# Synthesis of 1-Azatriene Complexes of Tungsten: Metal-Promoted Ring-Opening of Dihydropyridine

**DOI:** 10.1021/acs.organomet.4c00108

**Published:** 2024-04-30

**Authors:** Jonathan D. Dabbs, Megan N. Ericson, Diane A. Dickie, W. Dean Harman

**Affiliations:** Department of Chemistry, University of Virginia, Charlottesville, Virginia 22904, United States

## Abstract

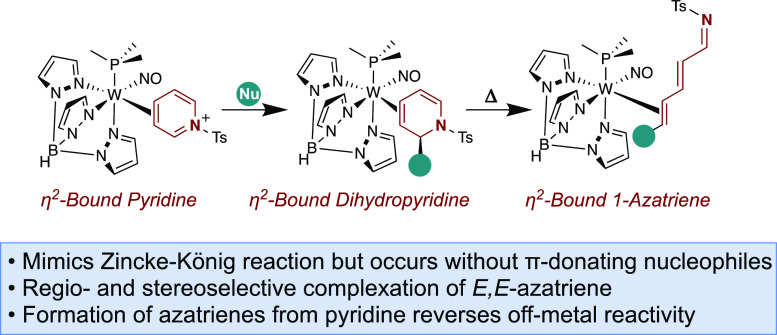

For nearly a century, chemists have explored how transition-metal complexes can affect the physical and chemical properties of linear conjugated polyenes and heteropolyenes. While much has been written about higher hapticity complexes (η^4^–η^6^), less is known about the chemistry of their η^2^ analogues. Herein, we describe a general method for synthesizing 5,6-η^2^-(1-azatriene) tungsten complexes via a 6π-azaelectrocyclic dihydropyridine ring-opening that is promoted by the π-basic nature of {WTp(NO)(PMe_3_)}. This study includes detailed spectroscopic and crystallographic data for the η^2^-dihydropyridine and η^2^-1-azatriene complexes, both of which were prepared as single regio- and stereoisomers.

## Introduction

**Figure 1:**
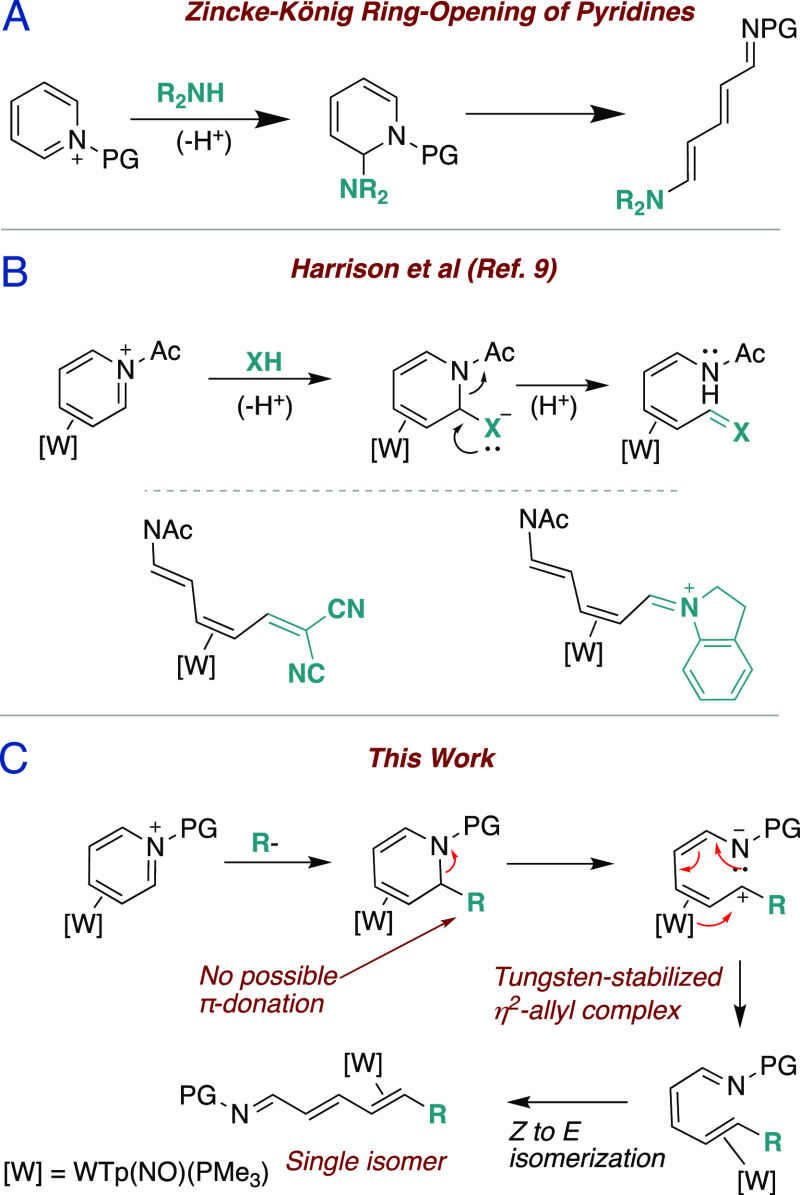
(A) Generic representation of the Zincke-König reaction. (B) Tungsten-mediated Zincke-König reaction with a C or N π-donor (X). (C) Proposed electrocyclization featuring an η^2^-allyl intermediate.

Since Reihlen and co-workers first reported the complex Fe(CO)_3_(η^4^-1,3-butadiene) in 1930,^1^ chemists have been fascinated by the ability of transition metals to influence the physical and chemical properties of conjugated polyenes.^2^ The vast majority of these studies have focused on η^4^-, η^5^-, and η^6^-coordinated polyenes, with some of the most significant developments historically involving iron.^2,3^ By comparison, much less is known about the properties of their η^2^-bound counterparts. Of the few complexes reported that involve nonaromatic polyenes, most are cyclooctatetraene complexes of Mn,^4,5^ Cu,^6^ and Ni.^7^ One reason for this dearth of linear η^2^-polyene complexes is the increased likelihood of constitutional- and stereoisomers, particularly if the metal complex is chiral.^8^ As an example, a complex formed between 1,3-pentadiene and an asymmetric metal complex can form up to 24 isomers (12 pairs of enantiomers).^8^ Thus, we were intrigued when we unexpectedly discovered that certain η^2^-dihydropyridine tungsten complexes could undergo an electrocyclic ring-opening to generate single diastereomers (racemic mixture) of η^2^-coordinated linear trienes and azatrienes (Figure 1B).^9^ This Zinke-König-like ring scission was possible when a substituent (**X**) alpha to the nitrogen was capable of π-donation, thereby stabilizing the carbocation generated by C–N ring scission. We questioned whether it was possible that the π-donating properties of tungsten alone could promote such a ring-scission, without the need for a π-donor substituent. The ring-opening could occur via an η^2^-allyl species that, following an allyl shift,^10^ would form an azatriene complex with the metal fragment intact (Figure 1C). If the dihydropyridine (DHP) complex could be prepared as a single regio- and stereoisomer, a single regio- and stereoisomer of the azatriene could result.

**Figure 2:**
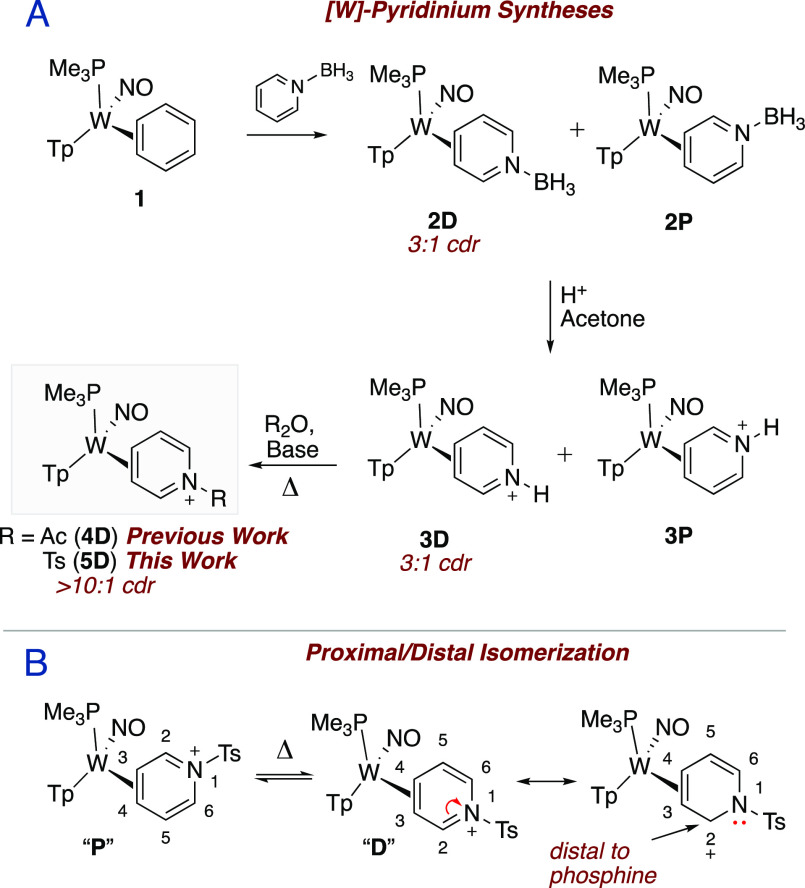
(A) Previously reported synthesis of **4D** and the analogous synthesis of **5D** from **1**. (B) Heat-induced isomerization of **5P** to **5D**.

Previous efforts have demonstrated that a complex of {WTp(NO)(PMe_3_)} ([W]; Tp = trispyrazolylborate) and *N*-acetylpyridinium triflate (**4D**) is a valuable precursor to functionalized tetrahydropyridines,^11−13^ isoquinuclidines,^14^ and trienyl enamides.^9^ This complex (**4D**) is generated by the initial exchange of WTp(NO)(PMe_3_)(benzene) (**1**) with pyridine-borane to form complex **2** as a mixture of distal (**D**) and proximal (**P**) coordination diastereomers (3:1 ratio; Figure 2A).^15^ This mixture then undergoes oxidative BH_3_-removal in acidic acetone to form the parent complex, [WTp(NO)(PMe_3_)(η^2^-pyridinium)]OTf (**3D** and **3P**). Owing to metal–ligand backbonding,^16^ the pyridine nitrogen is highly nucleophilic, allowing for its rapid acetylation in the presence of acetic anhydride. Fortuitously, gently heating this solution (55 °C) improves the coordination diastereomer ratio (cdr) from 3:1 to 10:1, thus maximizing the diastereomer with partial positive charge distal to the PMe_3_ (Figure 2, Panel B).^10^ This work demonstrates the tosylation of **4** via an analogous process that enriches before subsequently undergoing an array of nucleophilic additions followed by a heat-induced ring opening to form azatrienes η^2^-bound to a [W] fragment.

## Results and Discussion

While the acetylpyridinium complex **4D** has shown broad synthetic utility,^9,11−14^ the corresponding C2-alkylated DHP complexes (i.e., no potential to π donate) failed to undergo the ring-opening shown in Figure 1. Hence, we sought a more electron-withdrawing functional group for the nitrogen that could still effect high DHP stereoselectivity (cdr). We settled on the *N*-tosylated pyridinium complex **5D** to conduct our investigation.

In a procedure analogous to the synthesis of the *N*-acetylated complex (**4**), Ts_2_O was stirred in a solution of **3** and 1,2-dichloroethane. Upon addition of lutidine, a change in the *J*_WP_ (^31^P NMR spectrum; ^183^W) from ∼294 Hz (consistent with **3D**) to 279 and 286 Hz, indicated that tosylation had occurred.^17^ After 10 min at room temperature, the cdr had changed from 3:1 to ∼6:1 (^31^P NMR). Similar to its acylated derivative **4D**, the distal stereoisomer (**5D**) is thermodynamically favored and can be maximized through mild heating (55 °C) (Figure 2B).^10^ The resulting 11:1 mixture would reach as high as 20:1 (^1^H NMR) after undergoing an aqueous workup and precipitation. The structural assignment of this compound was supported with 2D NMR and high-resolution mass spectrometry (HRMS) data and yields for **5D** were as high as 89% when carried out on a multigram scale (10 g). Of note, the ^1^H and ^13^C NMR data indicated that the counterions triflate (–OTf) and tosylate (–OTs) were both present in a mixture in the final product. A cyclic voltammogram demonstrated that this complex has an *E*_pa_ of +1.18 V (*N*,*N*-dimethylacetamide (DMA), 100 mV/s), just slightly positive of the acetylated analogue, **4D**.^15^ However, the ν_NO_ values for **4D** and **5D** (FTIR/ATR) are 1611 and 1607 cm^–1^, respectively, an observation that together with the electrochemical data suggests that the tosyl group on **5D** is not significantly more electron-withdrawing than the acetyl group on **4D**. Fortunately, this feature would ultimately not preclude our intended goal (vide infra).

### Nucleophilic Additions to the DHP

**Figure 3:**
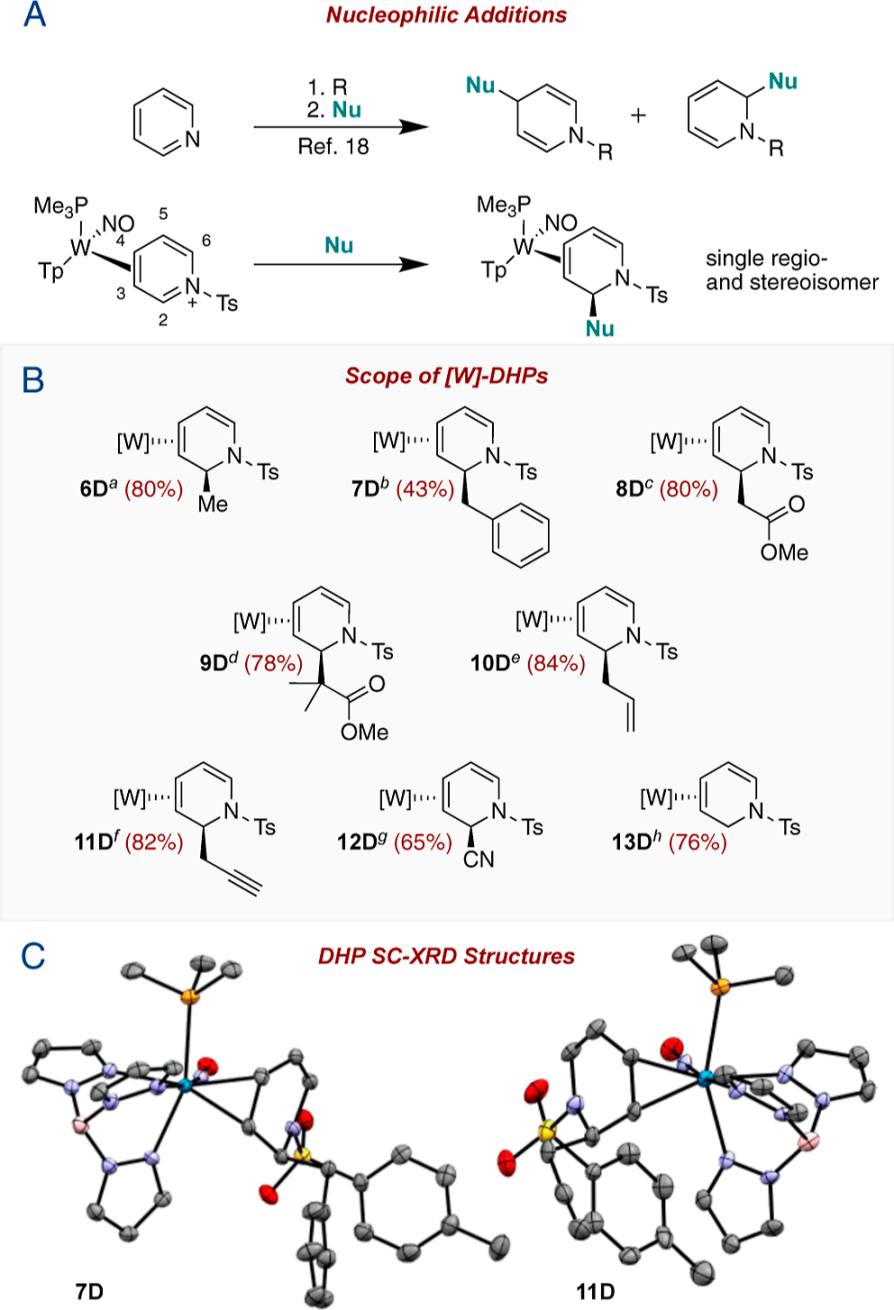
(A) Pyridinium salts (R = electron-withdrawing group) undergo selective addition reactions when coordinated to [W]. (B) DHP complexes **6D–13D** are generated by incorporating various nucleophiles to **5D** at C2 anti to the metal. All syntheses were conducted at room temperature under an inert N_2_ atmosphere. ^a^ZnMe_2_, THF. ^b^BnMgCl, THF. ^c^MBA, Zn°, THF. ^d^MTDA, TEA, DCM. ^e^allyl bromide, Zn°, THF. ^f^propargyl bromide, Zn°, THF. ^g^NaCN, MeOH. ^h^NaBH_4_, MeOH. (C) ORTEP/ellipsoid diagrams of **7D** and **11D** were acquired via SC-XRD.

The protection of the η^2^-bound pyridine with a tosyl group activates the iminium carbon for a wide range of chemoselective nucleophilic addition reactions. Nucleophilic addition to *N*-activated pyridinium salts is well-established,^18^ and while a wide range of 1,2-DHPs can be prepared via this method, these syntheses can be fraught with regioselectivity issues when the pyridine lacks a directing group (Figure 3A). In the case of complex **5**, the coordination of the pyridinium at C3 and C4 removes the possibility of C4 addition, and the bulky nature of the tungsten fragment [W] exclusively directs these nucleophiles anti to the metal at the C2 carbon.^19^

A range of carbon nucleophiles were chosen for the synthesis of DHP complexes shown in Figure 3. Yields of DHP complexes **6D**–**13D** range from 43–84%, the compounds being synthesized as racemic mixtures (Figure 3B). Nucleophiles successfully added included dimethylzinc (**6D**), benzyl magnesium chloride (BnMgCl) (**7D**), methyl bromoacetate (MBA) with zinc powder (**8D**), and methyl trimethylsilyl dimethylketene acetal (MTDA) (**9D**). Barbier reactions were conducted in the syntheses of **10D** and **11D**, using allyl bromide and propargyl bromide, respectively. Finally, cyanide and hydride were incorporated through the addition of NaCN and NaBH_4_ (**12D** and **13D**). The structures of **6D**, **7D**, and **11D** were determined via single-crystal X-ray diffraction (SC-XRD; Figure 3C), confirming their regio- and stereochemical assignments.

### DHP Ring Opening

**Figure 4:**
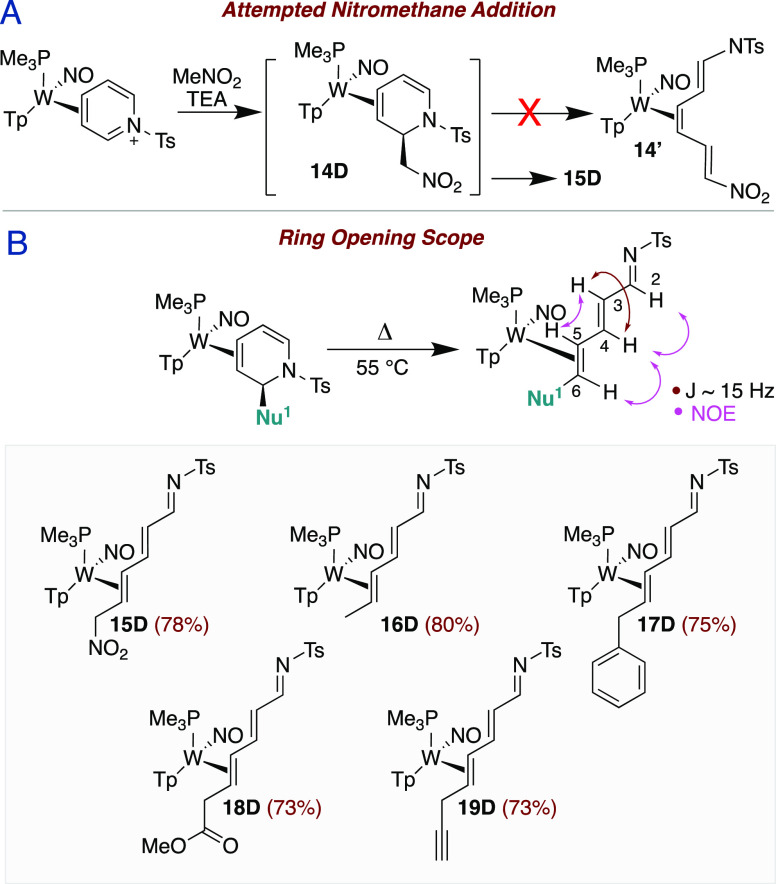
(A) Synthesis of **15D** via the **14D** intermediate. (B) Scope of DHP ring-opening to form **15D**–**19D**.

In addition to the successful preparations of DHP complexes shown in Figure 3, we attempted to synthesize a 2-nitromethyl-substituted DHP complex (**14D**). Using reaction conditions identical to those reported by Harrison et al. for the analogous acetyl-pyridinium system,^19^ excess nitromethane and triethylamine (TEA) were combined in a DCM solution of **5D** and stirred for 40 min. ^1^H NMR spectra revealed the presence of a lone diamagnetic complex characterized by several unexpected signals including a downfield doublet at 8.60 ppm and two doublet-of-doublets at 7.29 and 6.27 ppm. We anticipated that deprotonation alpha to the nitro-group might occur (**14′** in Figure 4) analogous to what Harrison et al. observed for malononitrile (Figure 1B). However, a full 2D NMR analysis indicated that **15D** was instead the desired 1-azatriene complex **15D** (Figure 4A). Unfortunately, paramagnetic impurities were also present and attempts to cleanly generate **15D** or **14D** by modulating the solvent, base, equivalence, temperature, or time were ineffective. However, other DHP complexes, including **6D**–**8D** and **11D**, were found to undergo this ring-opening with gentle heating (55 °C for 24 h), forming the desired 1-azatrienes **16D**–**19D** (Figure 4B). These compounds were characterized by similar downfield ^1^H NMR features as observed for **15D** but could be cleanly generated. Crystal structures of **17D** and **19D** not only confirm the proposed azadiene connectivity but show an E,E azatriene stereochemistry. This is consistent with H–H coupling for the uncoordinated alkene (∼15 Hz) and NOE data, which supports a trans-vicinal-hydrogen orientation (Figure 4). Bond lengths for the azatriene ligand are unremarkable, being largely in agreement with the crystal data of organic analogues^20−23^ but with a slight shortening (∼0.02 Å) of the C4–C5 and C2–C3 bonds. This observation is consistent with metal-backbonding into the azadiene π-system.^24^

**Figure 5:**
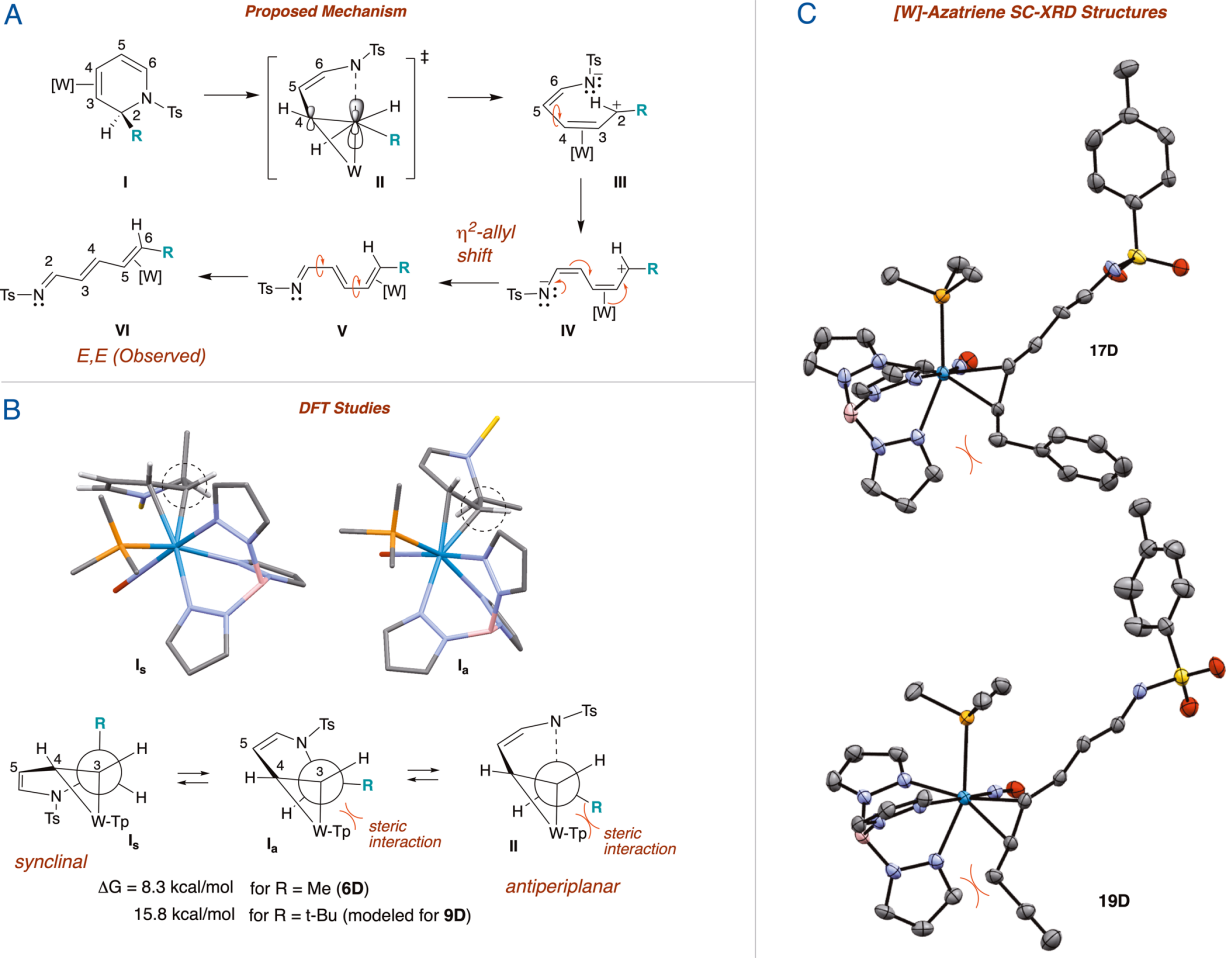
(A) Proposed mechanism for achieving E/E stereochemistry (B) Newman projection showing development of E stereochemistry and steric strain for large R. (C) ORTEP (50% ellipsoids) of **17D** and **19D**.

We anticipate that the driving force behind this reaction is the generation of a 1-azatriene ligand, which is an excellent π-acceptor for the π-basic [W] fragment.^16^ For example, DFT calculations indicate that the conversion of **6D** to **16D** is −2.8 kcal/mol. Typically, the reverse reaction—the electrocyclic ring closure— is the spontaneous reaction,^25^ unless the nitrogen has a strong withdrawing group and there is a π-donating substituent at C2 of DHP (see Figure 1).^26^ For example, DFT calculations indicate that conversion of the dihydropyridine ligand of **6D** is 0.7 kcal/mol more stable than the azatriene ligand of **16D** (Supporting Information). The purported mechanism behind this transformation (Figure 5) involves breaking of the C2–N bond (**I** → **III**) followed by 6π-electron-rearrangement concomitant with the “η^2^-allyl shift”^10^ to render an η^2^-azatriene complex with the terminal alkene (C5, C6) η^2^-bound to the metal. Breaking the C2–N bond likely requires a conformational change from the synclinal conformation observed in crystal structures (**I**_**s**_ in Figure 5, Panel B) to an antiperiplanar transition state (**II** in Panel B) that allows the developing p orbital on C2 to align with the W–C3–C4 π system.^10^ Between the ground-state conformation and the transition state lies a higher energy conformation (**I**_**a**_) that approximates the transition state. From this purported geometry, the R substituent on C2 (which was anti to the tungsten in the dihydropyridine **I**) is brought into a trans configuration relative to C4 (Figure 5, Panel B; **III**). The rearrangement from **IV** to **V** also locks in the trans stereochemistry of the C4–C5 bond (labeled as C3–C4 in the product 1-azatriene). Thus, a single stereoisomer of each azatriene complex is observed. We note that while the ester **8D** readily ring-opens, the bulkier analogue **9D** is stable, even with prolonged (∼3 days) heating at elevated temperatures (∼80 °C). A plausible explanation for this observation is a steric interaction in the requisite antiperiplanar conformation (**I**_**a**_ in Figure 5) between the geminal dimethyl moiety and the [W] fragment. Approximating the R group of **9D** with a *t*-butyl group, DFT calculations find that the conformation leading to the transition state is 15.7 kcal/mol above the synclinal ground state, whereas for R = Me (i.e., **6D**), the energetic cost is only 8.3 kcal/mol (Figure 5). This steric interaction can also be viewed in the crystal structure of **17D**, where R = Bn (see Figure 5, Panel C).

Interestingly, **12D** and **13D** fail to readily ring-open; even after prolonged periods (∼3 days) of elevated heating (∼80 °C), other than starting material, only decomposition products were observed. This observation is thought to be a result of the reduced hyperconjugation that would stabilize the positive charge at C2 of the allyl species **III** in Figure 5 and, hence, the transition state **II**.^10^ Furthermore, when a 6:1 cdr mixture of **7D** and **7P** was heated overnight (55 °C), a crude ^1^H NMR spectrum showed that only the distal form (**7D**) ring-opened to form **17D**, while the proximal isomer (**7P**) remained unreacted. Previous studies have demonstrated a thermodynamic preference to orient carbenium carbons distal to the PMe_3_.^10^ This experiment suggests that the asymmetric unit {WTp(NO)(PMe_3_)} stabilizes the transition state (**II** in Figure 5) of distal DHP complexes significantly more than for the proximal diastereomers.

Attempts to oxidatively decomplex the 1-azatriene complex **18D** using CAN, DDQ, or O_2_/silica were unsuccessful;^11,27^ CAN and DDQ both resulted in observable degradation of the complex with no identifiable free organics, while O_2_/silica rendered only starting material.

Several noteworthy comparisons can be drawn to other instances of DHP ring-opening. Zincke first observed this reactivity when combining a secondary amine with a dinitrophenylpyridinium salt. Other instances of azatrienes spontaneously formed from substituted DHP salts involve 1,2-DHPs substituted at C2 with other heteroatoms,^28^ aryl or alkyne R groups,^29,30^ or amino groups^31^ that serve as π donors (vide supra).^26^ Examples of metal-coordinated azatrienes are rare and almost always involve sigma coordination through a heteroatom. As an example, Cui et al.^21^ combined 2-picoline-*N*-oxide with a magnesium hydride complex and observed two different ring-opening products, depending on the solvent. These *N*-oxoazatrienes were bound through the oxygen. Wolczanski et al. demonstrated that pyridine could be opened to a binuclear niobium alkylidine complex.^32^ Several reports of η^4^-azatrienes have emerged, but they are cyclic and typically involve a larger π system.^33−35^ The only previous report of an η^2^-bound azatriene that we are aware of involves the complex MoTp(NO)(DMAP)(*N*-methyltrifluoropicolinium), which upon hydride reduction to the corresponding DHP and exposure to triflic acid (HOTf), evolves into a Mo-coordinated 1-azatriene analogous to those in Figure 4.^24^ The CF_3_ group of DHP and protonation of nitrogen were shown to be essential for the ring-opening to occur. The lack of azatriene formation from *N*-acetyled [W]-DHPs remains mysterious. While our own data is ambiguous (vide supra), Piettre and co-workers have made a strong case that sulfonyl groups are more withdrawing than acetyl groups.^36^ As stated earlier, a more withdrawing sulfonyl group would be better suited for stabilizing the proposed intermediates **III** and **IV** (Figure 5A).

## Concluding Remarks

Over several decades, our research group has endeavored to demonstrate the ability of an η^2^-coordinated transition metal (Os, Re, Mo, W) to effect organic transformations of aromatic molecules not accessible by conventional synthetic methods.^3,16,37,38^ An impediment to investigating parallel chemistry with linear polyenes has been in the preparation of such complexes as single regio- and stereoisomers. For example, {WTp(NO)(PMe_3_)} and the 1-azaheptatriene ligand of **16D** could form 12 different isomers (racemic) in which the metal was dihapto-coordinated.^8^ Many of these isomers would likely form with competitive rates and would have similar free energies,^8,16^ hence one would need to rely on tedious separation methods or fortuitous solubility differences to obtain a single isomer. This study outlines a possible strategy for forming such linear polyene complexes as single isomers, starting with an aromatic precursor where metal linkage isomerization is facile^16^ and then ring-opening to prepare the linear polyene system. Analogous approaches could be envisioned with pyrroles, furans, diazenes, and even cyclic polyenes of the form C_*n*_H_*n*_ (e.g., benzene, cycloheptatrienyl, cyclooctatetrene), where initial isomer formation can be minimized.^16^ Efforts in our lab continue toward these goals.

## Supporting Information Available

Synclinal and antiperiplanar conformations of tBu and Me, and organic ligands of 6D and16D (XYZ)
^1^H and ^13^C NMR spectra of selected compounds and crystallographic information for compounds **5D**–**19D** (PDF)

## Supplementary Material

om4c00108_si_001.xyz
