# Supplementary material for: Synthesis of 1-Azatriene Complexes of Tungsten: Metal-Promoted Ring-Opening of Dihydropyridine
Source: Organometallics. 2024 Apr 30;43(9):1051–6. doi: 10.1021/acs.organomet.4c00108 (PMC11094799; doi:10.1021/acs.organomet.4c00108)
Supplement: Supplementary file 2 — om4c00108_si_002.pdf [file om4c00108_si_002.pdf]

Supporting Information

Synthesis of 1-Azatriene Complexes of Tungsten: Metal-promoted Ring-Opening of Dihydropyridine

Jonathan D. Dabbs, Megan N. Ericson, Diane A. Dickie, and W. Dean Harman*

Department of Chemistry, University of Virginia, Charlottesville, Virginia 22904, United States

Table of Contents

General Information (S1-S3)

**S1**	**Supplementary Title Page, Table of Contents**	
**S2**	**General Methods**	
**S3**	**Abbreviations**	

Compound Synthetic Methodologies and Characterizations (S4-S17)

**S4**	**Synthesis and Characterization of [W] (*N*-tosyl) pyridinium Complex (5D)**
**S5-S12**	**Synthesis and Characterization of [W] 1,2-DHP Complexes (6D-13D)**
**S13-S17**	**Synthesis and Characterization of [W] 1-azatriene Complexes (15D-19D)**

NMR Spectroscopy (S18-S33)

**S18**	**5D**	**^{1}H & ^{13}C NMRs (Figures S1 and S2)**
**S19**	**6D**	**^{1}H & ^{13}C NMRs (Figures S3 and S4)**
**S20**	**7D**	**^{1}H & ^{13}C NMRs (Figures S5 and S6)**
**S21**	**8D**	**^{1}H & ^{13}C NMRs (Figures S7 and S8)**
**S22-S24**	**9D**	**^{1}H, ^{13}C, NOESY, COSY, HSQC, HMBC NMRs (Figures S9-S14)**
**S25**	**10D**	**^{1}H & ^{13}C NMRs (Figures S15 and S16)**
**S26**	**11D**	**^{1}H & ^{13}C NMRs (Figures S17 and S18)**
**S27**	**12D**	**^{1}H & ^{13}C NMRs (Figures S19 and S20)**
**S28**	**13D**	**^{1}H & ^{13}C NMRs (Figures S21 and S22)**
**S29**	**15D**	**^{1}H & ^{13}C NMRs (Figures S23 and S24)**
**S30**	**16D**	**^{1}H & ^{13}C NMRs (Figures S25 and S26)**
**S31**	**17D**	**^{1}H & ^{13}C NMRs (Figures S27 and S28)**
**S32**	**18D**	**^{1}H & ^{13}C NMRs (Figures S29 and S30)**
**S33**	**19D**	**^{1}H & ^{13}C NMRs (Figures S31 and S32)**

SC-XRD Data (S34-S39)

**S34**	**6D**	**ORTEP Diagram & SC-XRD Data (Figure S33 and Table S1)**
**S35**	**7D**	**ORTEP Diagram & SC-XRD Data (Figure S34 and Table S2)**
**S36**	**11D**	**ORTEP Diagram & SC-XRD Data (Figure S35 and Table S3)**
**S37**	**13P**	**ORTEP Diagram & SC-XRD Data (Figure S36 and Table S4)**
**S38**	**17D**	**ORTEP Diagram & SC-XRD Data (Figure S37 and Table S5)**
**S39**	**19D**	**ORTEP Diagram & SC-XRD Data (Figure S38 and Table S6)**

Computational Methods (S40) **(Table S7)**

General Methods:

NMR spectra were obtained on an 800 MHz spectrometer. Chemical shifts are referenced to tertramethylsilane (TMS) utilizing residual ^{1}H or ^{13}C signals of the deuterated solvents as internal standards. Chemical shifts are reported in ppm and coupling constants (*J*) are reported in hertz (Hz). Infrared Spectra (IR) were recorded on a spectrometer as a glaze on a diamond anvil ATR assembly, with peaks reported in cm^{-1}. Electrochemical experiments were performed under a nitrogen atmosphere. Most cyclic voltammetric (CV) data were recorded at ambient temperature at 100 mV/s, unless otherwise noted, with a standard three electrode cell from +1.25 V to -1.25 V with a platinum working electrode, *N,N*-dimethylacetamide (DMA) or acetonitrile solvent, and tetrabutylammonium hexafluorophosphate (TBAH) electrolyte (~1.0 M). All potentials are reported versus the normal hydrogen electrode (NHE) using cobaltocenium hexafluorophosphate ($E_{1/2}$ = -0.78 V, -1.75 V) or ferrocene ($E_{1/2}$ = 0.55 V) as an internal standard. Peak separation of all reversible couples was less than 100 mV. All synthetic reactions were performed in a glovebox under a dry nitrogen atmosphere unless otherwise noted. All solvents were purged with nitrogen prior to use. Deuterated solvents were used as received from Cambridge Isotopes.

NMR assignments of all compounds were determined using 2D NMR methods including NOESY, COESY, HMBC, and HSQC. When possible, pyrazole (Pz) protons of the (trispyrazolyl) borate (Tp) ligand were uniquely assigned (e.g., "PzB3") using two-dimensional NMR data (see Figure S1). If unambiguous assignments were not possible, Tp protons were labeled as "Pz3/5 or Pz4". All *J* values for Pz protons are 2 (±0.4) Hz. BH peaks (around 4-5 ppm) in the ^{1}H NMR spectra are not assigned due to their quadrupole broadening; however, confirmation of the BH group is provided by IR data (around 2500 cm^{-1}).

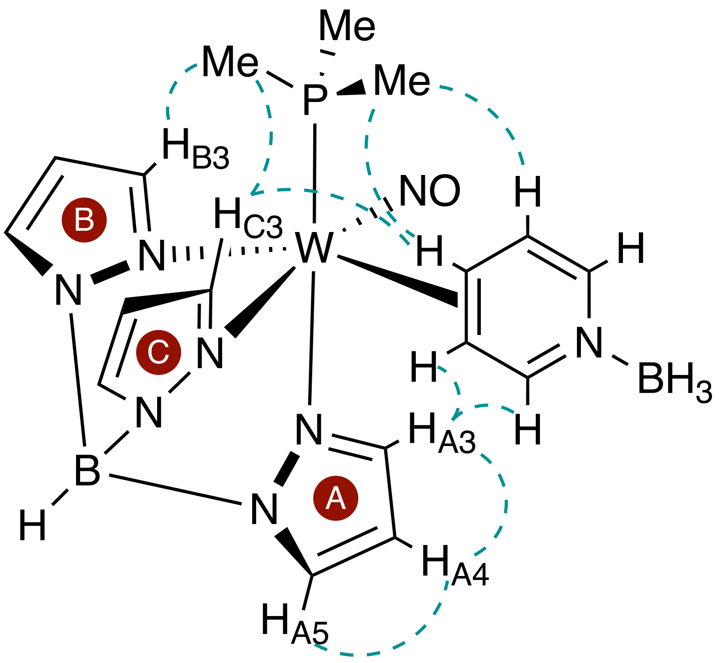

Abbreviations:

Anhydrous Magnesium Sulfate	$MgSO_4$
Acetonitrile	MeCN
Benzyl Magnesium Chloride	BnMgCl
Dichloroethane	DCE
Dichloromethane	DCM
Diethyl ether	Et_2O
Diethylzinc	$ZnEt_2$
Dimethoxyethane	DME
Dimethylacetamide	DMA
Dimethylzinc	$ZnMe_2$
Lithium Dimethyl malonate	LiDiMM
Methanol	MeOH
Sodium Borohydride	$NaBH_4$
Sodium Cyanide	NaCN
Sodium tertbutoxide	NaOtBu
Saturated Aqueous Sodium Bicarbonate	$NaHCO_3$
Tetrahydrofuran	THF
Triethylamine	TEA
Triflic Acid	TfOH
Trimethylsilyl dimethylketene acetal	MTDA

COMPOUND SYNTHETIC METHODOLOGIES AND CHARACTERIZATIONS

Synthesis and characterization of WTp(NO)(PMe$_3$)(η^2-(*N*-tosyl)-pyridinium) (OTf) (5D)

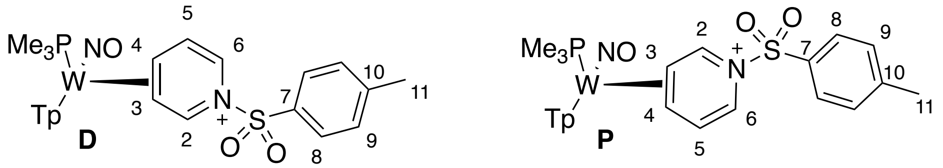

Methanesulfonic anhydride (10.51 g, 32.20 mmol), **3** (8.34 g, 11.39 mmol), and DCE (100 mL) were charged to a flame-dried 250 mL round-bottom flask with a 1-inch stir bar. Lutidine (2.31 g, 21.56 mmol) was added to initiate the reaction, and the flask was submerged in a pre-heated oil bath set to 55 °C. After stirring the solution for ~2 hours, the solution was diluted with 150 mL of DCM and added to a separatory funnel. This solution was washed 3x with 200 mL of saturated aqueous $NaHCO_3$. The organic layer was isolated and set aside. The combined aqueous layers were combined and back extracted with 50 mL of DCM to prevent product loss. The organic layers were combined in a single flask and dried with anhydrous $MgSO_4$. This powder was then filtered off into a 60 mL coarse porosity fritted funnel and washed with DCM. The dried organic layers were then reduced *in vacuo* down to dryness. The residue in the flask was redissolved in minimal DCM (approximately 10 mL). The solution was then slowly added to 500mL of stirring Et_2O. An orange precipitate formed immediately and was allowed to stir for ~10 minutes to ensure total precipitation. This powder was collected on a 60 mL medium porosity frit and washed 2x with 30 mL of ether. This powder was dried in a desiccator under vacuum for ~30 minutes. (7.74 g, 76% yield)

^{1}H NMR (CD_2Cl_2, δ, 25 °C):
D: 9.05 (d, J = 6.0 Hz, 1H, H2), 7.97 (d, J = 8.5 Hz, 2H, H8), 7.94 (d, J = 2.2, 2H (2 overlapping doublets), Tp3/5), 7.93 (d, J = 2.0 Hz, 1H, Tp3/5), 7.91 (d, J = 2.4 Hz, 1H, Tp3/5), 7.76 (d, J = 2.5 Hz, 1H, Tp3/5), 7.66 (d, J = 2.5 Hz, 1H, Tp3/5), 7.49 (d, J = 8.3 Hz, 1H, H9), 6.54 (ddd, J = 1.8, 5.5, 7.6, 1H, H5), 6.46 (t, J = 2.3 Hz, 1H, Tp4), 6.43 (dd, J = 1.6, 7.6, 1H, H6), 6.42 (t, J = 2.3 Hz, 1H, Tp4), 6.40 (t, J = 2.4 Hz, 1H, Tp4), 4.13 (dt, J = 6.1, 12.4 Hz, 1H, H4), 3.00 (td, J = 1.8, 6.4 Hz, 1H, H3), 2.49 (s, 3H, H11), 1.23 (d, J_{PH} = 9.2 Hz, 9H, PMe_3).
P: 9.57 (d, J = 5.4 Hz, 1H, H2), [Tp doublets and triplets buried] 6.72 (t, J = 6.8 Hz, 1H, H5), 6.21 (dd, J = 1.2, 7.4, 1H, H6), 4.36 (q, J = 6.5 Hz, 1H, H3), 2.76 (t, J = 6.5 Hz, 1H, H4), 2.50 (s, 3H, H11), 1.23 (d, J_{PH} = 8.8 Hz, 9H, PMe_3).

^{13}C NMR (CD_2Cl_2, δ, 25 °C):
D: 167.2 (C2), 148.5 (C7), 146.4 (Tp3/5), 145.9 (Tp3/5), 138.6 (Tp3/5), 138.4 (Tp3/5), 137.6 (Tp3/5), 133.1 (C10), 131.5 (C9), 129.0 (C8), 126.6 (Tp3/5), 124.1 (d, J_{PC} = 2.9 Hz, C5), 114.8 (C6), 108.2 (Tp4), 108.2 (Tp4), 107.8 (Tp4), 67.1 (d, J_{PC} = 13.9 Hz, C4), 66.6 (C3), 22.0 (C11), 13.2 (d, J_{PC} = 31.1 Hz, 3C, PMe_3).
P: signals too weak to assign.

IR: ν(NO) = 1607 cm^{-1}
CV (DMA; 100 mV/s): $E_{p,a}$ = +1.18 V (NHE)
HRMS (ESI) m/z: [M]$^+$ Calcd for $C_{24}H_{31}BN_8O_3PSW^+$ 737.1574; Found 737.1575

Synthesis and characterization of WTp(NO)(PMe_3)(η^2-(*N*-tosyl)-2-(methyl)-1,2-dihydropyridine) (6D)

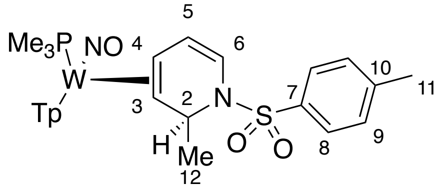

An oven dried 4-dram vial and stir pea were set up on a stir plate and charged with **5D** (4 g, 4.46 mmol). This was then diluted with dry THF (20 mL), and 1.2 M $ZnMe_2$ (in THF) was added to the solution (14.8 mL, 17.8 mmol). This solution was then stirred for 15 minutes and then quenched with a few drops of saturated aqueous $NaHCO_3$. The solution was diluted with 150 mL of DCM and added to a separatory funnel. This solution was washed 3x with 200 mL of sat. aq. $NaHCO_3$. The organic layer was isolated and set aside. The combined aqueous layers were combined and back extracted with 50 mL of DCM to prevent product loss. The organic layers were combined in a single flask and dried with anhydrous $MgSO_4$. This powder was then filtered off into a 60 mL coarse porosity fritted funnel and washed with DCM. The dried organic layers were then reduced *in vacuo* to dryness. The residue in the flask was redissolved in minimal DCM (approximately 5 mL). The solution was then slowly added to 200mL of stirring pentane. A tan precipitate formed immediately and was allowed to stir for ~10 minutes to ensure total precipitation. This powder was collected on a 60 mL medium porosity frit and washed 2x with 30 mL of pentane. This powder was dried in a desiccator under vacuum for ~30 minutes (2.70 g, 80% yield).

^{1}H NMR (CD_3CN, δ, 25 °C): 8.03 (d, J = 1.80 Hz, 1H, PzA3), 8.02 (d, J = 2.0 Hz, 1H, PzB3), 7.84 (d, J = 2.4 Hz, 1H, PzB5), 7.81 (d, J = 8.3 Hz, 2H, H8), 7.81 (d, J = 2.2 Hz, 1H, PzC5), 7.75 (d, J = 2.4 Hz, 1H, PzA5), 7.35 (d, J = 8.7 Hz, 2H, H9), 7.32 (d, J = 2.2 Hz, 1H, PzC3), 6.36 (t, J = 2.2 Hz, 1H, PzB4), 6.25 (t, J = 2.2 Hz, 1H, PzA5), 6.24 (t, J = 2.2 Hz, 1H, PzC4), 5.91 (d, J = 7.9 Hz, 1H, H6), 5.75 (dd, J = 5.1, 7.9 Hz, 1H, H5), 4.57 (q, J = 6.1, 12.2 Hz, 1H, H2), 2.77 (ddd, J = 5.1, 10.5, 13.7 Hz, H4), 2.39 (s, 3H, H11), 1.23 (d, J = 10.5 Hz, 1H, H3), 1.21 (d, J_{PH} = 8.5 Hz, 9H, PMe_3), 0.96 (d, J = 6.1 Hz, 3H, H12).

^{13}C NMR (CD_3CN, δ, 25 °C): 144.3 (C7), 144.2 (d, J_{PC} = 2.2 Hz, PzB3), 144.1 (PzA3), 141.5 (PzC3), 140.1 (C10), 137.7 (PzC5), 137.3 (PzB5), 136.9 (PzA5), 130.6 (2C, C9), 128.4 (2C, C8), 115.7 (C6), 114.3 (d, J_{PC} = 2.7 Hz, C5), 107.4 (PzB4), 107.2 (PzC4), 107.0 (PzA4), 67.5 (C3), 51.4 (C2), 43.7 (d, J_{PC} = 10.3 Hz, C4), 22.3 (C12), 21.5 (C11), 13.6 (d, J_{PC} = 28.4 Hz, 3C, PMe_3).

IR: ν(NO) = 2492 cm^{-1}
CV (DMA; 100 mV/s): $E_{p,a}$ = +0.44 V (NHE)
SC-XRD Data on S34

Synthesis and characterization of WTp(NO)(PMe_3)(η^2-(*N*-tosyl)-2-(benzyl)-1,2-dihydropyridine) (7D)

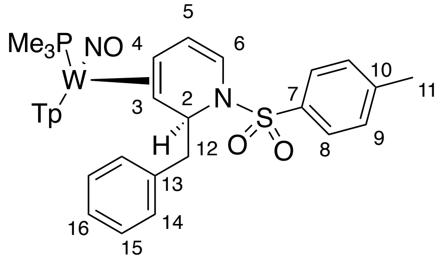

An oven dried 4-dram vial and stir pea were set up on a stir plate and charged with **5D** (198 mg, 0.22 mmol). This was then diluted with dry DME (3 mL), and BnMgCl (1.0 M in dry THF) was added to the solution (0.75 mL, 0.75 mmol). This solution was then stirred for 25 minutes and quenched with sat. aq. $NaHCO_3$. The solution was diluted with 150 mL of DCM and added to a separatory funnel. This solution was washed 3x with 200 mL of saturated aqueous $NaHCO_3$. The organic layer was isolated and set aside. The combined aqueous layers were combined and back extracted with 50 mL of DCM to prevent product loss. The organic layers were combined in a single flask and dried with anhydrous $MgSO_4$. This powder was then filtered off into a 60 mL coarse porosity fritted funnel and washed with DCM. The dried organic layers were then reduced *in vacuo* to dryness. The residue in the flask was redissolved in minimal DCM (approximately 5 mL). The solution was then slowly added to 100 mL of stirring pentane. A tan precipitate formed immediately and was allowed to stir for ~10 minutes to ensure total precipitation. This powder was collected on a 60 mL medium porosity frit and washed 2x with 30 mL of pentane. This powder was dried in a desiccator under vacuum for ~30 minutes (78 mg, 43% yield).

^{1}H NMR (CD_2Cl_2, δ, 25 °C): 8.06 (d, J = 1.8 Hz, 1H, PzA3), 8.04 (d, J = 1.9 Hz, 1H, PzB3), 7.92 (d, J = 8.3 Hz, 2H, H8), 7.71 (d, J = 2.3 Hz, 1H, PzB5), 7.63 (d, J = 2.2 Hz, 1H, PzC5), 7.53 (d, J = 2.4 Hz, 1H, PzA5), 7.34 (d, J = 8.3 Hz, 2H, H9), 7.14 (d, J = 2.0 Hz, 1H, PzC3), 7.03 (t, J = 7.5 Hz, 2H, H15), 6.94-6.98 (m, 13H, H14/16), 6.31 (t, J = 2.2 Hz, 1H, PzB4), 6.14 (t, J = 2.2 Hz, 1H, PzA4), 6.13 (t, J = 2.2 Hz, 1H, PzC4), 6.06 (d, J = 7.8 Hz, 1H, H6), 5.76 (dd, J = 5.1, 7.8 Hz, 1H, H5), 4.68 (d, J =10.3 Hz, 1H, H2), 2.87 (dd, J = 10.3, 12.6 Hz, 1H, H12'), 2.74 (ddd, J = 4.9, 10.6, 13.5 Hz, 1H, H4), 2.61 (dd, J = 3.0, 12.6 Hz, 1H, H12), 2.39 (s, 3H, H11), 1.45 (d, J = 10.3 Hz, 1H, H3), 1.20 (d, J_{PH} = 8.3 Hz, 1H, PMe_3).

^{13}C NMR (CD_2Cl_2, δ, 25 °C): 143.6 (C7), 143.5 (PzA3),143.4 (d, J_{PC} = 1.7 Hz, PzB3), 140.1 (C13), 139.9 (PzC3), 139.5 (C10), 136.7 (PzC5), 136.2 (PzB5), 135.7 (PzA5), 130.0 (2C, C9), 129.8 (2C, C14), 128.2 (2C, C15), 127.9 (2C, C8), 125.8 (C16), 115.9 (C6), 114.1 (d, J_{PC} = 3.2 Hz, C5), 106.6 (PzB4), 106.1 (PzA4/C4), 106.0 (PzA4/C4), 62.4 (d, J_{PC} = 1.6 Hz, C3), 56.7 (C2), 43.8 (d, J_{PC} = 10.3 Hz, C4), 41.7 (C12), 21.6 (C11), 13.6 (d, J_{PC} = 27.9 Hz, 3C, PMe_3).

SC-XRD Data on S35

Synthesis and characterization of WTp(NO)(PMe_3)(η^2-(*N*-tosyl)-2-(methylacetate)-1,2-dihydropyridine) (8D)

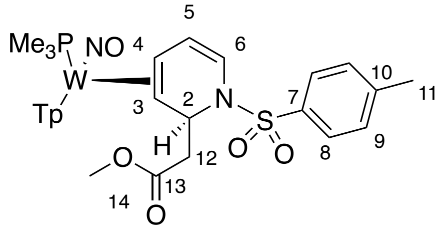

Methyl bromoacetate (224 mg, 1.46 mmol), **5D** (956 mg, 1.07 mmol), and THF (12 mL) were charged to a flame-dried 50 mL round-bottom flask with a 1-inch stir bar. Zinc powder (281 mg, 4.30 mmol) was added to initiate the reaction, and the heterogenous solution was stirred for 1 hour. The zinc was removed by filtering the solution through a celite plug set up in a 60 mL coarse porosity frit with 1 inch of celite, which was then washed with residual DCM to prevent loss of product. The solution was diluted with 150 mL of DCM and added to a separatory funnel. This solution was washed 3x with 200 mL of saturated aqueous $NaHCO_3$. The organic layer was isolated and set aside. The combined aqueous layers were combined and back extracted with 50 mL of DCM to prevent product loss. The organic layers were combined in a single flask and dried with anhydrous $MgSO_4$. This powder was then filtered off into a 60 mL coarse porosity fritted funnel and washed with DCM. The dried organic layers were then reduced *in vacuo* to dryness. The residue in the flask was redissolved in minimal DCM (approximately 5 mL). The solution was then slowly added to 200mL of stirring pentane. A tan precipitate formed immediately and was allowed to stir for ~10 minutes to ensure total precipitation. This powder was collected on a 60 mL medium porosity frit and washed 2x with 30 mL of pentane. This powder was dried in a desiccator under vacuum for ~30 minutes (690 mg, 80% yield).

^{1}H NMR (CD_2Cl_2, δ, 25 °C): 8.05 (d, *J* = 1.9 Hz, 1H, PzB3), 7.90 (d, *J* = 1.6 Hz, 1H, PzA3), 7.86 (d, *J* = 8.3 Hz, 2H, H8), 7.75 (d, *J* = 2.4 Hz, 1H, PzB5), 7.72 (d, *J* = 2.4 Hz, 1H, PzC5), 7.63 (d, *J* = 2.4 Hz, 1H, PzA5),7.32 (d, *J* = 8.2 Hz, 2H, H9), 7.21 (d, *J* = 1.9 Hz, 1H, PzC3), 6.33 (t, *J* = 2.2 Hz, 1H, PzB4), 6.20 (t, *J* = 2.2 Hz, 1H, PzA4), 6.19 (t, *J* = 2.1 Hz, 1H, PzC4), 6.01 (d, *J* = 7.8 Hz, 1H, H6), 5.72 (dd, *J* = 5.1, 7.8 Hz, 1H, H5), 4.90 (d, *J* = 9.8 Hz, 1H, H2), 3.38 (s, 3H, H14), 2.70 (m, 2H, H4 & H12), 2.40 (s, 3H, H11), 2.36 (dd, *J* = 8.4, 15.1 Hz, 1H, H12'), 1.46 (d, *J* = 10.7 Hz, 1H, H3), 1.20 (d, J_{PH} = 8.4 Hz, 9H, PMe_3).

^{13}C NMR (CD_2Cl_2, δ, 25 °C): 172.5 (C13), 143.8 (C7), 143.7 (PzA3), 143.5 (d, J_{PC} = 2.2 Hz, PzB3),140.2 (PzC3), 138.4 (C10), 136.9 (PzC5), 136.4 (PzB5), 135.8 (PzA5), 130.0 (2C, C9), 128.2 (2C, C8), 115.4 (C6), 113.7 (d, J_{PC} = 3.0 Hz, C5), 106.6 (PzB4), 106.3 (PzC4), 106.0 (PzA4), 64.3 (d, J_{PC} = 2.2 Hz, J_{WC} = 33.0 Hz, C3), 52.1 (C2), 51.4 (C14), 43.5 (d, J_{PC} = 10.7 Hz, C4), 41.3 (C12), 21.7 (C11), 13.4 (d, J_{PC} = 27.8 Hz, 3C, PMe_3).

IR: ν(NO) = 1570 cm^{-1}
CV (DMA; 100 mV/s): $E_{p,a}$ = +0.50 V (NHE)

EA for $C_{27}H_{36}BN_8O_5PSW$:
Calcd: (%C, 40.02), (%H, 4.48), (%N, 14.05)
Found: (%C, 39.95), (%H, 4.40), (%N, 14.20)

Synthesis and characterization of WTp(NO)(PMe_3)(η^2-(*N*-tosyl)-2-(*α*,*α*-dimethyl-methylacetate)-1,2-dihydropyridine) (9D)

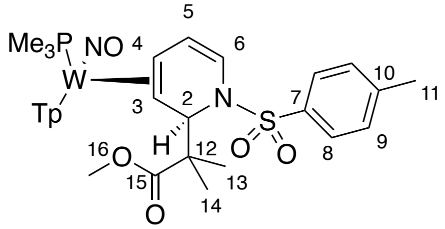

An oven dried 4-dram vial and stir pea were set up on a stir plate and charged with **5D** (1.004 g, 1.13 mmol). This was then diluted with DCM (11 mL), and MTDA (412 mg, 2.36 mmol) was added to the solution. This solution was stirred for 2 hours and quenched with minutes and the solution was quenched with TEA (311 mg, 3.07 mmol). The reaction was diluted with 150 mL of DCM and added to a separatory funnel. This solution was washed 3x with 200 mL of saturated aqueous $NaHCO_3$. The organic layer was isolated and set aside. The combined aqueous layers were combined and back extracted with 50 mL of DCM to prevent product loss. The organic layers were combined in a single flask and dried with anhydrous $MgSO_4$. This powder was then filtered off into a 60 mL coarse porosity fritted funnel and washed with DCM. The dried organic layers were then reduced *in vacuo* to dryness. The residue in the flask was redissolved in minimal DCM (approximately 5 mL). The solution was then slowly added to 200mL of stirring pentane. A tan precipitate formed immediately and was allowed to stir for ~10 minutes to ensure total precipitation. This powder was collected on a 60 mL medium porosity frit and washed 2x with 30 mL of pentane. This powder was dried in a desiccator under vacuum for ~30 minutes (740 mg, 78% yield).

^{1}H NMR (CD_2Cl_2, δ, 25 °C): 8.41 (d, J = 1.9 Hz, 1H, PzA3), 8.05 (d, J = 1.9 Hz, 1H, PzB3), 7.80 (d, J = 8.3 Hz, 2H, H8), 7.75 (d, J = 2.4 Hz, 1H, PzB5), 7.72 (d, J = 2.3 Hz, 1H, PzC5), 7.66 (d, J = 2.4 Hz, 1H, PzA5), 7.26 (d, J = 8.2 Hz, 2H, H9), 7.24 (d, J = 1.8 Hz, 1H, PzC3), 6.33 (t, J = 2.4 Hz, 1H, PzA4), 6.32 (t, J = 2.3 Hz, 1H, PzB4), 6.22 (t, J = 2.2 Hz, 1H, PzC4), 6.04 (d, J = 7.8 Hz, 1H, H6), 5.67 (dd, J = 5.0, 7.8 Hz, 1H, H5), 5.34 (s, 1H, H2), 2.87 (s, 3H, H16), 2.81 (ddd, J = 5.0, 10.8, 14.1 Hz, 1H, H4), 2.39 (s, 3H, H11), 1.25 (s, 3H, H14), 1.19 (d, J_{PH} = 8.3 Hz, 9H, PMe_3), 1.00 (d, J = 10.8 Hz, 1H, H3), 0.89 (s, 3H, H13)

^{13}C NMR (CD_2Cl_2, δ, 25 °C): 177.4 (C15), 144.3 (PzA3), 143.2 (d, J_{PC} = 1.7 Hz, PzB3), 143.1 (C7), 140.7 (C10), 140.0 (PzC3), 136.7 (PzC5), 136.4 (PzB5), 135.8 (PzA5), 129.6 (2C, C9), 127.9 (2C, C8), 119.1 (C6), 115. (d, J_{PC} = 2.7 Hz, C5), 106.6 (PzA4/B4), 106.4 (PzC4), 106.1 (PzA4/B4), 59.9 (2C, C2 and C3), 54.7 (C12), 51.0 (C16), 44.7 (d, J_{PC} = 10.6 Hz, C4), 25.9 (C13), 21.6 (C11), 19.8 (C14), 13.4 (d, J_{PC} = 27.9 Hz, 3C, PMe_3).

IR: ν(NO) = 1577 cm^{-1}
CV (DMA; 100 mV/s): $E_{p,a}$ = +0.46 V (NHE)
Unable to obtain EA, SC-XRD, or HRMS data for compound. Full 2D NMR provided below.

Synthesis and characterization of WTp(NO)(PMe$_3$)(η^2-(*N*-tosyl)-2-(allyl)-1,2-dihydropyridine) (10D)

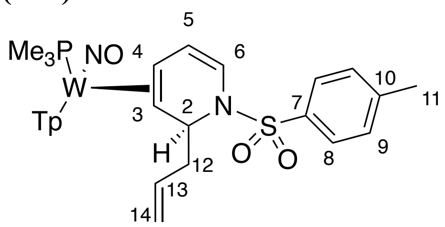

Allyl bromide (350 mg, 2.89 mmol), **5D** (999 mg, 1.11 mmol), and THF (8 mL) were charged to a flame-dried 50 mL round-bottom flask with a 1-inch stir bar. Zinc powder (323 mg, 4.93 mmol) was added to initiate the reaction, and the heterogenous solution was stirred for 2 hours. The zinc was removed by filtering the solution through a celite plug set up in a 60 mL coarse porosity frit with 1 inch of celite, which was then washed with residual DCM to prevent loss of product. The solution was diluted with 150 mL of DCM and added to a separatory funnel. This solution was washed 3x with 200 mL of saturated aqueous $NaHCO_3$. The organic layer was isolated and set aside. The combined aqueous layers were combined and back extracted with 50 mL of DCM to prevent product loss. The organic layers were combined in a single flask and dried with anhydrous $MgSO_4$. This powder was then filtered off into a 60 mL coarse porosity fritted funnel and washed with DCM. The dried organic layers were then reduced *in vacuo* to dryness. The residue in the flask was redissolved in minimal DCM (approximately 5 mL). The solution was then slowly added to 200mL of stirring pentane. A tan precipitate formed immediately and was allowed to stir for ~10 minutes to ensure total precipitation. This powder was collected on a 60 mL medium porosity frit and washed 2x with 30 mL of pentane. This powder was dried in a desiccator under vacuum for ~30 minutes (730 mg, 84% yield).

^{1}H NMR (CD_2Cl_2, δ, 25 °C): 8.14 (d, J = 1.9 Hz, 1H, PzA3), 8.05 (d, J = 1.9 Hz, 1H, PzB3), 7.83 (d, J = 8.3 Hz, 2H, H8), 7.75 (d, J = 2.4 Hz, 1H, PzB5), 7.73 (d, J = 2.2 Hz, 1H, PzC5), 7.64 (d, J = 2.4 Hz, 1H, PzA5), 7.32 (d, J = 8.3 Hz, 2H, H9), 7.22 (d, J = 2.2 Hz, 1H, PzC3), 6.32 (t, J = 2.2 Hz, 1H, PzB4), 6.23 (t, J = 2.2 Hz, 1H, PzA4), 6.20 (t, J = 2.2 Hz, 1H, PzC4), 6.01 (d, J = 7.8 Hz, 1H, H6), 5.68 (dd, J = 5.0, 7.8 Hz, 1H, H5), 5.60 (m, 1H, H13), 4.72 (d, J = 10.2 Hz, 1H, H14), 4.70 (d, J = 17.9 Hz, 1H, H14'), 4.53 (d, J = 9.5 Hz, 1H, H2), 2.73 (ddd, J = 5.0, 10.6, 13.5 Hz, 1H, H4), 2.41 (s, 3H, H11), 2.41 (m, 1H, H12), 1.99 (m, 1H, H12'), 1.61 (d, J = 10.6 Hz, 1H, H3), 1.21 (d, J_{PH} = 8.3 Hz, 9H, PMe$_3$).]

^{13}C NMR (CD_2Cl_2, δ, 25 °C): 143.7 (PzA3), 143.5 (d, J_{PC} = 1.5 Hz, PzB3), 143.5 (C7), 140.2 (PzC3), 139.6 (C10), 136.8 PzB5), 136.6 (C13), 136.3 (PzC5), 135.8 (PzA5), 129.9 (2C, C9), 127.8 (2C, C8), 116.3 (C14), 116.2 (C6), 113.7 (d, J_{PC} = 3.0 Hz, C5), 106.6 (PzB4), 106.3 (PzC4), 106.2 (PzA4), 62.8 (td, J_{WP} = 1.5, 16.6 Hz, C3), 55.2 (C2), 43.7 (d, J_{PC} = 10.4 Hz, C4), 41.0 (C12), 21.6 (C11), 13.6 (d, J_{PC} = 27.9 Hz, 3C, PMe$_3$).

IR: ν(NO) = 1561 cm^{-1}
CV (DMA; 100 mV/s): $E_{p,a}$ = +0.46 V (NHE)

EA for $C_{27}H_{36}BN_8O_3PSW$:
Calcd: (%C, 41.67), (%H, 4.66), (%N, 14.40)
Found: (%C, 41.97), (%H, 4.81), (%N, 14.24)

Synthesis and characterization of WTp(NO)(PMe$_3$)(η^2-(*N*-tosyl)-2-(propargyl)-1,2-dihydropyridine) (11D)

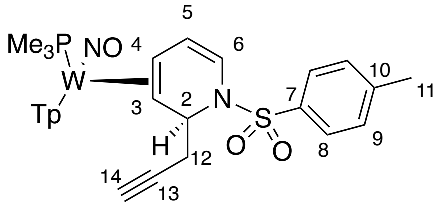

Propargyl bromide (220 mg, 1.85 mmol), **5D** (935 mg, 1.04 mmol), and THF (10 mL) were charged to a flame-dried 50 mL round-bottom flask with a 1-inch stir bar. Zinc powder (391 mg, 5.98 mmol) was added to initiate the reaction, and the heterogenous solution was stirred for 2 hours. The zinc was removed by filtering the solution through a celite plug set up in a 60 mL coarse porosity frit with 1 inch of celite, which was then washed with residual DCM to prevent loss of product. The solution was diluted with 150 mL of DCM and added to a separatory funnel. This solution was washed 3x with 200 mL of saturated aqueous $NaHCO_3$. The organic layer was isolated and set aside. The combined aqueous layers were combined and back extracted with 50 mL of DCM to prevent product loss. The organic layers were combined in a single flask and dried with anhydrous $MgSO_4$. This powder was then filtered off into a 60 mL coarse porosity fritted funnel and washed with DCM. The dried organic layers were then reduced *in vacuo* to dryness. The residue in the flask was redissolved in minimal DCM (approximately 5 mL). The solution was then slowly added to 200mL of stirring pentane. A tan precipitate formed immediately and was allowed to stir for ~10 minutes to ensure total precipitation. This powder was collected on a 60 mL medium porosity frit. (666 mg, 82% yield).

Cleanup Procedure: extra clean material obtained by dissolving the obtained powder was then added to 100 mL of stirring diethyl ether and allowed to stir until homogeneous (~20 minutes). 150 mL of pentanes were then added, causing a fine powder to precipitate out. This powder was then collected on a frit, and has a slight beige color. This powder is highly pure.

^{1}H NMR (CD_2Cl_2, δ, 25 °C): 8.06 (d, J = 1.7 Hz, 1H, PzB3), 8.00 (d, J = 1.6 Hz, 1H, PzA3), 7.84 (d, J = 8.3 Hz, 2H, H8), 7.76 (d, J = 2.3 Hz, 1H, PzB5), 7.74 (d, J = 2.1 Hz, 1H, PzC5), 7.65 (d, J = 2.3 Hz, 1H, PzA5), 7.33 (d, J = 8.0 Hz, 2H, H9), 7.25 (d, J = 1.9 Hz, 1H, PzC3), 6.33 (t, J = 2.2 Hz, 1H, PzB4), 6.22 (t, J = 2.2 Hz, 2H, PzA4 & PzC4), 5.96 (d, J = 7.7 Hz, 1H, H6), 5.73 (dd, J = 5.1, 7.7 Hz, 1H, H5), 4.60 (d, J = 9.5 Hz, 1H, H2), 2.74 (ddd, J = 5.1, 10.7, 13.5 Hz, 1H, H4), 2.56 (ddd, J = 2.7, 10.0, 16.1 Hz, 1H, H12), 2.42 (s, 3H, H11), 2.15 (dt, J = 2.7, 16.1 Hz, 1H, H12'), 1.96 (d, J = 10.7 Hz, 1H, H3), 1.71 (t, J = 2.7 Hz, 1H, H14), 1.20 (d, J_{PH} = 8.3 Hz, 1H, PMe$_3$).

^{13}C NMR (CD_2Cl_2, δ, 25 °C): 143.8 (C7), 143.7 (PzA3), 143.5 (PzB3), 140.3 (PzC3), 138.8 (C10), 136.9 (PzC5), 136.4 (PzB5), 135.9 (PzA5), 130.1 (2C, C9), 127.9 (2C, C8), 115.1 (C6), 114.2 (C5), 106.6 (PzB4), 106.3 (PzC4), 106.2 (PzA4), 83.1 (C13), 69.8 (C14), 62.7 (J_{WC} = 33.9 Hz, C3), 54.6 (C2), 43.6 (d, J_{PC} = 10.6 Hz, C4), 25.5 (C12), 21.7 (C11), 13.5 (d, J_{PC} = 28.0 Hz, 3C, PMe$_3$).

IR: ν(NO) = 1607 cm^{-1}
CV (DMA; 100 mV/s): $E_{p,a}$ = +1.18 V (NHE)
SC-XRD Data on S36

Synthesis and characterization of WTp(NO)(PMe$_3$)(η^2-(*N*-tosyl)-2-(carbonitrile)-1,2-dihydropyridine) (12D)

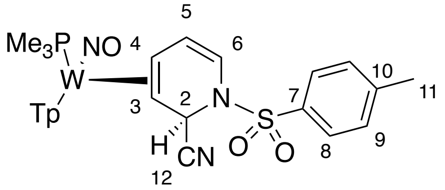

An oven dried 4-dram vial and stir pea were set up on a stir plate and charged with **5D** (963 mg, 1.07 mmol). This was then diluted with MeOH (12 mL), and NaCN was added to the solution (189 mg, 3.86 mmol). This solution was then stirred for 2.5 hours. The solution was diluted with 150 mL of DCM and added to a separatory funnel. This solution was washed 3x with 200 mL of saturated aqueous $NaHCO_3$. The organic layer was isolated and set aside. The combined aqueous layers were combined and back extracted with 50 mL of DCM to prevent product loss. The organic layers were combined in a single flask and dried with anhydrous $MgSO_4$. This powder was then filtered off into a 60 mL coarse porosity fritted funnel and washed with DCM. The dried organic layers were then reduced *in vacuo* to dryness. The residue in the flask was redissolved in minimal DCM (approximately 5 mL). The solution was then slowly added to 200mL of stirring pentane. A tan precipitate formed immediately and was allowed to stir for ~10 minutes to ensure total precipitation. This powder was collected on a 60 mL medium porosity frit and washed 2x with 30 mL of pentane. This powder was dried in a desiccator under vacuum for ~30 minutes (534 mg, 65% yield).

^{1}H NMR (CD_2Cl_2, δ, 25 °C): 8.04 (d, *J* = 1.9, 1H, PzB3), 8.03 (d, *J* = 1.9 Hz, 1H, PzA3), 7.83 (d, *J* = 8.4 Hz, 2H, H8), 7.77 (d, *J* = 2.4 Hz, 1H, PzB5), 7.76 (d, *J* = 2.2 Hz, 1H, PzC5), 7.69 (d, *J* = 2.4 Hz, 1H, PzA5), 7.38 (d, *J* = 8.2 Hz, 2H, H9), 7.26 (d, *J* = 2.2 Hz, 1H, PzC3), 6.35 (t, *J* = 1.9 Hz, 1H, PzB4), 6.30 (t, *J* = 2.2 Hz, 1H, PzA4), 6.24 (t, *J* = 2.4 Hz, 1H, PzC4), 6.05 (d, *J* = 7.8 Hz, 1H, H6), 5.95 (dd, *J* = 5.3, 7.8 Hz, 1H, H5), 5.26 (m, 1H, H2), 2.77 (ddd, *J* = 5.3, 10.3, 12.9 Hz, 1H, H4), 2.44 (s, 3H, H11), 1.77 (d, *J* = 10.3, 1H, H3), 1.25 (d, J_{PH} = 8.5 Hz, 9H, PMe$_3$).

^{13}C NMR (CD_2Cl_2, δ, 25 °C): 144.7 (C7), 143.8 (d, J_{PC} =1.9 Hz, PzB3), 143.0 (PzA3), 140.5 (PzC3), 137.1 (PzC5), 136.8 (PzB5), 136.2 (PzA5), 135.8 (C10), 130.1 (2C, C9), 128.1 (2C, C8), 121.3 (C12), 115.5 (d, J_{PC} = 2.7 Hz, C6), 115.3 (C5), 106.8 (PzB4), 106.7 (PzA4), 106.6 (PzC4), 62.4 (d, J_{PC} = 3.0 Hz, J_{WC} = 33.8 Hz, C3), 47.1 (C2), 42.8 (d, J_{PC} = 11.2 Hz, C4), 21.7 (C11), 13.7 (d, J_{PC} = 28.6 Hz, 3C, PMe$_3$).

IR: ν(NO) = 1577 cm^{-1}
CV (DMA; 100 mV/s): $E_{p,a}$ = +0.66 V (NHE)

EA for $C_{25}H_{31}BN_9O_3PSW$:
Calcd: (%C, 39.34), (%H, 4.09), (%N, 16.52)
Found: (%C, 39.27), (%H, 4.02), (%N, 16.41)

Synthesis and characterization of WTp(NO)(PMe$_3$)(η^2-(*N*-tosyl)-1,2-dihydropyridine) (13D)

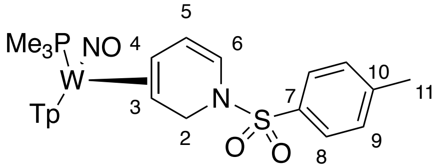

Two separate solutions were prepared in two 30 mL glass screw-cap test tubes. Tube A was comprised of **5D** (1.052 g, 1.17 mmol) dissolved in MeOH and Tube B was a heterogeneous slurry of $NaBH_4$ (125 mg, 3.30 mmol) in dry THF (10 mL). The tube B slurry was added to tube A, and the tube sat for 10 minutes (bubbling occurred). The solution was diluted with 150 mL of DCM and added to a separatory funnel. This solution was washed 3x with 200 mL of saturated aqueous $NaHCO_3$. The organic layer was isolated and set aside. The combined aqueous layers were combined and back-extracted with 50 mL of DCM to prevent loss of product. The organic layers were combined in a single flask and dried with anhydrous $MgSO_4$. This powder was then filtered off into a 60 mL coarse porosity fritted funnel and washed with DCM. The dried organic layers were then reduced *in vacuo* to dryness. The residue in the flask was redissolved in minimal DCM (approximately 5 mL). The solution was then slowly added to a stirring 100mL solution of pentane. A tan precipitate formed immediately and was allowed to stir for ~10 minutes to ensure total precipitation. This powder was collected on a 60 mL medium porosity frit and washed 2x with 30 mL of pentane. This powder was dried in a desiccator under vacuum overnight. (663 mg mg, 76% yield)

^{1}H NMR (CD_2Cl_2, δ, 25 °C): 8.04 (d, J = 1.8 Hz, 1H, PzB3), 7.98 (d, J = 1.6 Hz, 1H, PzA3), 7.75 (d, J = 2.2 Hz, 1H, PzB5), 7.74 (d, J = 8.2 Hz, 2H, H8), 7.73 (d, J = 2.2 Hz, 1H, PzC5), 7.66 (d, J = 2.3 Hz, 1H, PzA5), 7.32 (d, J = 8.1 Hz, 2H, H9), 7.23 (d, J = 1.8 Hz, 1H, PzC3), 6.33 (t, J = 2.2 Hz, 1H, PzB4), 6.25 (t, J = 2.1 Hz, 1H, PzB4), 6.20 (t, J = 2.2 Hz, 1H, PzC4), 6.10 (d, J = 7.9 Hz, 1H, H6), 5.75 (dd, J = 4.6, 7.9 Hz, 1H, H5), 4.42 (d, J = 11.2 Hz, 1H, H2), 4.10 (dd, J = 3.9, 11.2 Hz, 1H, H2'), 2.63 (ddd, J = 4.6, 10.3, 12.7 Hz, 1H, H4), 2.41 (s, 3H, H11), 1.39 (d, J = 10.3 Hz, 1H, H3), 1.23 (d, J_{PH} = 8.4 Hz, 9H, PMe_3).

^{13}C NMR (CD_2Cl_2, δ, 25 °C): 143.9 (d, J_{PC} = 1.9 Hz, PzB3), 143.6 (C7), 143.4 (PzA3), 140.5 (PzC3), 136.9 (PzC5), 136.5 (PzB5), 136.4 (C10), 135.9 (PzA5), 129.9 (2C, C9), 127.9 (2C, C8), 119.3 (C6), 115.3 (d, J_{PC} = 2.9 Hz, C5), 106.7 (PzB4), 106.4 (PzA4), 106.3 (PzC4), (C3 obscured behind CD_2Cl_2), 47.3 (C2), 46.0 (d, J_{PC} = 10.7 Hz, C4), 21.7 (C11), 14.0 (d, J_{PC} = 27.9 Hz, 3C PMe_3).

IR: ν(NO) = 1560 cm^{-1}
CV (DMA; 100 mV/s): $E_{p,a}$ = +0.47 V (NHE)
Proximal Form SC-XRD Data on S37

Synthesis and characterization of WTp(NO)(PMe$_3$)(η^2-(*NE*,3*E*,5*E*)-(*N*-tosyl)-(6-nitromethyl)-1-azatriene) (15D)

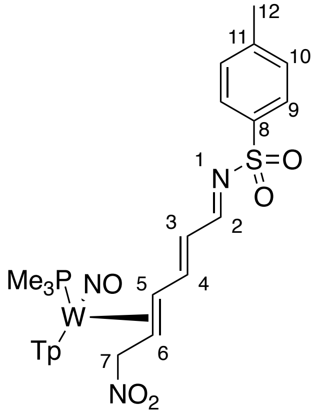

An oven-dried 4-dram vial was tared and charged with **5D** (102 mg, 0.13 mmol) and EtCN (1 mL). A separate dry 4-dram vial was added nitromethane (32 mg, 0.32 mmol) and TEA (21 mg, 0.34) along with EtCN(1 mL). The nitromethane/TEA solution was then added to the [W] solution, and the combined solution was stirred for 40 hrs. The reaction solution was then precipitated in 80 mL of stirring Et_2O, and the tan precipitate was collected on a 15 mL F frit. The frit was then dried in the desiccator overnight. (80 mg, 78%)

^{1}H NMR (CD_2Cl_2, δ, 25 °C): 8.58 (d, *J* = 9.9 Hz, 1H, H2), 8.05 (d, *J* = 1.9 Hz, 1H, Tp3/5), 8.04 (d, *J* = 1.9 Hz, 1H, Tp3/5), 7.83 (d, *J* = 2.3 Hz, 1H, Tp3/5), 7.82 (d, *J* = 2.4 Hz, 1H, Tp3/5), 7.76 (d, *J* = 8.3 Hz, 2H, H9), 7.70 (d, *J* = 2.4 Hz, 1H, Tp3/5), 7.53 (d, *J* = 2.1 Hz, 1H, Tp3/5), 7.33 (d, *J* = 8.3 Hz, 2H, H10), 7.27 (dd, *J* = 10.6, 14.8 Hz, 1H, H4), 6.40 (t, *J* = 2.3 Hz, 1H, Tp4), 6.36 (t, *J* = 2.3 Hz, 1H, Tp4), 6.35 (t, *J* = 2.3 Hz, 1H, Tp4), 6.27 (dd, *J* = 9.9, 14.8 Hz, 1H, H3), 3.74 (ddd, *J* = 1.4, 3.8, 12.8 Hz, 1H, H7'), 3.48 (t, *J* = 12.3, 1H, H7'), 3.40 (m, 1H, H5), 3.35 (m, 1H, H6), 2.43 (s, 3H, H12), 1.16 (d, J_{PH} = 8.7 Hz, 9H, PMe$_3$).

^{13}C NMR (CD_2Cl_2, δ, 25 °C): 174.3 (C4), 171.3 (C2), 144.3 (Tp3/5), 144.2 (d, J_{PC} = 1.8 Hz, Tp3/5), 144.1 (C8), 142.1 (Tp3/5), 138.0 (Tp3/5), 137.6 (C11), 137.2 (Tp3/5), 136.5 (Tp3/5), 130.0 (2C, C10), 127.7 (2C, C9), 120.2 (C3), 107.6 (Tp4), 107.5 (Tp4), 107.1 (Tp4), 82.1 (C7), 64.5 (d, J_{PC} = 2.0 Hz, H5), 60.5 (d, J_{PC} = 2.0 Hz, H6), 21.7 (C12), 12.7 (d, J_{PC} = 28.9 Hz, 3C, PMe$_3$).

HRMS (ESI) m/z: $[M]^{+H}$ Calcd for $C_{25}H_{34}BN_9O_5PSW^+$ 798.1738; Found 798.1744

Synthesis and characterization of WTp(NO)(PMe$_3$)(η^2-(*NE*,3*E*,5*E*)-(*N*-tosyl)-(6-methyl)-1-azatriene) (16D)

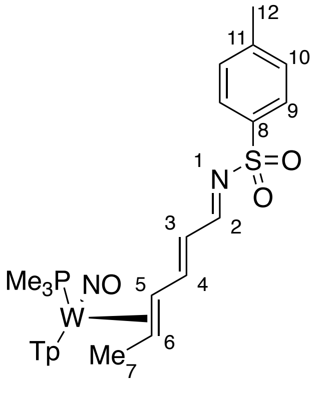

A 10 mL screw cap test tube was tared and charged with **6D** (76 mg, 0.09 mmol) and EtCN (3 mL). This tube was then added to a 55 °C mineral oil bath heated for 22 hours. The reaction solution was then precipitated in 80 mL of stirring Et_2O, and the yellow precipitate was collected on a 15 mL F frit. The frit was then dried in the desiccator overnight. (61 mg, 80%)

^{1}H NMR (CDCl$_3$, δ, 25 °C): 8.60 (d, J = 10.1 Hz, 1H, H2), 8.02 (d, J = 1.8 Hz, 1H, PzB3), 7.99 (d, J = 1.8 Hz, 1H, PzA3), 7.81 (d, J = 8.2 Hz, 1H, H9), 7.74 (d, J = 2.2 Hz, 2H, PzB5/C5), 7.61 (d, J = 2.3 Hz, 1H, PzA5), 7.43 (d, J = 1.9 Hz, 1H, PzC3), 7.28 (d, J = 8.2 Hz, 1H, H10), 7.24 (dd, J = 11.5, 14.6 Hz, 1H, H4), 6.32 (t, J = 2.2 Hz, 1H, PzB4),6.27 (t, J = 2.1 Hz, 1H, PzA4), 6.23 (t, J = 2.2 Hz, 1H, PzC4), 6.24 (dd, J = 10.1, 14.6 Hz, 1H, H3), 3.26 (dt, J = 7.8, 11.5 Hz, 1H, H5), 3.19 (m, 1H, H6), 2.40 (s, 3H, H12), 1.15 (d, J_{PH} = 8.3 Hz, 9H, PMe$_3$), 1.11 (d, J = 6.6 Hz, 3H, H7).

^{13}C NMR (CDCl$_3$, δ, 25 °C): 177.9 (C4), 171.1 (C2), 144.0 (PzA3), 143.7 (d, J_{PC} = 1.4 Hz,PzB3), 143.2 (C8), 141.9 (PzC3), 137.8 (C11), 136.8 (PzB5/PzC5), 136.3 (PzB5/PzC5), 135.4 (PzA4), 129.6 (C10), 127.5 (C9), 118.2 (C3), 106.9 (PzB4), 106.7 (PzA4), 106.0 (PzC4), 69.0 (d, J_{PC} = 6.3 Hz, C5), 65.7 (C6), 21.7 (C12), 17.2 (C7), 12.7 (d, J_{PC} = 27.9 Hz, PMe$_3$).

IR: ν(NO) = 1541 cm^{-1}
HRMS (ESI) m/z: [M]$^{+H}$ Calcd for $C_{25}H_{35}BN_8O_3PSW^+$ 753.1887; Found 753.1887

Synthesis and characterization of WTp(NO)(PMe$_3$)(η^2-(*NE*,3*E*,5*E*)-(*N*-tosyl)-(6-benzyl)-1-azatriene) (17D)

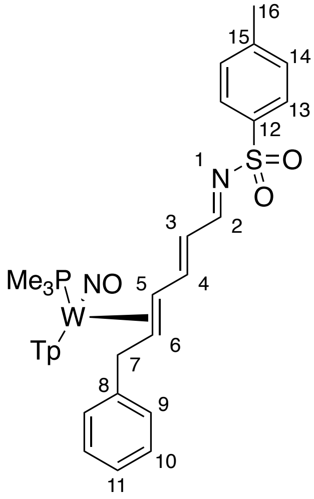

A 10 mL screw cap test tube was tared and charged with **7D** (15 mg, 0.02 mmol) and EtCN (3 mL). This tube was then added to a 55 °C mineral oil bath heated for 22 hours. The reaction solution was then precipitated in 80 mL of stirring Et$_2$O, and the yellow precipitate was collected on a 15 mL F frit. The frit was then dried in the desiccator overnight. (51 mg, 75%)

^{1}H NMR (CD$_2$Cl$_2$, δ, 25 °C): 8.51 (d, J = 10.1 Hz, 1H, H2), 8.04-8.05 (m, 2H, Tp3/5), 7.81-7.83 (m, 2H, Tp3/5), 7.72-7.75 (m, 3H, H13 & Tp3/5), 7.31 (d, J = 7.9 Hz, 2H, H14), 7.27 (dd, J = 11.6, 14.6 Hz, 1H, H4), 7.17 (t, J = 7.4 Hz, 2H, H10), 7.06 (t, J = 7.4 Hz, 1H, H11), 6.99 (d, J = 7.4 Hz, 2H, H9), 6.38 (t, J = 2.1 Hz, 1H, Tp4), 6.37 (t, J = 2.2 Hz, 1H, Tp4), 6.30 (t, J = 2.2 Hz, 1H, Tp4), 6.20 (dd, J = 10.1, 14.6 Hz, 1H, H3), 3.39-3.43 (m, 1H, H5), 3.27-3.31 (m, 1H, H6), 2.42 (s, 3H, H11), 2.12 (d, J = 14.8 Hz, 1H, H7), 1.93 (dd, J = 11.2, 14.8 Hz, 1H, H7), 1.18 (d, J_{PH} = 8.5 Hz, 9H, PMe$_3$).

^{13}C NMR (CD$_2$Cl$_2$, δ, 25 °C): 177.4 (C12), 171.2 (C2), 147.7 (C8), 144.0 (2C, Tp3/5), 143.8 (C4), 142.3 (Tp3/5), 138.1 (C15), 137.3 (Tp3/5), 136.8 (Tp3/5), 136.0 (Tp3/5), 129.9 (2C, C14), 128.5 (2C, C10), 128.2 (2C, C9), 127.6 (2C, C13), 125.7 (C11), 118.2 (C3), 107.2 (Tp4), 107.2 (Tp4), 106.4 (Tp4), 71.9 (C6), 67.8 (d, J_{PC} = 6.6 Hz, C5), 39.3 (C7), 21.7 (C16), 12.8 (d, J_{PC} = 28.5 Hz, 3C, PMe$_3$).

SC-XRD Data on S38

Synthesis and characterization of WTp(NO)(PMe$_3$)(η^2-(*NE*,3*E*,5*E*)-(*N*-tosyl)-(6-methylester)-1-azatriene) (18D)

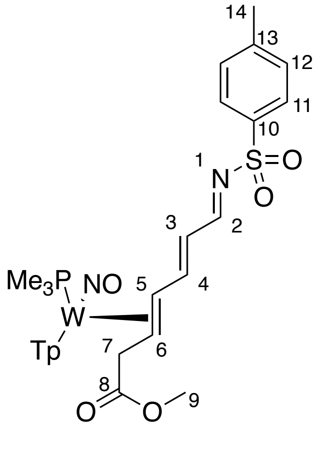

A 10 mL screw cap test tube was tared and charged with **8D** (30 mg, 0.10 mmol) and MeCN (3 mL). This tube was then added to a 55 °C mineral oil bath heated for 18 hours. The reaction solution was then precipitated in 80 mL of stirring Et_2O, and the yellow precipitate was collected on a 15 mL F frit. The frit was then dried in the desiccator overnight. (22 mg, 73%)

^{1}H NMR (CD_3CN, δ, 25 °C): 8.50 (d, J = 10.1Hz, 1H, H2), 8.05 (d, J = 2.0 Hz, 1H, PzB3), 8.00 (d, J = 2.0 Hz, 1H, PzA3, 7.90 (d, J = 2.4 Hz, 1H, PzA5), 7.89 (d, J = 2.4 Hz, 1H, PzC5), 7.77 (d, J = 2.3 Hz, 1H, PzB5), 7.73 (d, J = 8.2 Hz, 2H, H11), 7.63 (d, J = 2.2 Hz, 1H, PzC3), 7.43 (dd, J = 10.6, 14.6 Hz, 1H, H4), 7.37 (d, J = 8.2 Hz, 2H, H12), 6.40 (t, J = 2.2 Hz, 1H, PzB4), 6.34 (t, J = 2.3 Hz, 1H, PzA4), 6.32 (t, J = 2.2 Hz, 1H, PzC4), 6.14 (dd, J = 10.1, 14.6 Hz, 1H, H3), 3.47 (s, 3H, H9), 3.41 (m, 1H, H5), 3.22 (m, 1H, H6), 2.41 (s, 3H, H14), 1.68 (dd, J = 3.5, 15.6 Hz, 1H, H7'), 1.59 (dd, J = 11.1, 15.6 Hz, 1H, H7), 1.12 (d, J_{PH} = 8.7 Hz, 9H, PMe$_3$).

^{13}C NMR (CD_3CN, δ, 25 °C): 177.8 (C8), 177.5 (C4), 172.0 (C2), 144.9 (d, J_{PC} = 1.9 Hz, PzB3), 144.7 (PzA3), 144.6 (C10), 143.6 (PzC3), 139.0 (C13), 138.4 (PzC5), 137.8 (PzA5), 137.1 (PzB5), 130.7 (2C, C12), 127.9 (2C, C11), 117.7 (C3), 108.0 (PzA4), 107.9 (PzB4), 107.3 (PzC4), 67.0 (d, J_{PC} = 6.2 Hz, C5), 63.9 (C6), 51.8 (C9), 37.9 (C7), 21.6 (C14), 12.8 (d, J_{PC} = 29.0 Hz, 3C, PMe$_3$).

IR: ν(NO) = 1531 cm^{-1}
CV (DMA; 100 mV/s): $E_{p,a}$ = +0.85 V (NHE)
HRMS (ESI) m/z: $[M]^{+H}$ Calcd for $C_{27}H_{37}BN_8O_5PSW^+$ 811.1942; Found 811.1947

Synthesis and characterization of WTp(NO)(PMe$_3$)(η^2-(*NE*,3*E*,5*E*)-(*N*-tosyl)-(6-propargyl)-1-aztatriene) (19D)

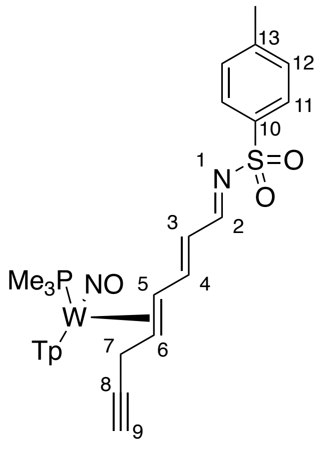

A 10 mL screw cap test tube was tared and charged with **11D** (30 mg, 0.10 mmol) and EtCN (3 mL). This tube was then added to a 55 °C mineral oil bath heated for 48 hours. The reaction solution was then precipitated in 80 mL of stirring Et_2O, and the yellow precipitate was collected on a 15 mL F frit. The frit was then dried in the desiccator overnight. (22 mg, 73%)

^{1}H NMR (CD_2Cl_2, δ, 25 °C): 8.56 (d, *J* = 10.0 Hz, 1H, H2), 8.02 (d, *J* = 1.6 Hz, 1H, Tp3/5), 8.01 (d, *J* = 1.6 Hz, 1H, Tp3/5), 7.78-7.82 (m, 2H, Tp3/5), 7.76 (d, *J* = 8.2 Hz, 2H, H11), 7.67 (d, *J* = 2.2 Hz, 1H, Tp3/5), 7.47 (d, *J* = 1.8 Hz, 1H, Tp3/5), 7.32 (d, *J* = 8.2 Hz, 2H, H12), 7.30 (dd, *J* = 11.1, 14.7 Hz, 1H, H4), 6.37 (t, *J* = 2.1 Hz, 1H, Tp4), 6.33 (t, *J* = 2.1 Hz, 1H, Tp4), 6.28 (t, *J* = 2.1 Hz, 1H, Tp4), 6.26 (dd, *J* = 10.1, 14.7 Hz, 1H, H3), 3.32 (dt, *J* = 7.5, 11.1 Hz, 1H, H5), 3.18-3.22 (m, 1H, H6), 2.42 (s, 3H, H14), 1.89 (t, *J* = 2.5 Hz, 1H, H9), 1.71 (d, *J* = 17.6 Hz, 1H, H7), 1.44 (d, *J* = 2.5, 11.1, 17.6 Hz, 1H, H7), 1.16 (d, J_{PH} = 8.5 Hz, 9H, PMe_3).

^{13}C NMR (CD_2Cl_2, δ, 25 °C): 177.1 (C4), 171.3 (C2), 144.2 (Tp3/5), 144.0 (Tp3/5), 143.8 (C10), 142.2 (Tp3/5), 138.1 (C13), 137.5 (Tp3/5), 136.9 (Tp3/5), 136.0 (Tp3/5), 129.9 (2C, C12), 127.6 (2C, C11), 118.7 (C3), 107.2 (2C, Tp4), 106.6 (Tp4), 89.8 (C8), 67.7 (C6), 67.6 (C9), 66.4 (d, J_{PC} = 7.0 Hz, C5), 21.7 (2C, C7 & C14), 12.8 (d, J_{PC} = 28.6 Hz, 3C, PMe_3).

HRMS (ESI) m/z: $[M]^{+H}$ Calcd for $C_{27}H_{35}BN_8O_3PSW^+$ 777.1887; Found 777.1895

SC-XRD Data on S39

NMR Spectroscopy:

Figure S1: ^{1}H NMR, 800 MHz, CD_2Cl_2, 25 °C, **5D**

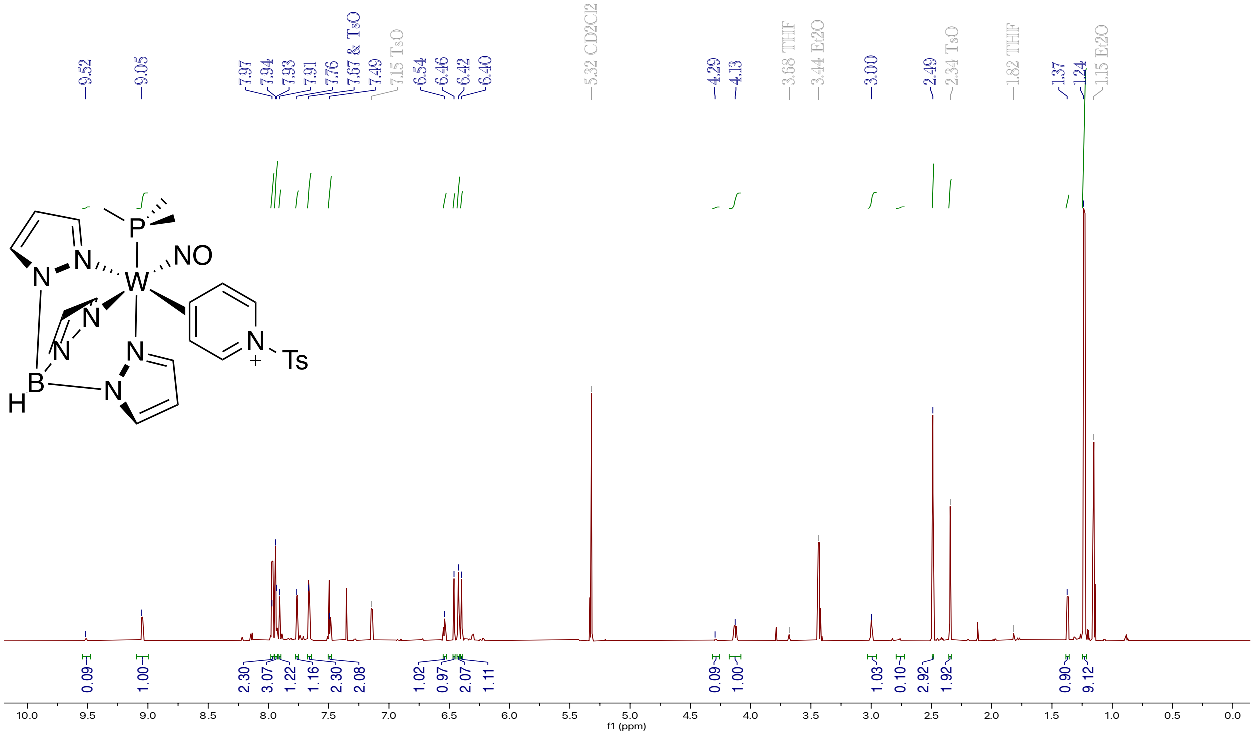

Figure S2: ^{13}C NMR, 200 MHz, CD_2Cl_2, 25 °C, **5D**

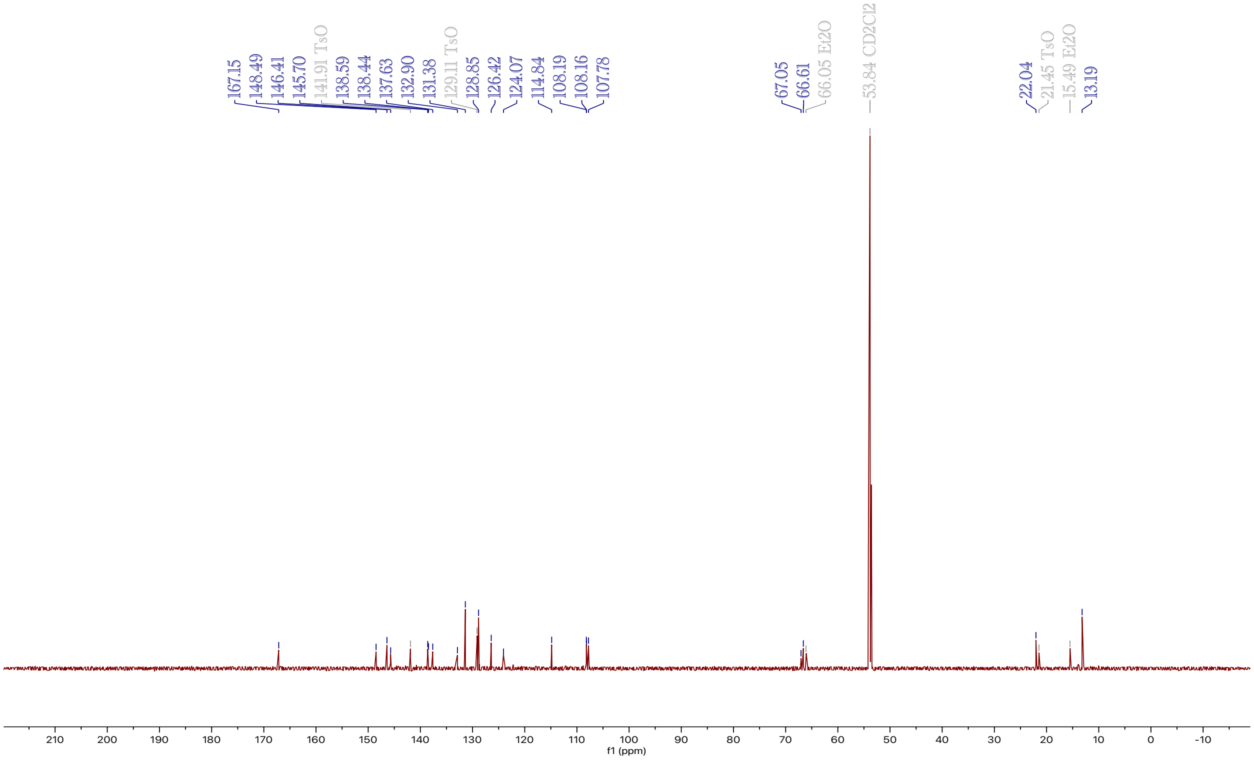

Figure S3: ^{1}H NMR, 800 MHz, CD_3CN, 25 °C, **6D**

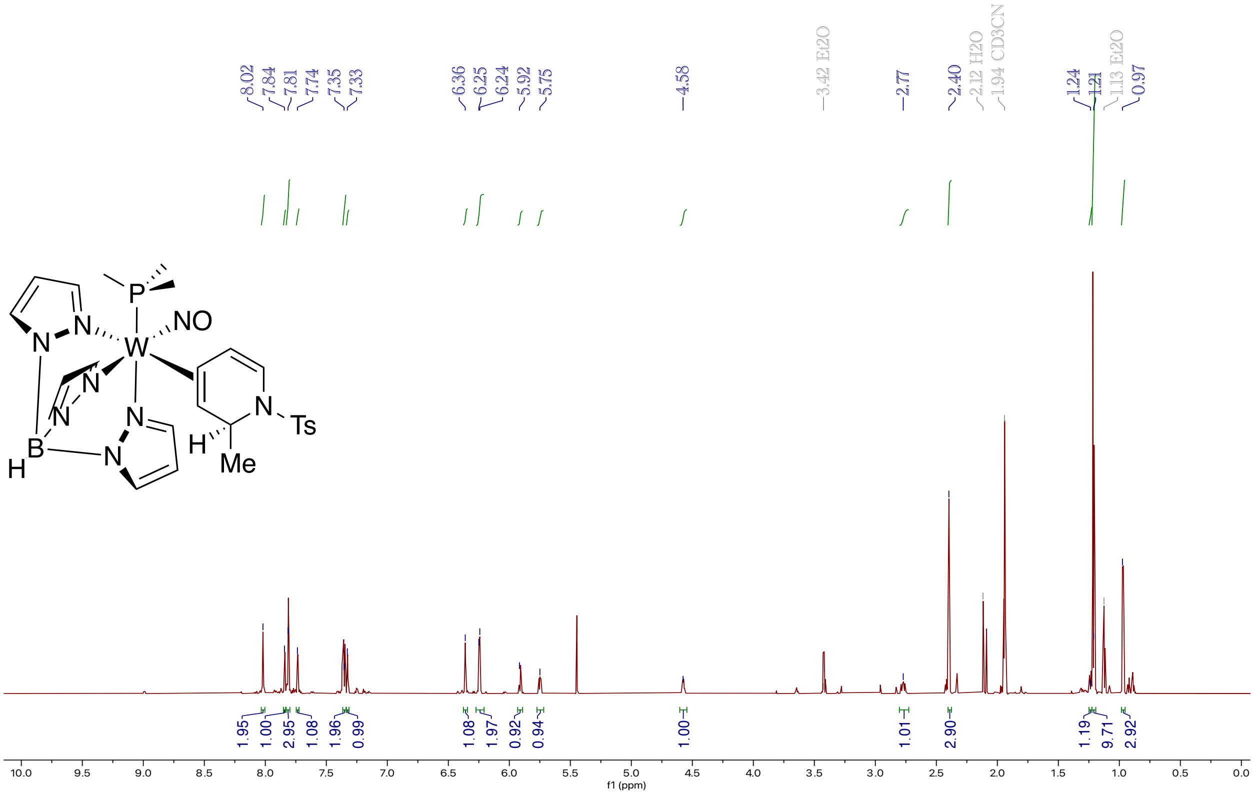

Figure S4: ^{13}C NMR, 200 MHz, CD_3CN, 25 °C, **6D**

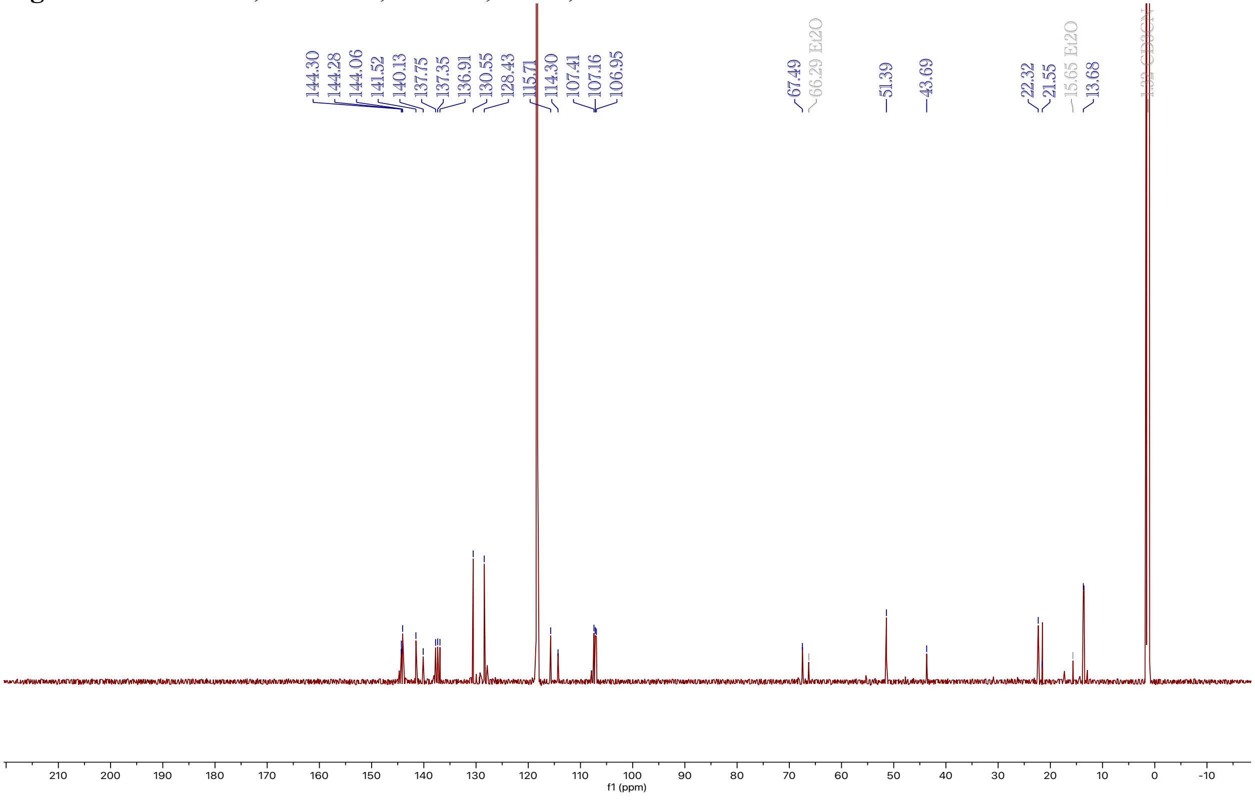

Figure S5: ^{1}H NMR, 800 MHz, CD_2Cl_2, 25 °C, **7D**

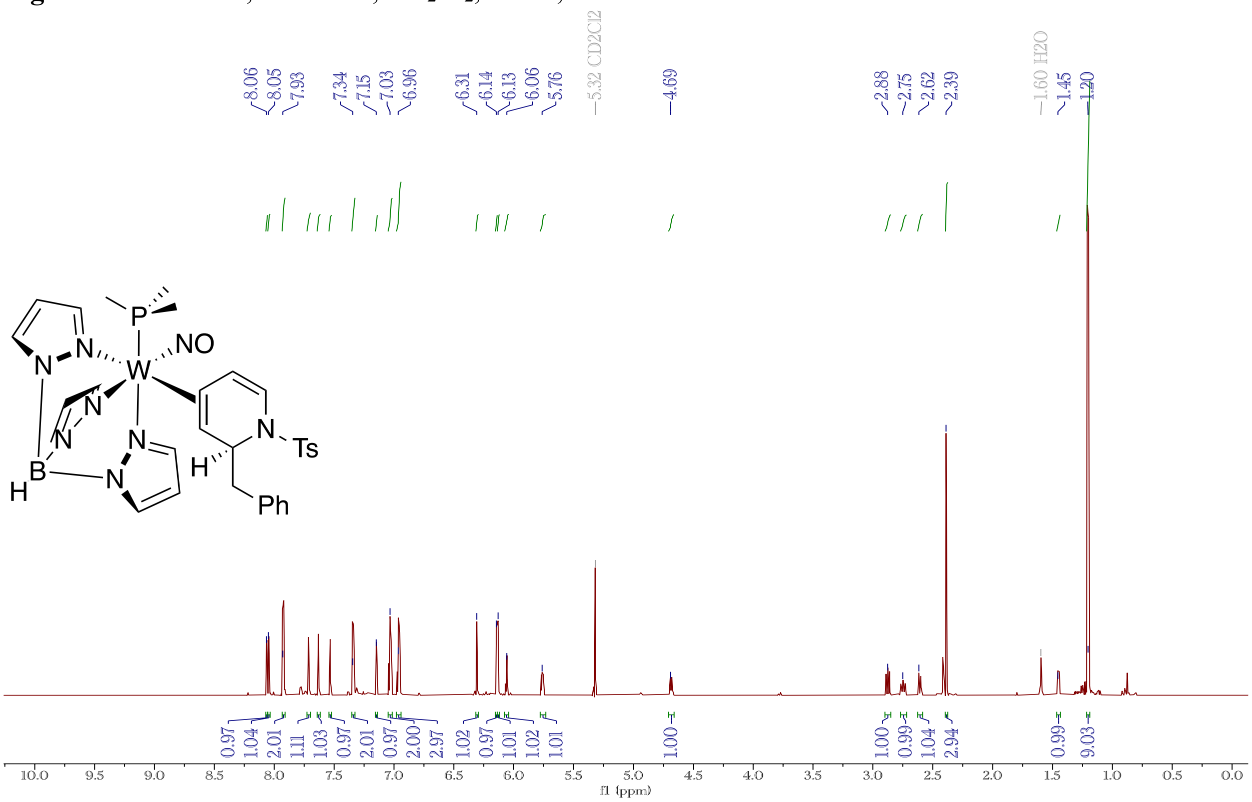

Figure S6: ^{13}C NMR, 200 MHz, CD_2Cl_2, 25 °C, **7D**

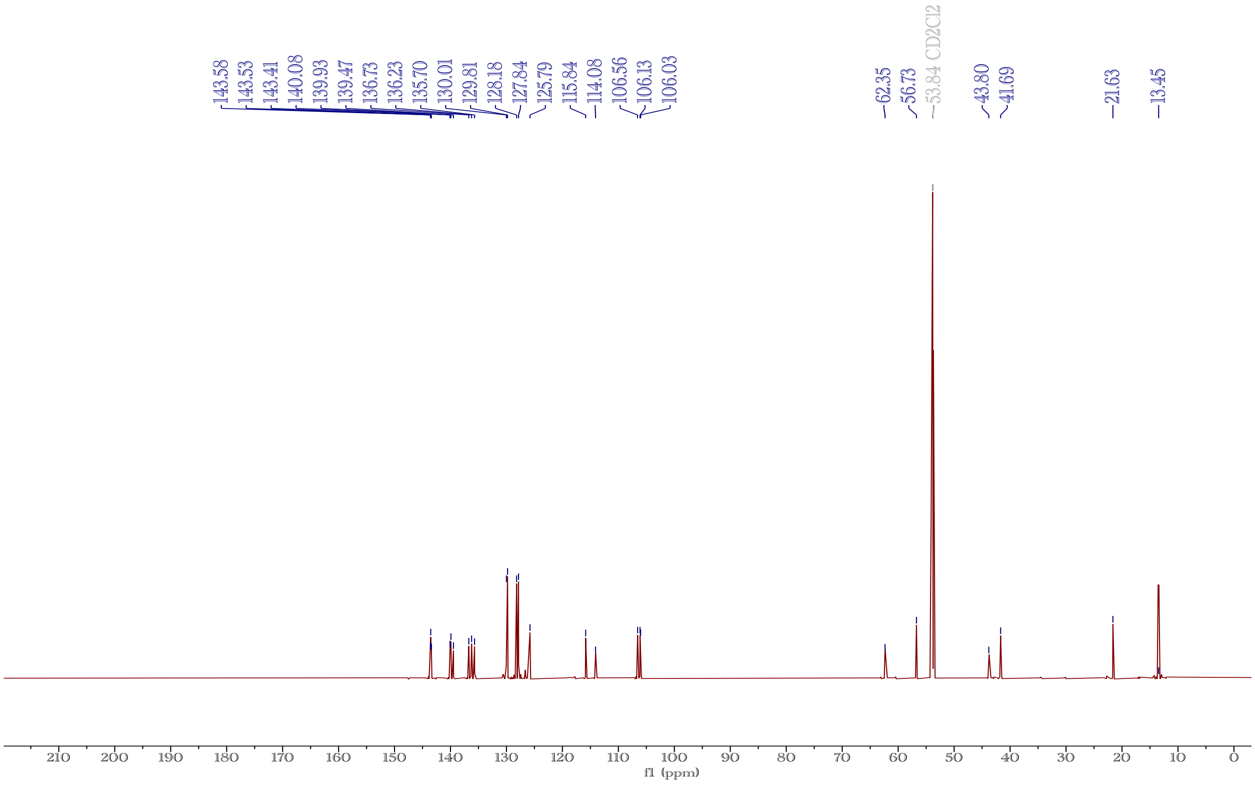

Figure S7: ^{1}H NMR, 800 MHz, CD_2Cl_2, 25 °C, **8D**

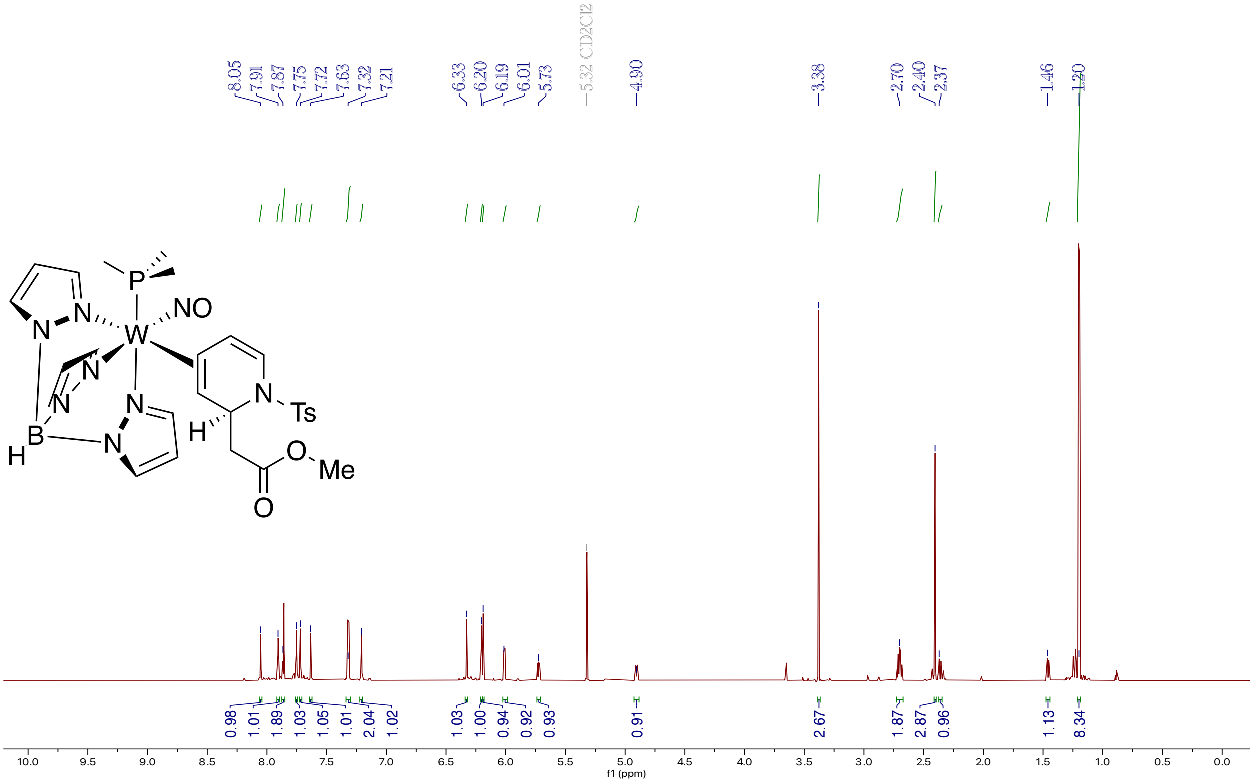

Figure S8: ^{13}C NMR, 200 MHz, CD_2Cl_2, 25 °C, **8D**

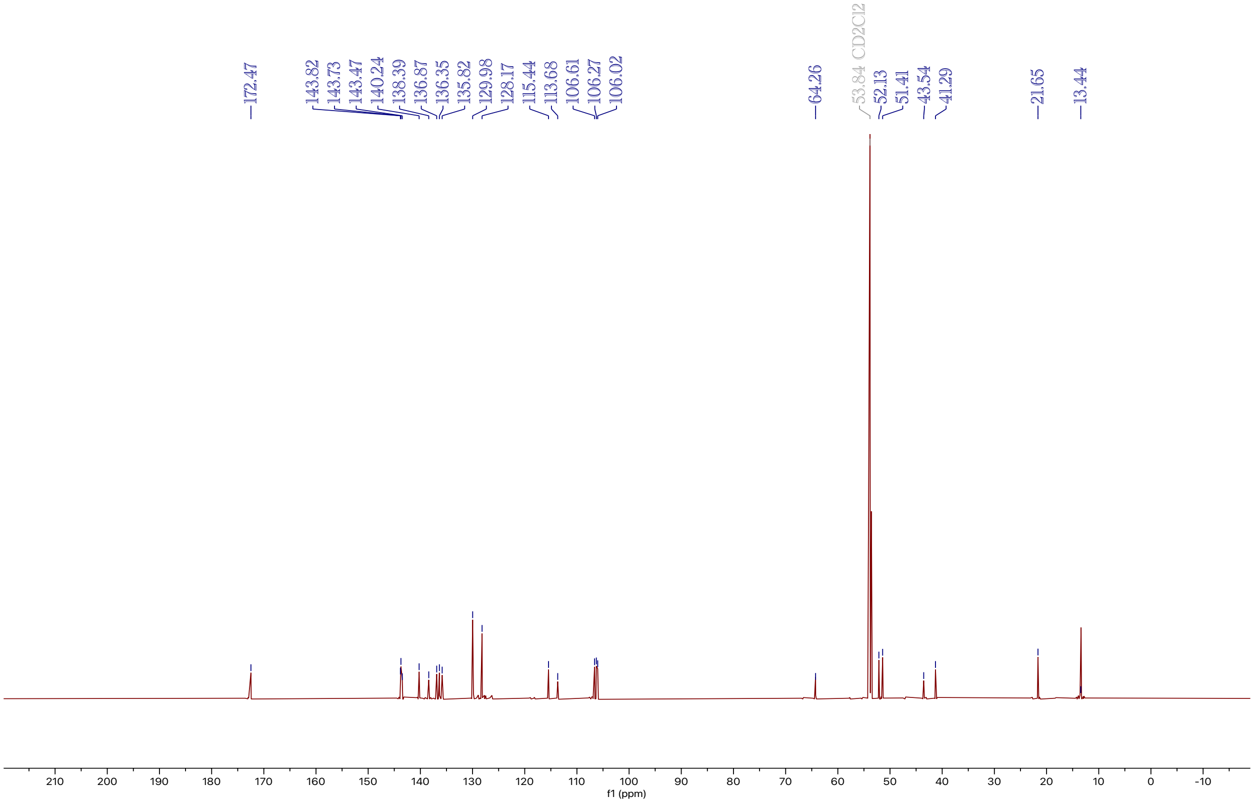

Figure S9: ^{1}H NMR, 800 MHz, CD_2Cl_2, 25 °C, **9D**

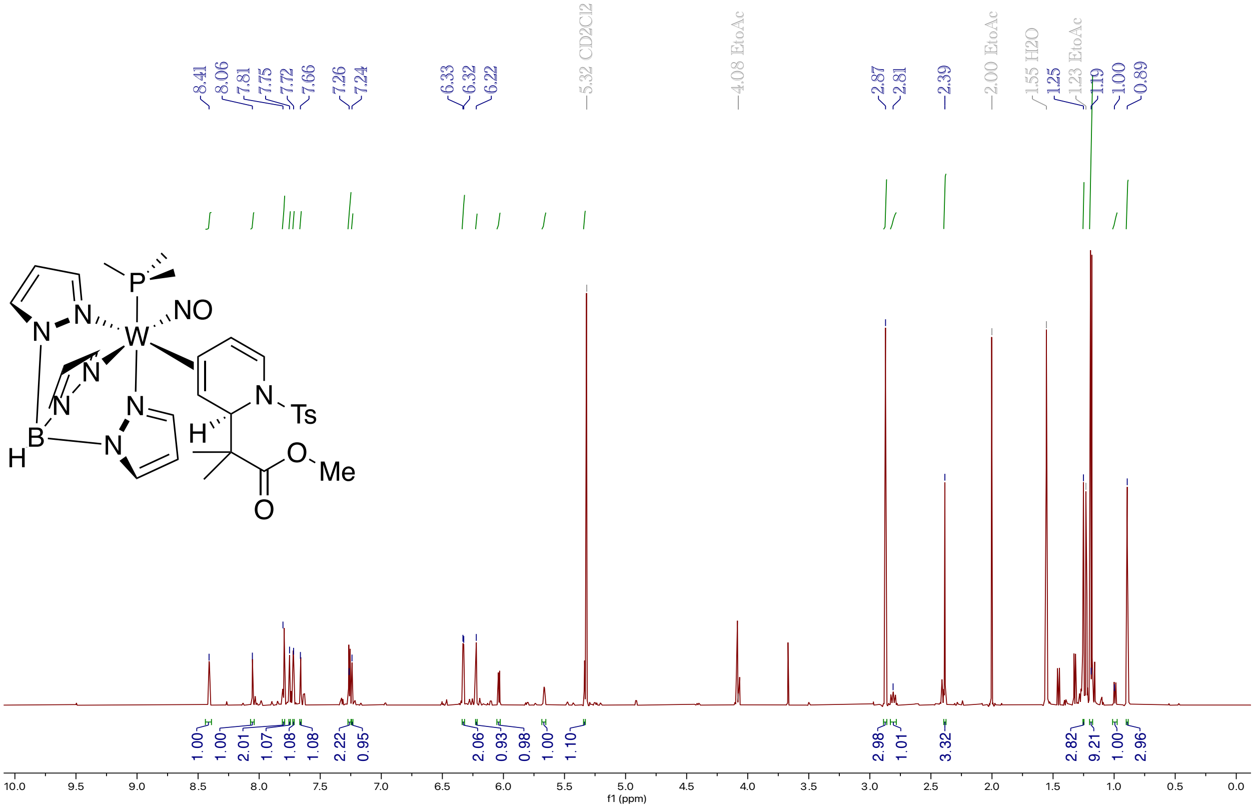

Figure S10: ^{13}C NMR, 200 MHz, CD_2Cl_2, 25 °C, **9D**

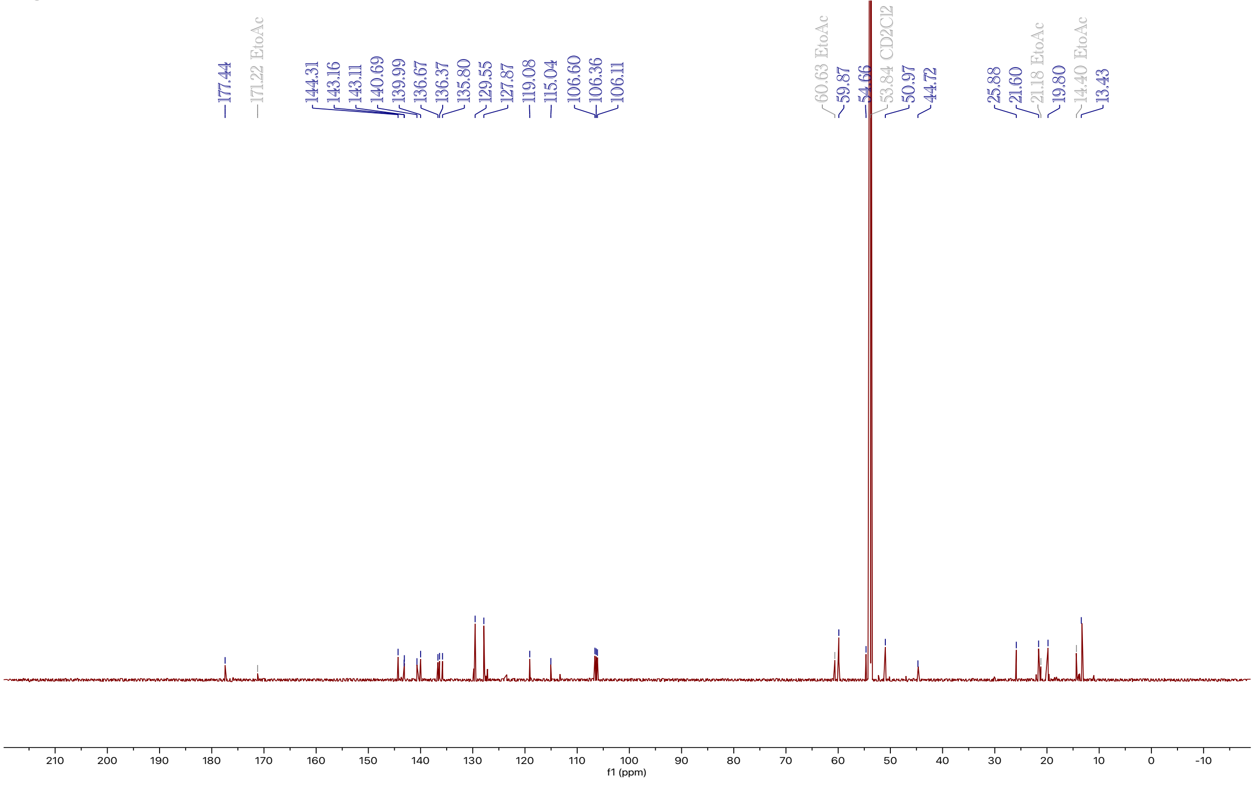

Figure S11: NOESY NMR 800 MHz, CD_2Cl_2, 25 °C, **9D**

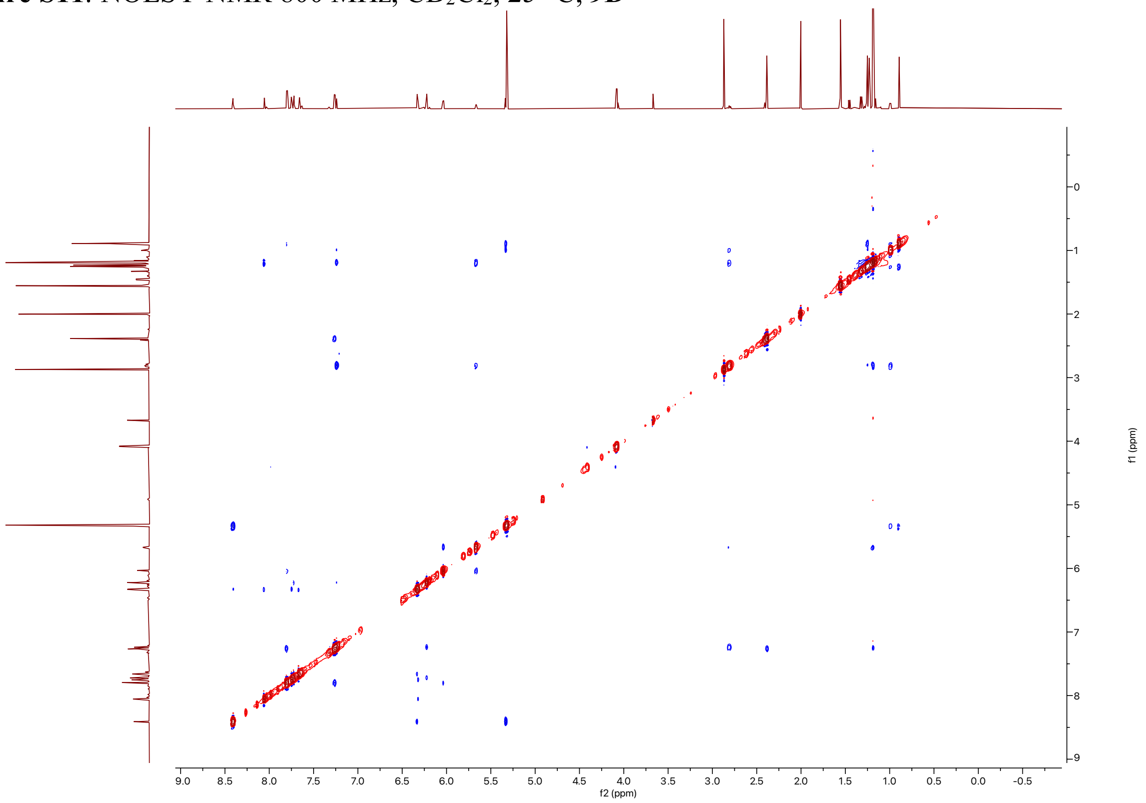

Figure S12: COSY NMR 800 MHz, CD_2Cl_2, 25 °C, **9D**

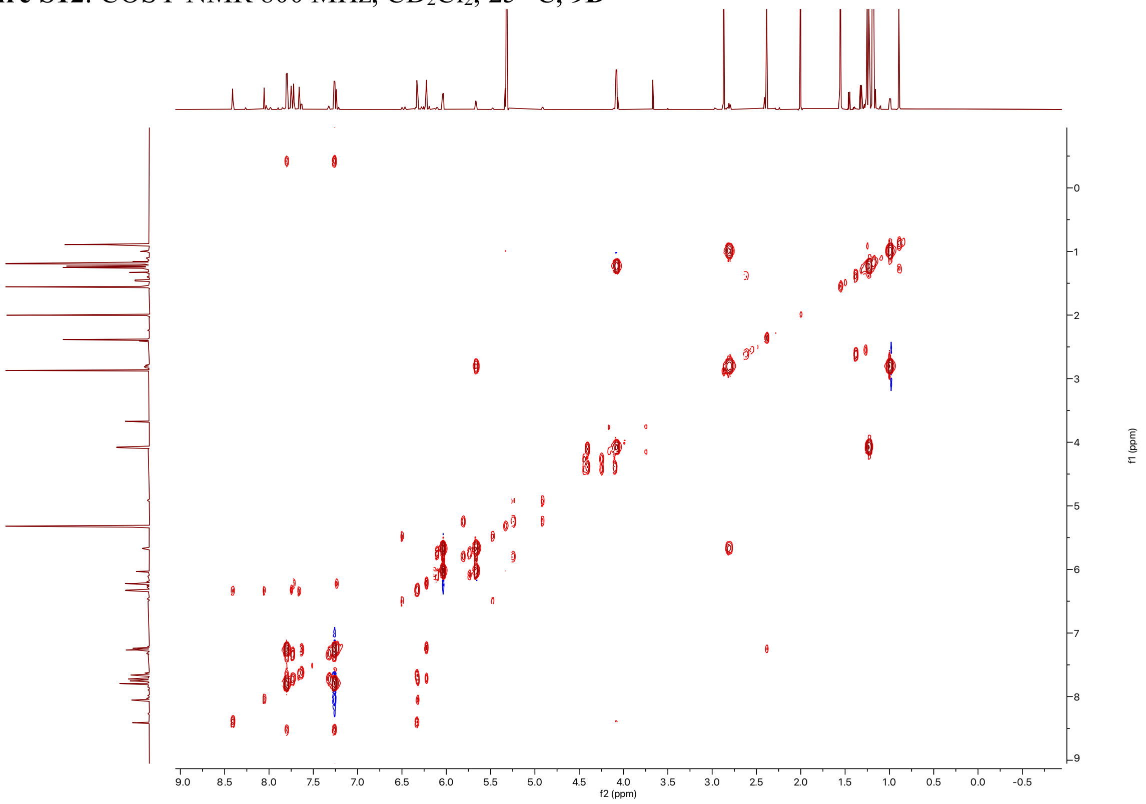

Figure S13: HSQC NMR 800 MHz, CD_2Cl_2, 25 °C, **9D**

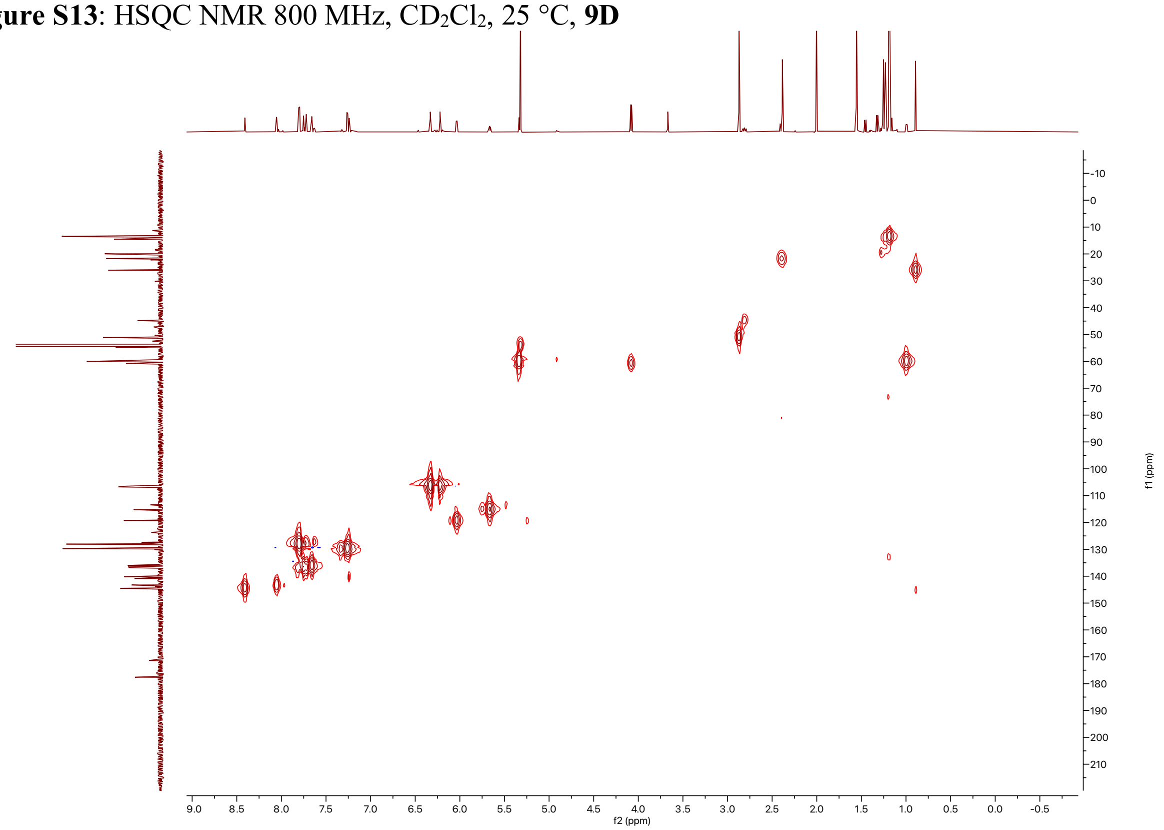

Figure S14: HMBC NMR 800 MHz, CD_2Cl_2, 25 °C, **9D**

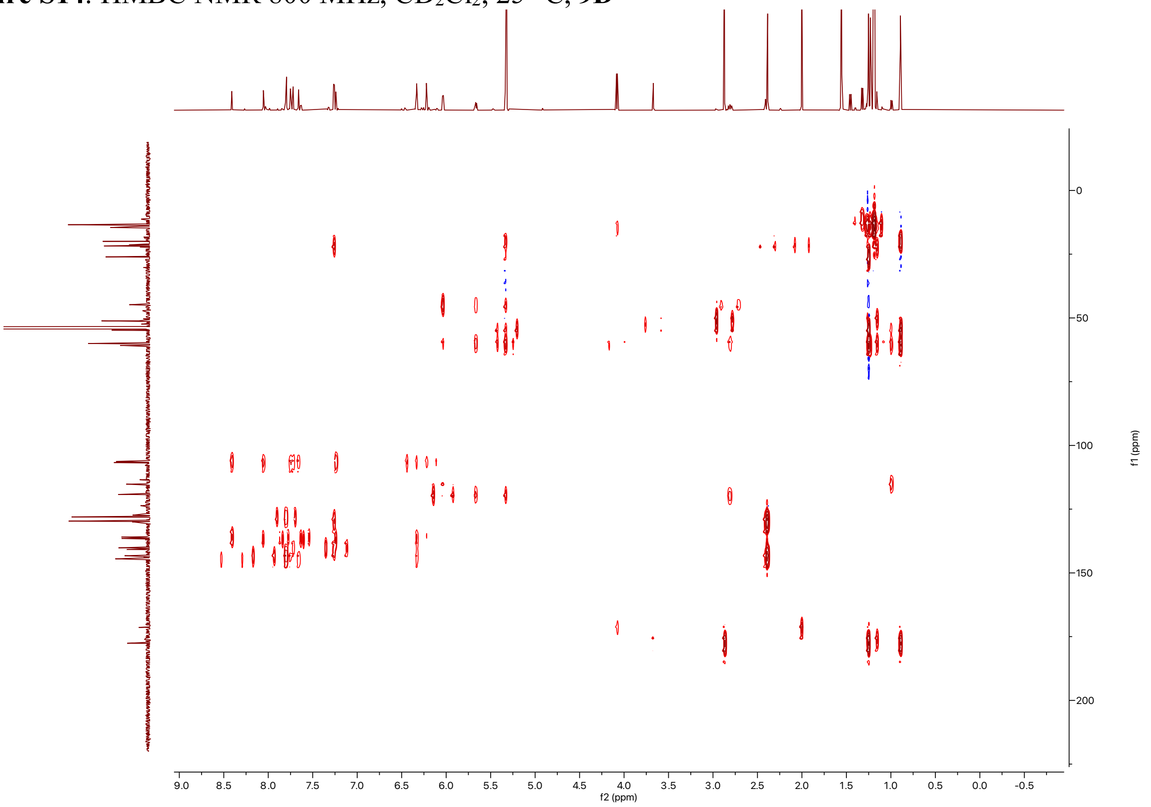

Figure S15: ^{1}H NMR, 800 MHz, CD_2Cl_2, 25 °C, **10D**

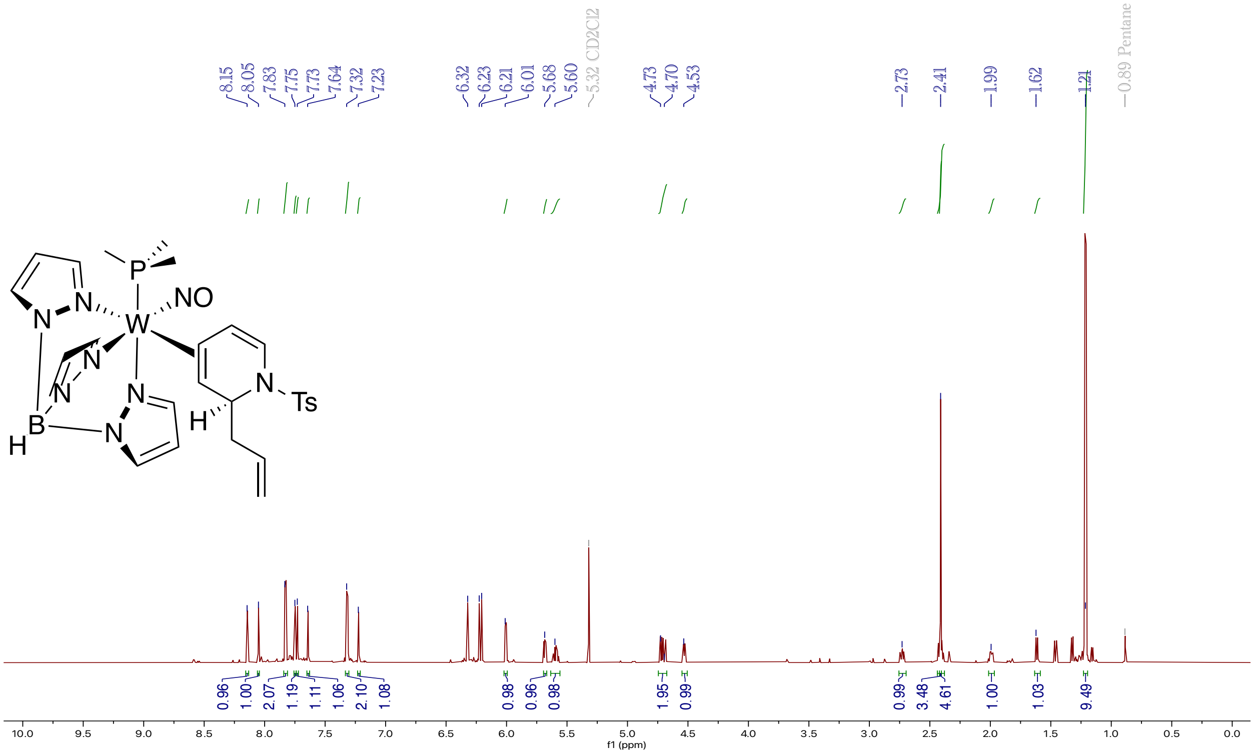

Figure S16: ^{13}C NMR, 200 MHz, CD_2Cl_2, 25 °C, **10D**

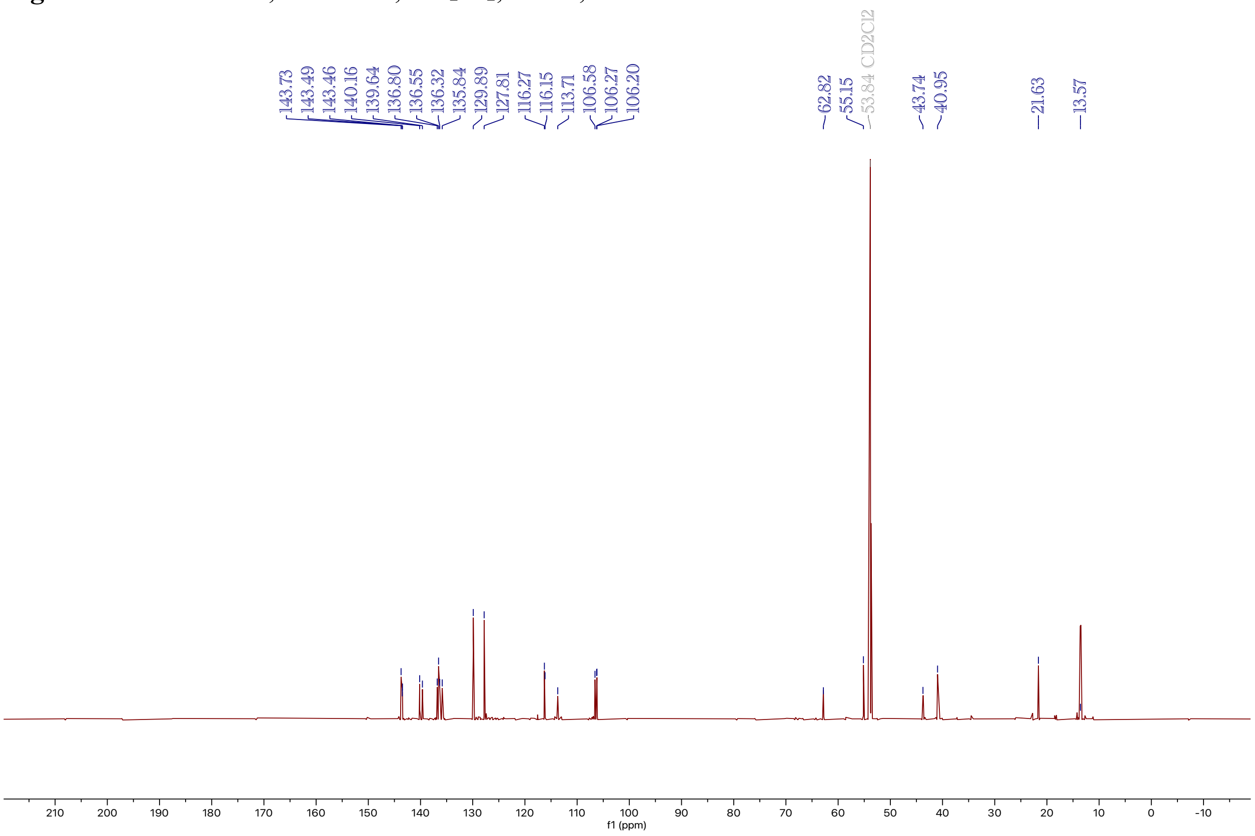

Figure S17: ^{1}H NMR, 800 MHz, CD_2Cl_2, 25 °C, **11D**

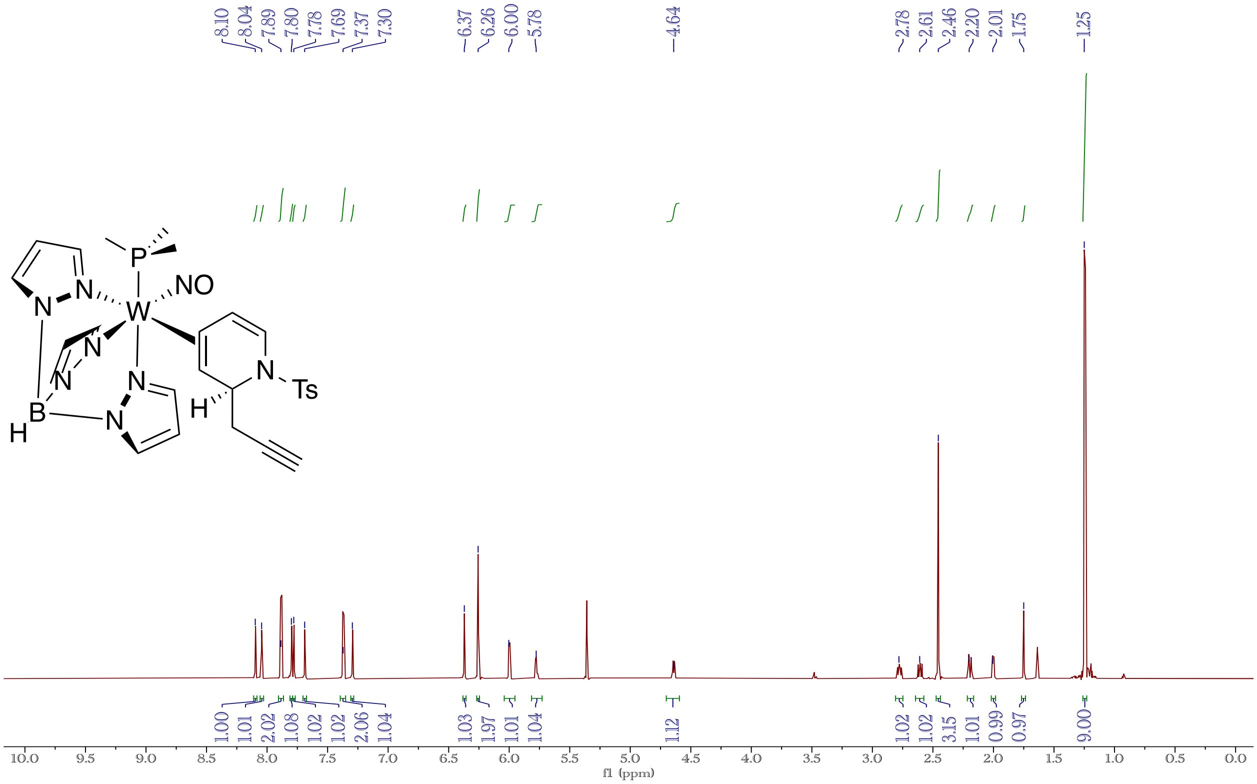

Figure S18: ^{13}C NMR, 200 MHz, CD_2Cl_2, 25 °C, **11D**

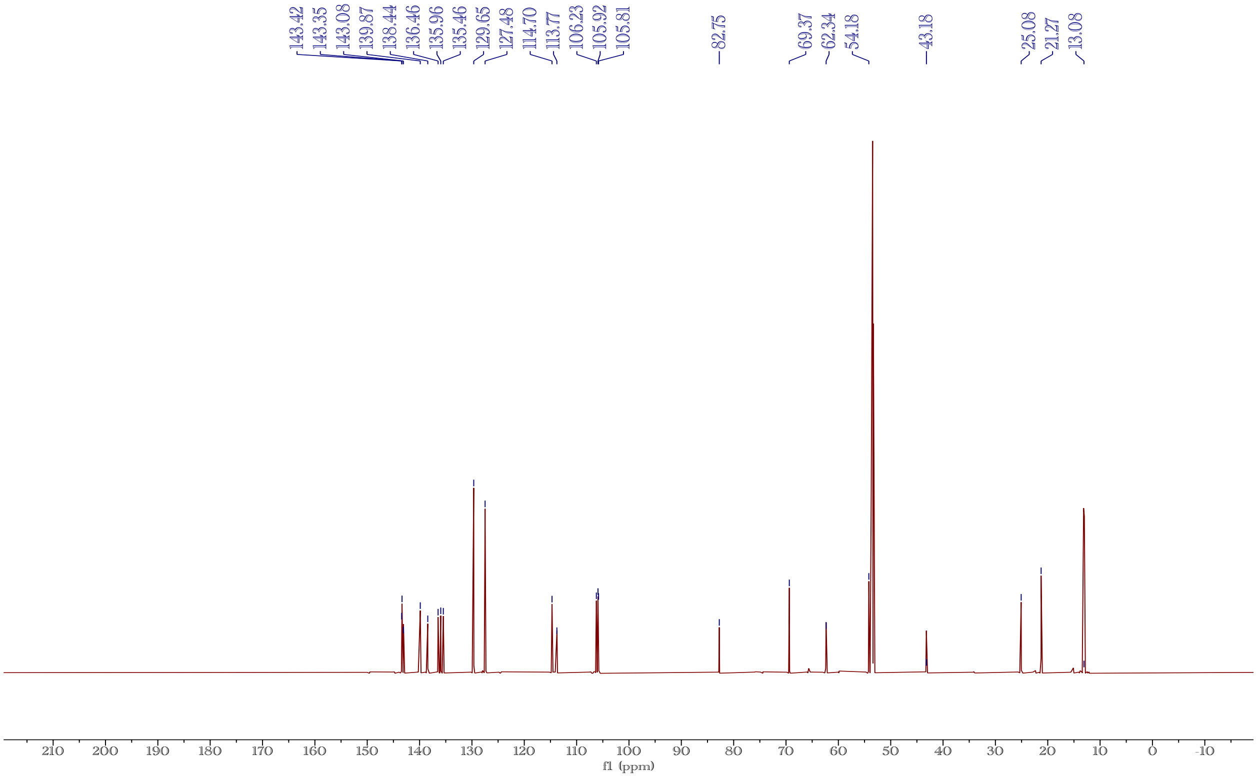

Figure S19: ^{1}H NMR, 800 MHz, CD_2Cl_2, 25 °C, **12D**

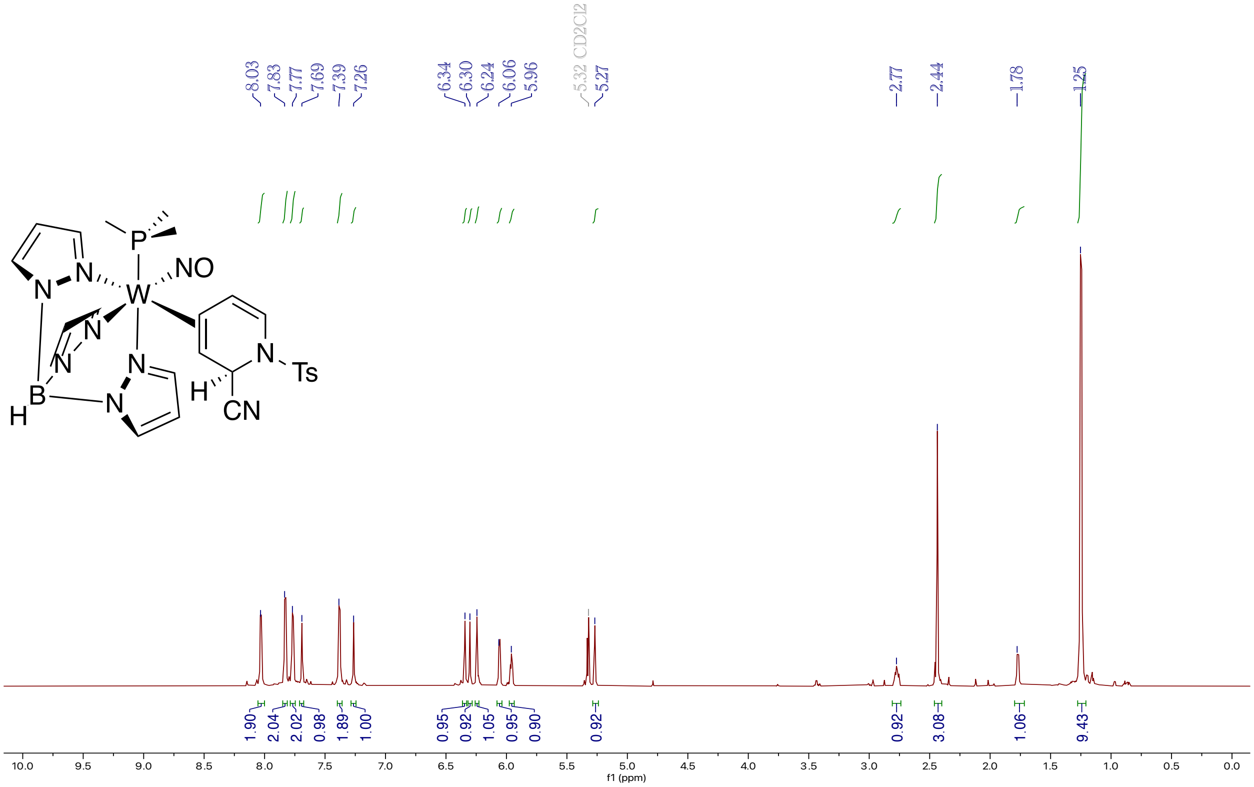

Figure S20: ^{13}C NMR, 200 MHz, CD_2Cl_2, 25 °C, **12D**

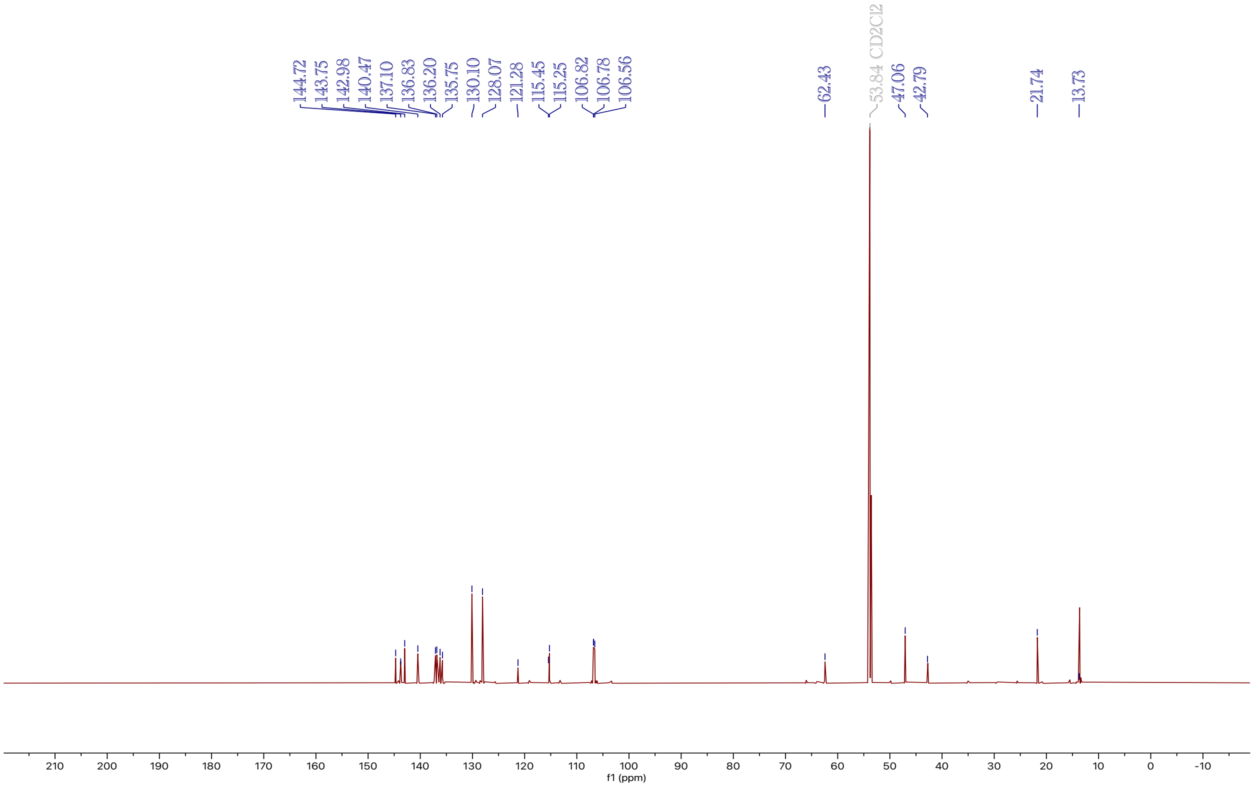

Figure S21: ^{1}H NMR, 800 MHz, CD_2Cl_2, 25 °C, **13D**

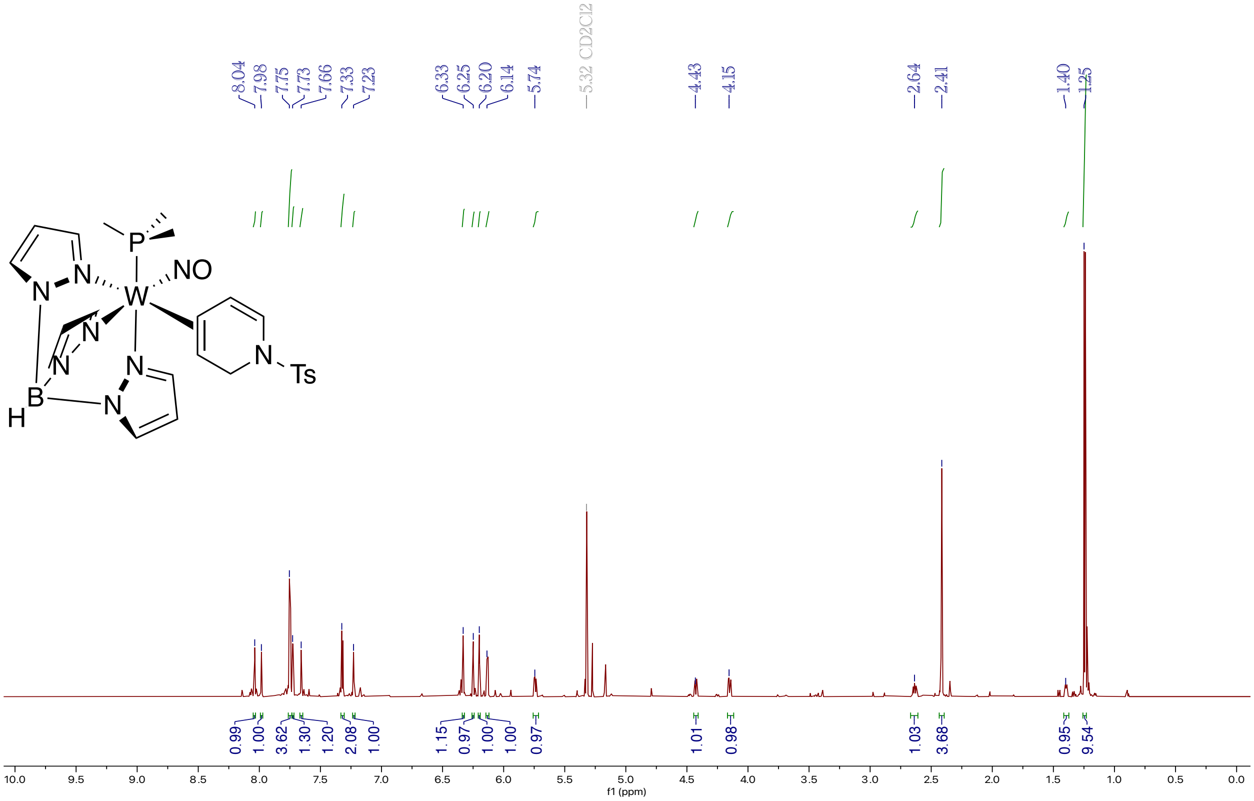

Figure S22: ^{13}C NMR, 200 MHz, CD_2Cl_2, 25 °C, **13D**

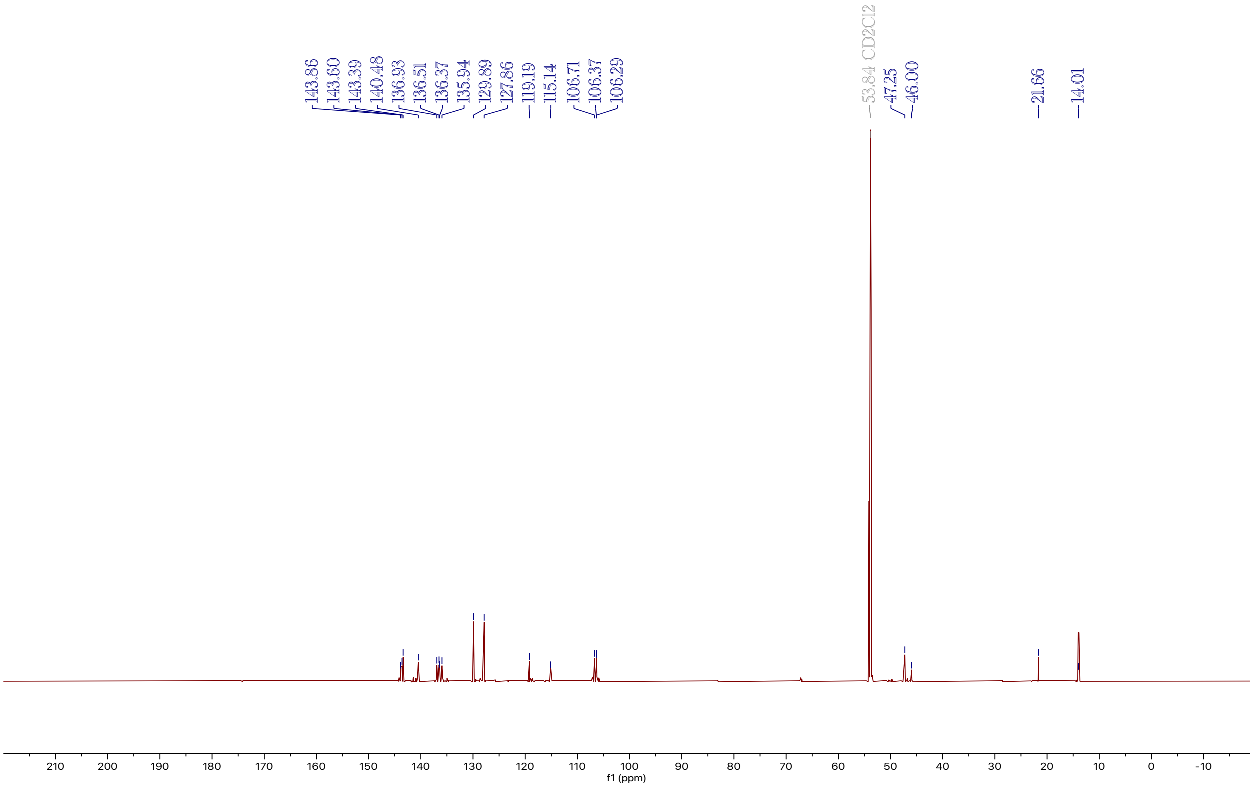

Figure S23: ^{1}H NMR, 800 MHz, CD_2Cl_2, 25 °C, **15D**

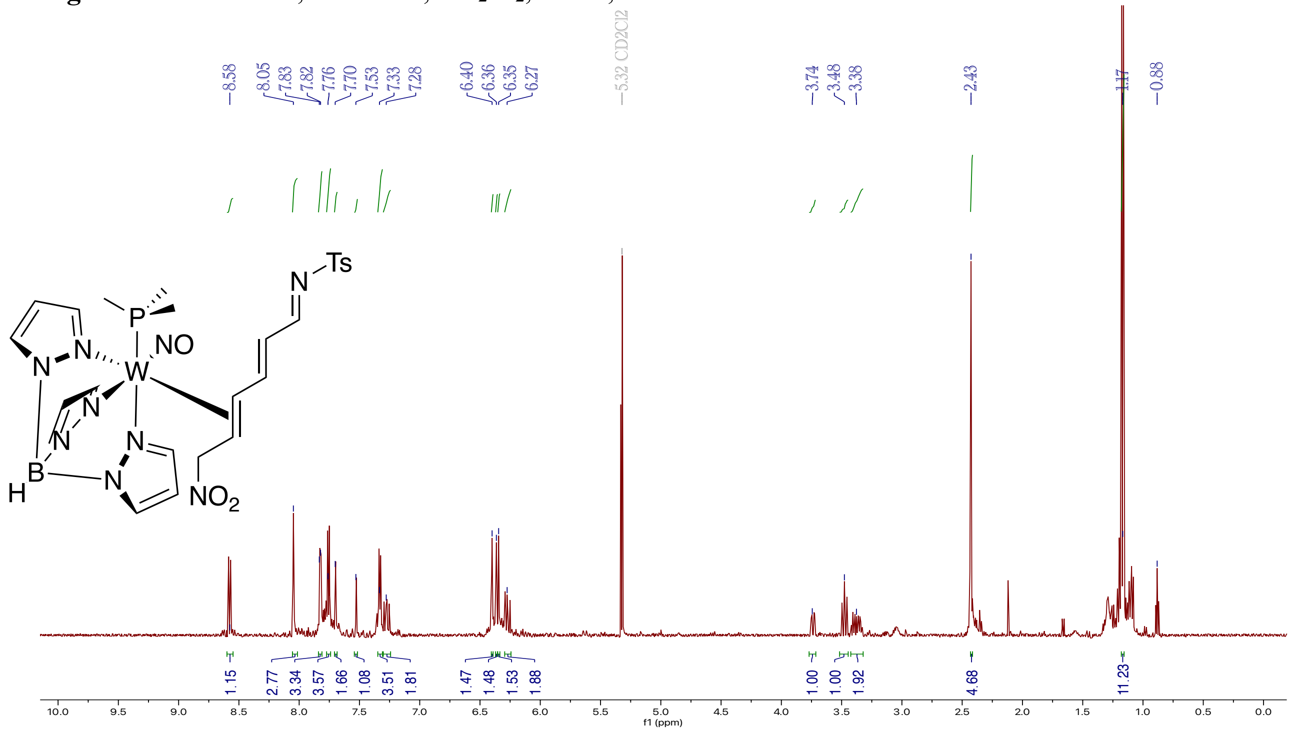

Figure S24: ^{13}C NMR, 200 MHz, CD_2Cl_2, 25 °C, **15D**

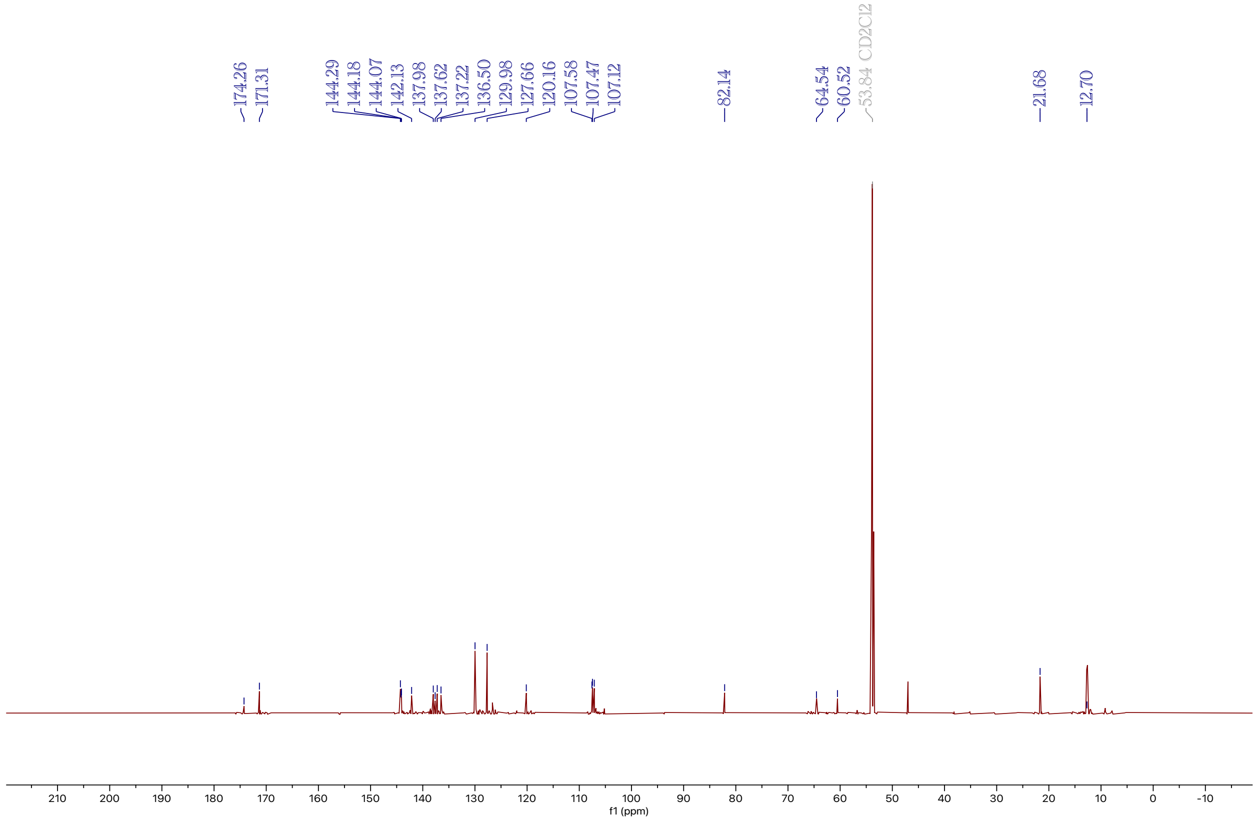

Figure S25: ^{1}H NMR, 800 MHz, $CDCl_3$, 25 °C, **16D**

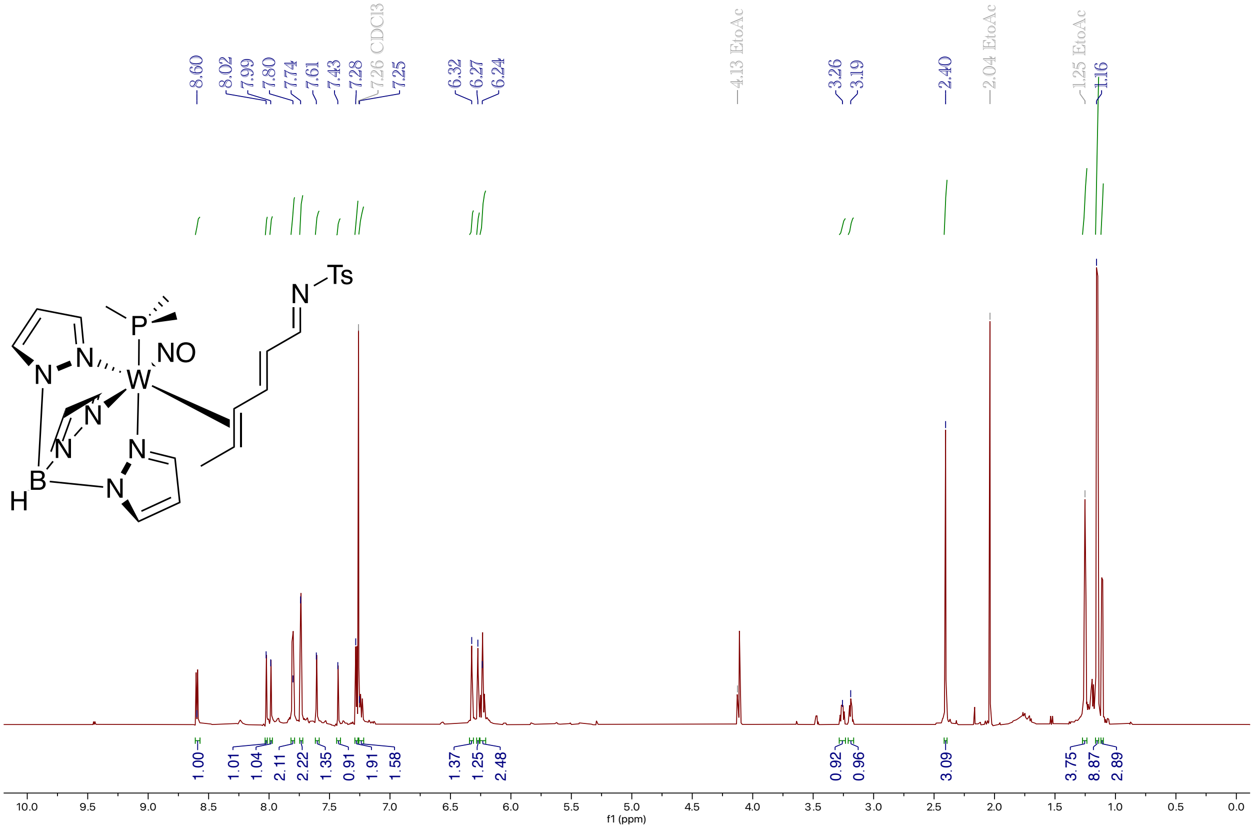

Figure S26: ^{13}C NMR, 200 MHz, $CDCl_3$, 25 °C, **16D**

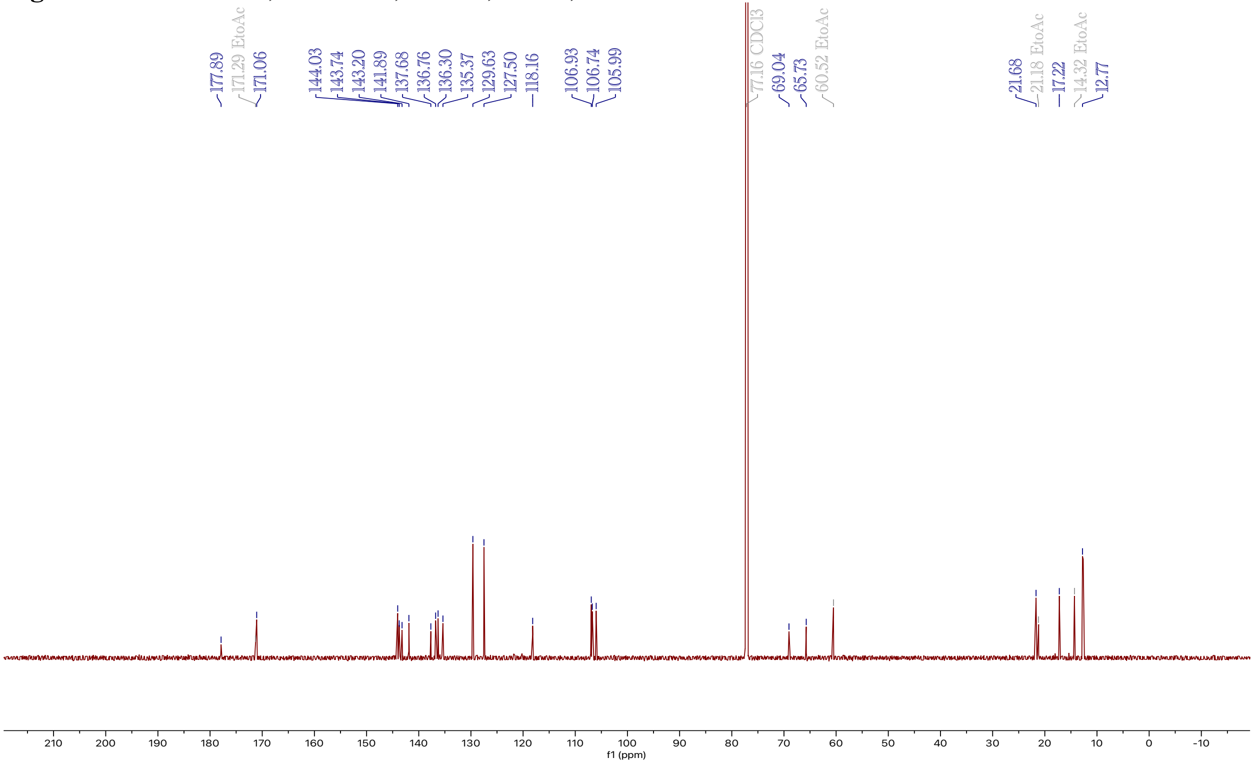

Figure S27: ^{1}H NMR, 800 MHz, CD_2Cl_2, 25 °C, **17D**

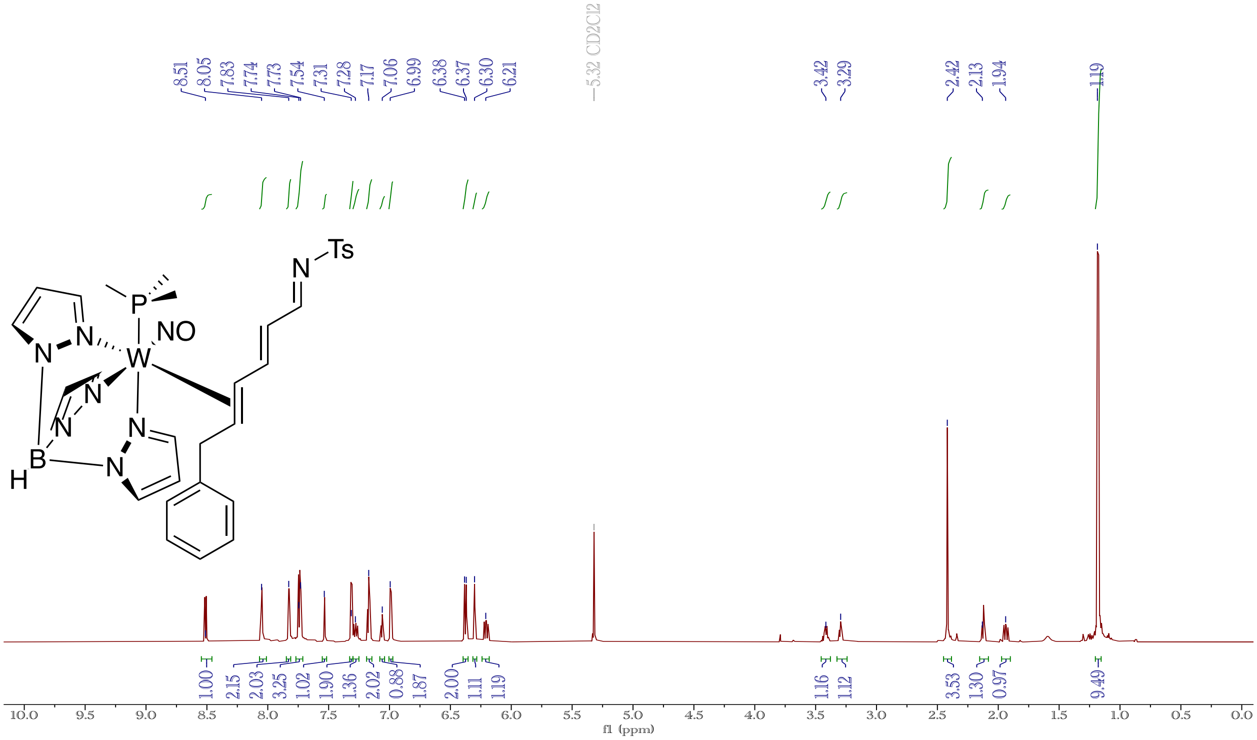

Figure S28: ^{13}C NMR, 200 MHz, CD_2Cl_2, 25 °C, **17D**

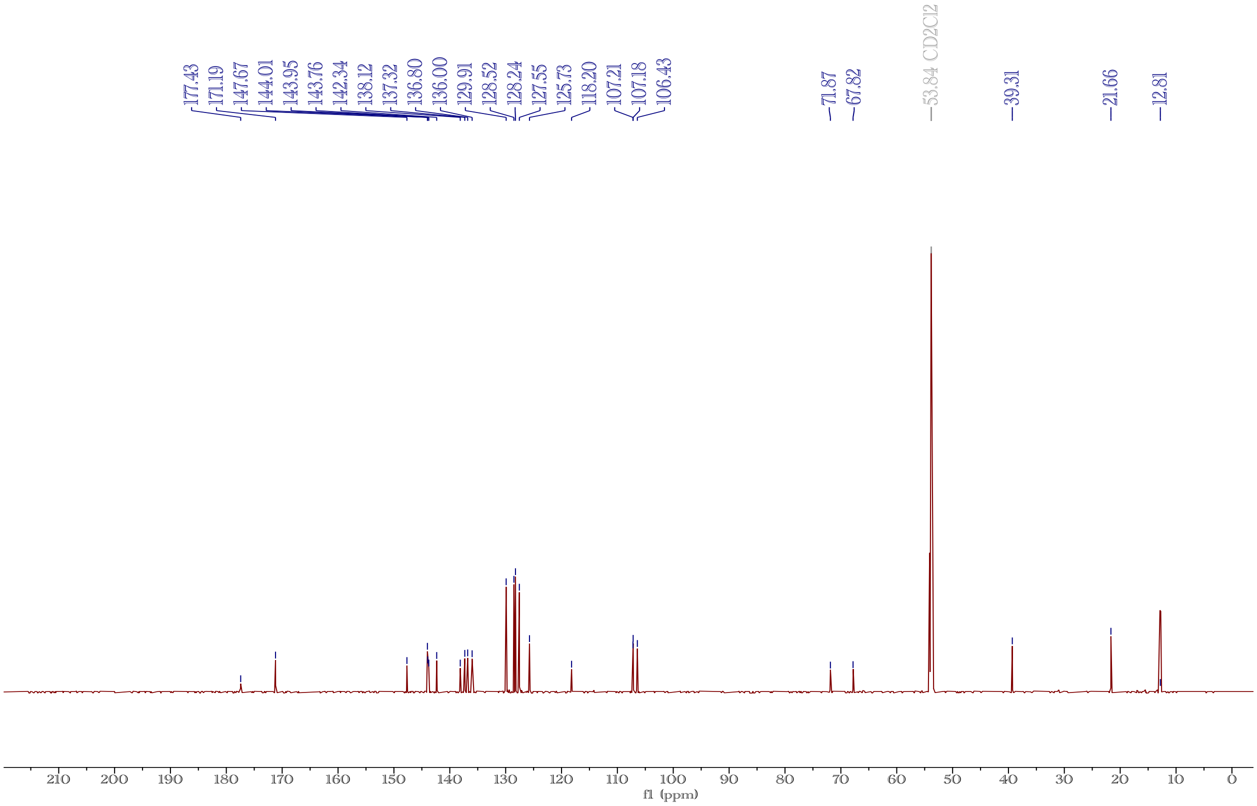

Figure S29: ^{1}H NMR, 800 MHz, CD_3CN, 25 °C, **18D**

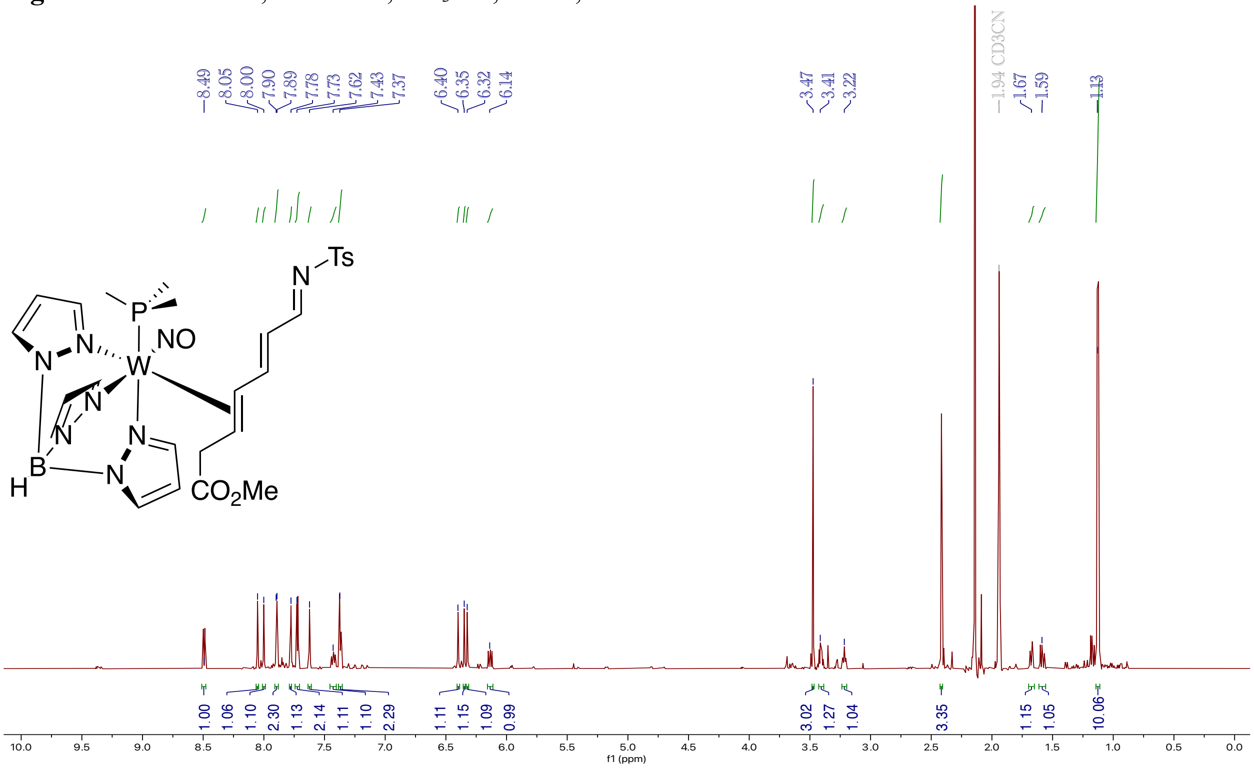

Figure S30: ^{13}C NMR, 200 MHz, CD_3CN, 25 °C, **18D**

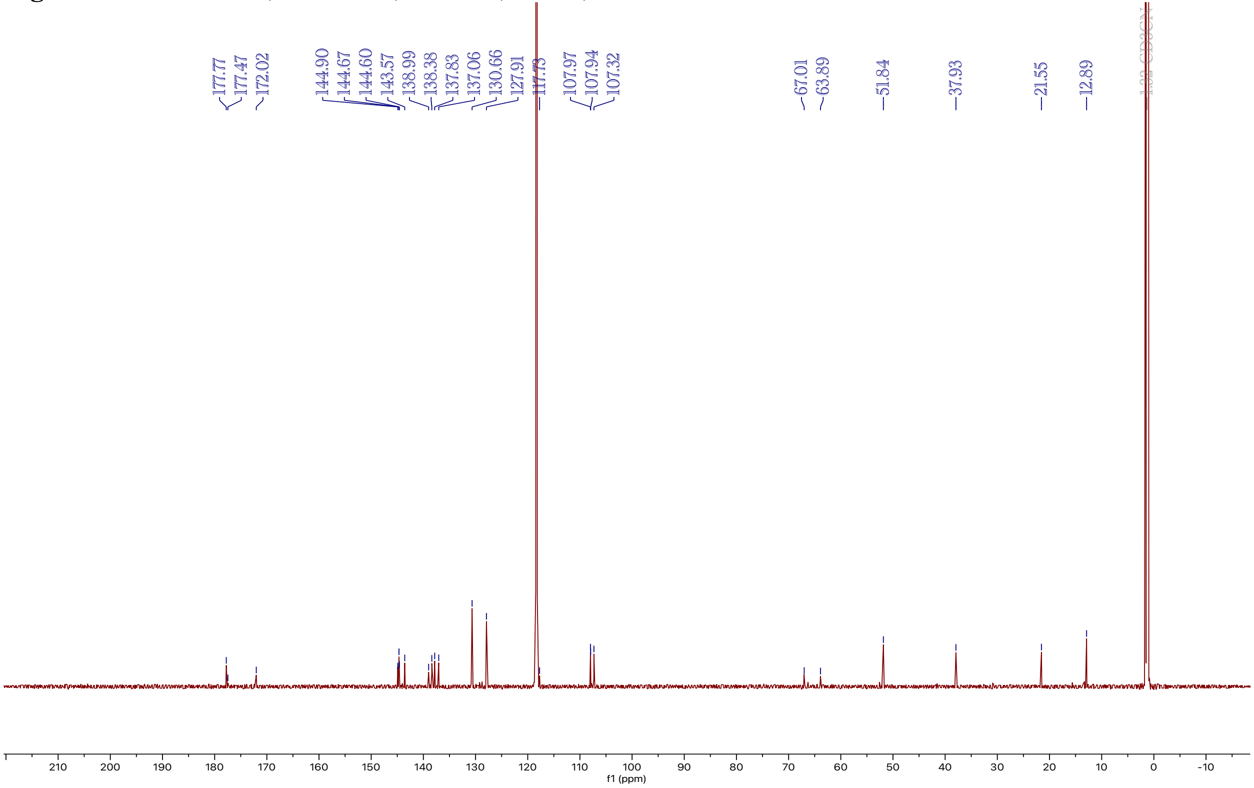

Figure S31: ^{1}H NMR, 800 MHz, CD_2Cl_2, 25 °C, **19D**

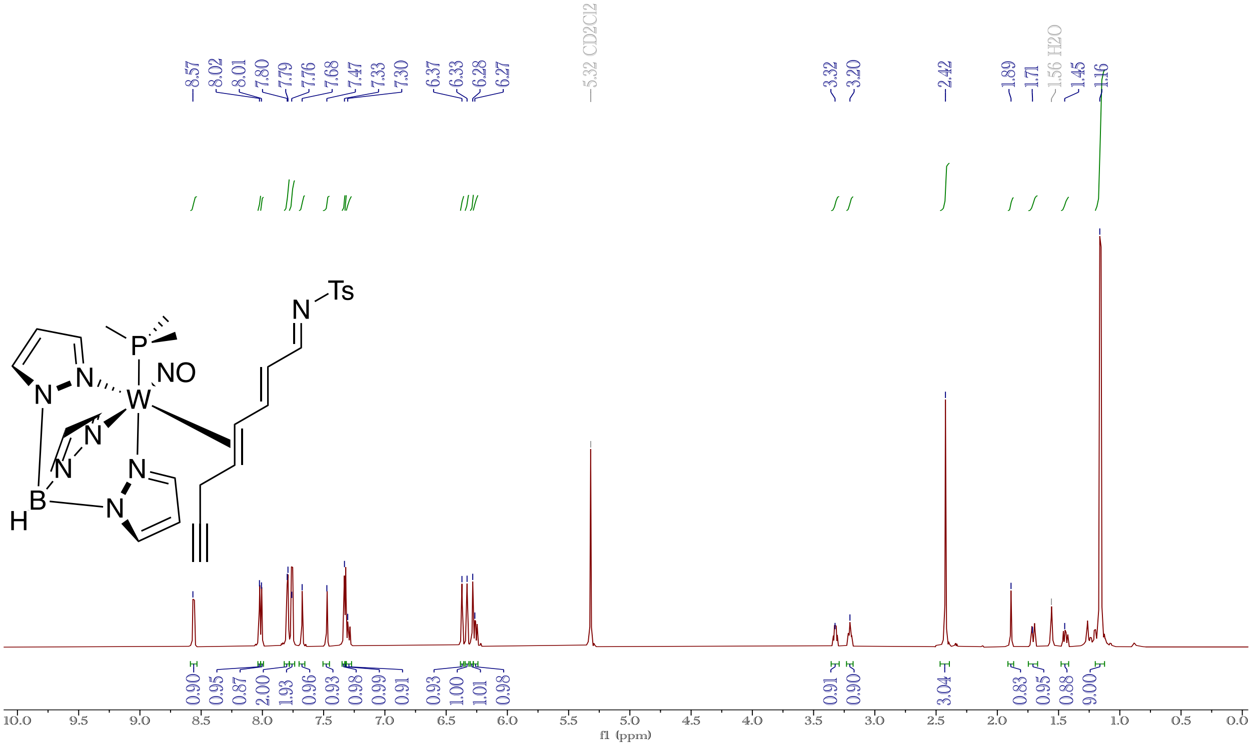

Figure S32: ^{13}C NMR, 200 MHz, CD_2Cl_2, 25 °C, **19D**

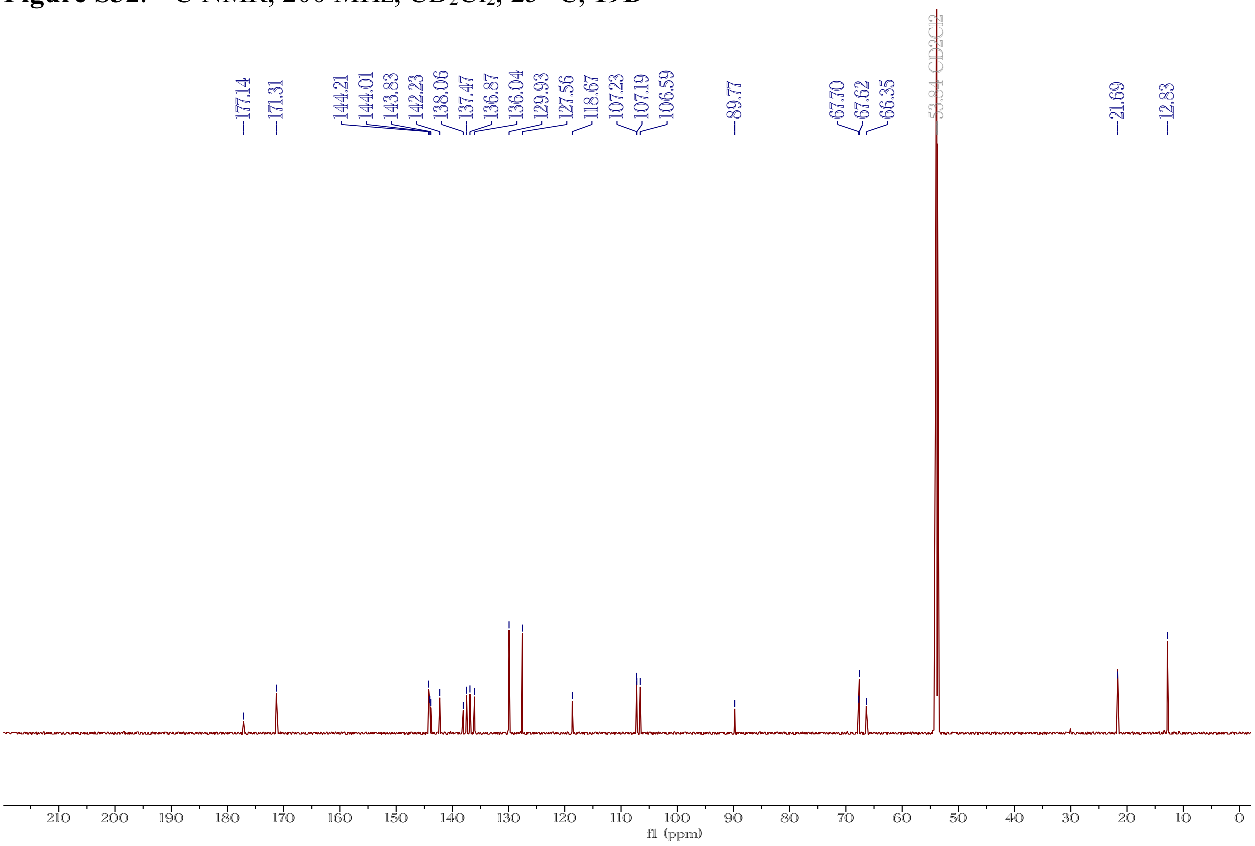

SC-XRD DATA:

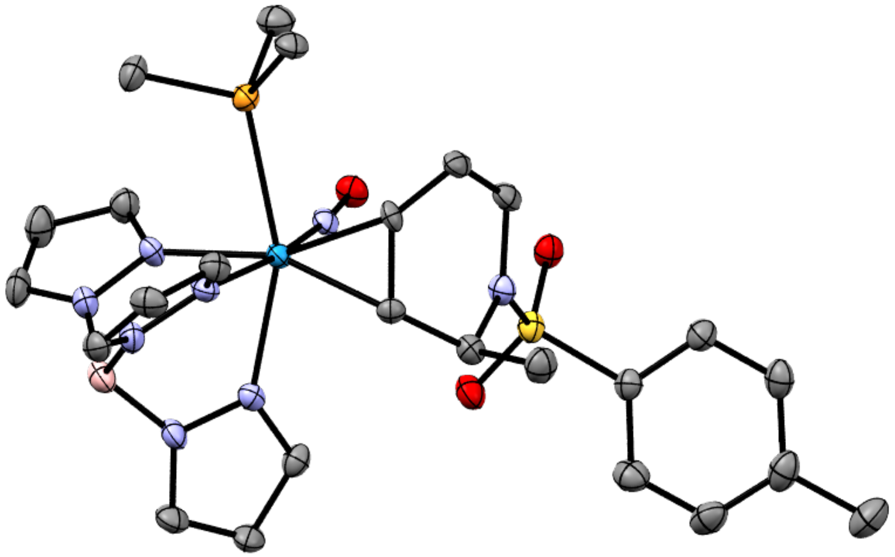

Figure S33: ORTEP/ellipsoid diagram of **6D**.

Table S1: SC-XRD data for **6D**.

CCDC<br>2323731	Chemical Formula<br>$C_{25}H_{34}BN_8O_3PSW$	FW (g/mol)<br>752.29
T (K)<br>100(2)	λ (Å)<br>0.71073	Crystal size (mm)<br>0.025 x 0.032 x 0.615
Crystal habit<br>Colorless needle	Crystal system<br>monoclinic	Space group<br>$P\ 2_1/c$
a (Å)<br>13.9628(4)	b (Å)<br>12.4970(4)	c (Å)<br>16.9468(5)
α (°)<br>90	β (°)<br>91.0580(10)	γ (°)<br>90
V ($Å^3$)<br>2956.60(15)	Z<br>4	ρ_{calc} (g/cm^3)<br>1.690
μ (mm^{-1})<br>4.074	F(000)<br>1496	θ range (°)<br>2.40 to 27.50
Index ranges<br>$-18 \le h \le 18$<br>$-16 \le k \le 16$<br>$-22 \le l \le 18$	Data/restraints/parameters<br>6780 / 0 / 369	Goodness-of-fit on F^2<br>0.999
R_1 [I>2σ(I)]<br>0.0374	wR_2 [all data]<br>0.0863	

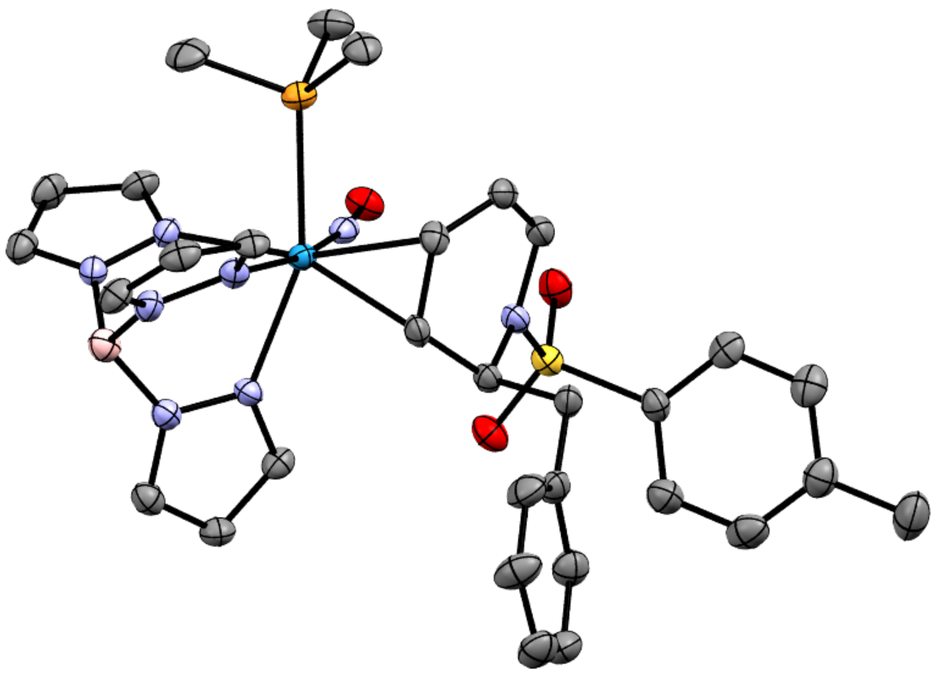

Figure S34: ORTEP/ellipsoid diagram of **7D**.

Table S2: SC-XRD data for **7D**.

CCDC	Chemical Formula	FW (g/mol)
2323732	$C_{31}H_{38}BN_8O_3PSW$	828.38
T (K)	λ (Å)	Crystal size (mm)
100(2)	0.71073	0.018 x 0.027 x 0.086
Crystal habit	Crystal system	Space group
Yellow habit	Monoclinic	$P\ 2_{1/}n$
a (Å)	b (Å)	c (Å)
18.1833(13)	8.3445(5)	22.3133(15)
α (°)	β (°)	γ (°)
90	99.876(2)	90
V (Å^3)	Z	ρ_{calc} (g/cm^3)
3335.4(4)	4	1.650
μ (mm^{-1})	F(000)	θ range (°)
3.620	1656	2.27 to 28.30
Index ranges	Data/restraints/parameters	Goodness-of-fit on F^2
$-24 \leq h \leq 17$<br>$-10 \leq k \leq 11$<br>$-29 \leq l \leq 29$	8273 / 0 / 425	1.010
R_1 [I>2σ(I)]	wR_2 [all data]	
0.0434	0.1000	

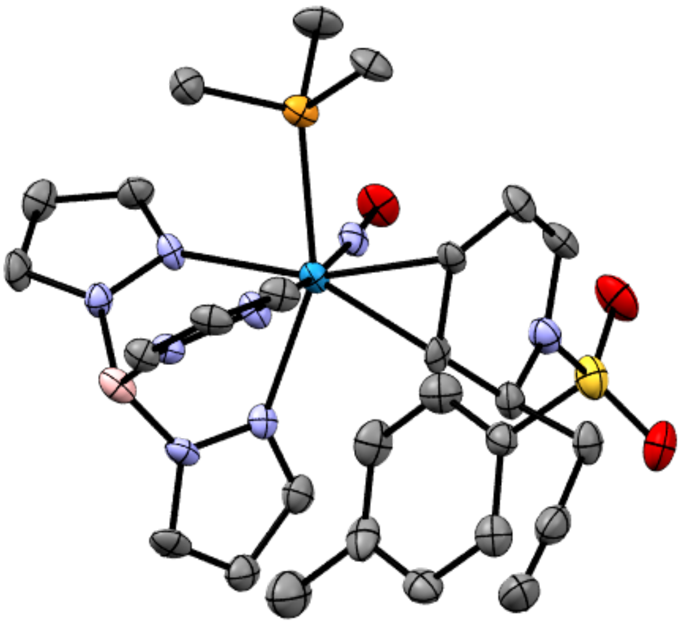

Figure S35: ORTEP/ellipsoid diagram of **11D**.

Table S3: SC-XRD data for **11D**.

CCDC	Chemical Formula	FW (g/mol)
2323733	$C_{27}H_{34}BN_8O_3PSW$	776.31
T (K)	λ (Å)	Crystal size (mm)
100(2)	0.71073	0.054 x 0.170 x 0.245
Crystal habit	Crystal system	Space group
Colorless plate	Orthorhombic	P $2_12_12_1$
a (Å)	b (Å)	c (Å)
10.0236(7)	12.8806(8)	23.7119(16)
α (°)	β (°)	γ (°)
90	90	90
V (Å^3)	Z	ρ_{calc} (g/cm^3)
3061.4(4)	4	1.684
μ (mm^{-1})	F(000)	θ range (°)
3.937	1544	1.80 to 27.16
Index ranges	Data/restraints/parameters	Goodness-of-fit on F^2
$-12 \leq h \leq 12$<br>$-16 \leq k \leq 16$<br>$-30 \leq l \leq 30$	6759 / 0 / 396	1.060
R_1 [I>2σ(I)]	wR_2 [all data]	
0.0412	0.0836	

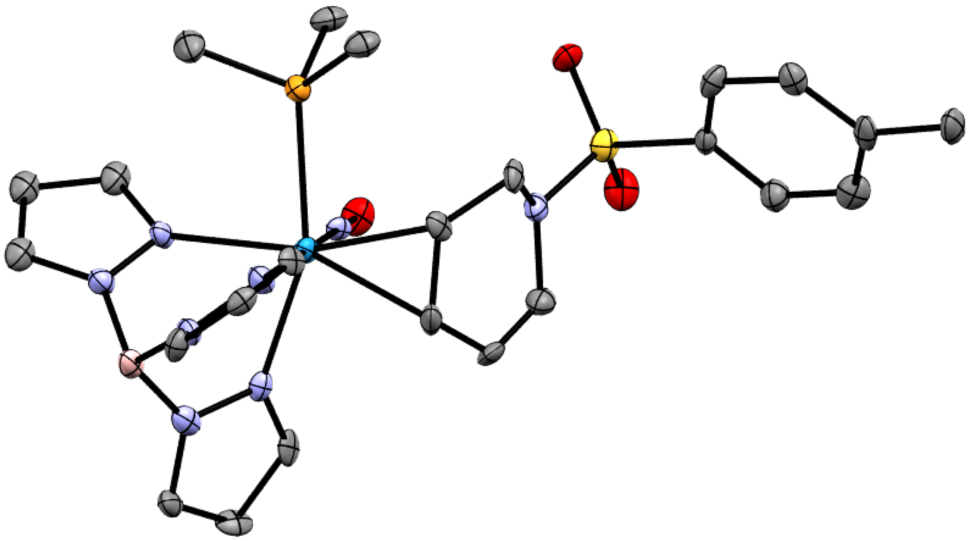

Figure S36: ORTEP/ellipsoid diagram of **13P**.

Table S4: SC-XRD data for **13P**.

CCDC<br>2323734	Chemical Formula<br>$C_{24}H_{32}BN_8O_3PSW$	FW (g/mol)<br>738.26
T (K)<br>100(2)	**λ** (Å)<br>0.71073	Crystal size (mm)<br>0.034 x 0.055 x 0.258
Crystal habit<br>Yellow rod	Crystal system<br>Triclinic	Space group<br>P -1
a (Å)<br>8.7191(10)	b (Å)<br>12.0085(15)	c (Å)<br>13.9368(16)
α (°)<br>96.925(4)	β (°)<br>90.003(3)	γ (°)<br>103.280(4)
V ($Å^3$)<br>1409.3(3)	Z<br>2	ρ_{calc} (g/cm^3)<br>1.740
μ (mm^{-1})<br>4.272	F(000)<br>732	θ range (°)<br>1.47 to 25.83
Index ranges<br>$-10 \leq h \leq 10$<br>$-14 \leq k \leq 14$<br>$-17 \leq l \leq 17$	Data/restraints/parameters<br>5384 / 0 / 355	Goodness-of-fit on F^2<br>1.005
R_1 [I>2σ(I)]<br>0.0432	wR_2 [all data]<br>0.0761	

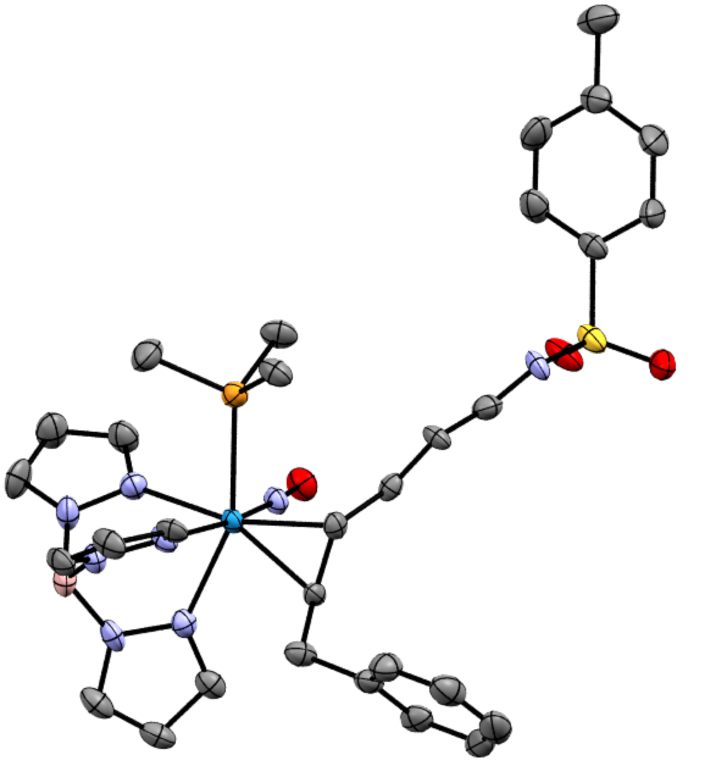

Figure S37: ORTEP/ellipsoid diagram of **17D**.

Table S5: SC-XRD data for **17D**.

CCDC<br>2323735	Chemical Formula<br>$C_{31}H_{38}BN_8O_3PSW$	FW (g/mol)<br>828.38
T (K)<br>100(2)	**λ** (Å)<br>0.71073	Crystal size (mm)<br>0.022 x 0.025 x 0.124
Crystal habit<br>Yellow needle	Crystal system<br>Monoclinic	Space group<br>P 1 21/c 1
a (Å)<br>12.9854(7)	b (Å)<br>13.6574(8)	c (Å)<br>19.8319(13)
α (°)<br>90	β (°)<br>100.964(2)	γ (°)<br>90
V (Å^3)<br>3452.9(4)	Z<br>4	ρ_{calc} (g/cm^3)<br>1.594
μ (mm^{-1})<br>3.497	F(000)<br>1656	θ range (°)<br>1.82 to 26.40
Index ranges<br>$-15 \leq h \leq 16$<br>$-16 \leq k \leq 17$<br>$-24 \leq l \leq 23$	Data/restraints/parameters<br>7067 / 0 / 423	Goodness-of-fit on F^2<br>0.995
R_1 [I>2σ(I)]<br>0.0406	wR_2 [all data]<br>0.0883	

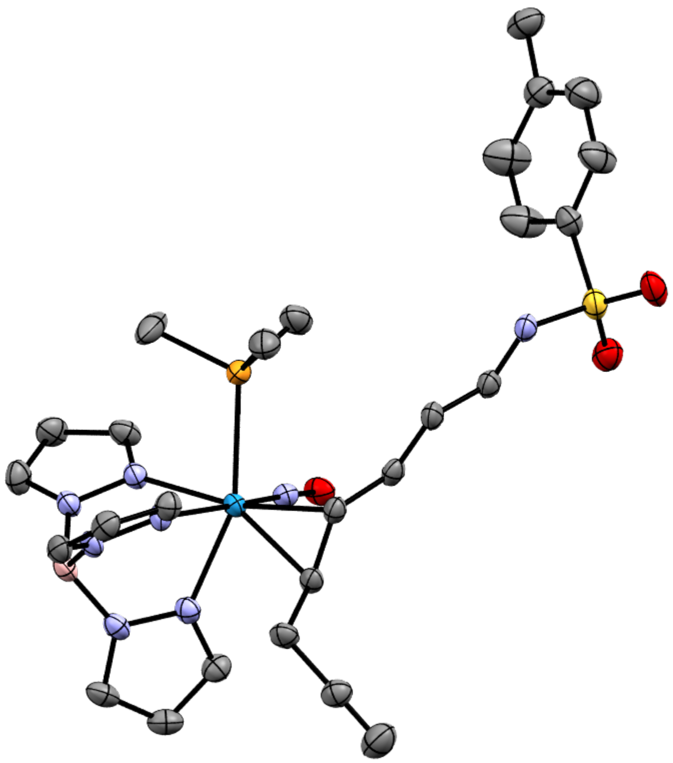

Figure S38: ORTEP/ellipsoid diagram of **19D**.

Table S6: SC-XRD data for **19D**.

CCDC	Chemical Formula	FW (g/mol)
2324410	$C_{27}H_{34}BN_8O_3PSW$	776.31
T (K)	**λ** (Å)	Crystal size (mm)
100(2)	0.71073	0.031 x 0.059 x 0.110
Crystal habit	Crystal system	Space group
Orange plate	Monoclinic	$P\ 2_1/n$
a (Å)	b (Å)	c (Å)
10.2794(8)	21.5308(12)	14.1410(10)
α (°)	β (°)	γ (°)
90	93.927(2)	90
V (Å^3)	Z	ρ_{calc} (g/cm^3)
3122.4(4)	4	1.651
μ (mm^{-1})	F(000)	θ range (°)
3.860	1544	2.20 to 26.39
Index ranges	Data/restraints/parameters	Goodness-of-fit on F^2
$-12 \leq h \leq 12$<br>$-25 \leq k \leq 26$<br>$-17 \leq l \leq 17$	6391 / 0 / 387	1.012
R_1 [I>2σ(I)]	wR_2 [all data]	
0.0296	0.0644	

Computational Methods.

Ground-state structures were optimized at the M06 level of theory using the 6-31G** [LANL2DZ for W] basis set in Gaussian 16. Previous literature demonstrates that this functional and basis set choice accurately corroborates experimental results. Solvent effects of acetonitrile were modeled using SMD. Gaussian's default criteria was used for optimization, vibrational frequency analysis verified that structures were minima and thermal free energy corrections were applied.

Table S7.

Structure	Compound	Electronic Energy (Hartree)	Relative Free Energy (kcal/mol)
6D	Organic	-1107.027080	0.00
16D		-1107.025930	+0.76
6D - s	Metal Bound	-2467.419617	0.00
16D		-2467.424033	-2.77
6D – s	R = Methyl	-2467.419617	0.00
6D – a		-2467.406407	+8.29
6D - s	R = t-Butyl	-2585.186730	0.00
6D – a		-2585.161620	+15.76
